# Supplementary material for: The STAIR OF KNOWLEDGE—a codesigned intervention to prevent pressure ulcers, malnutrition, poor oral health and falls among older persons in nursing homes in Sweden: development of a complex intervention
Source: BMJ Open. 2023 Aug 10;13(8):e072453. doi: 10.1136/bmjopen-2023-072453 (PMC10423781; doi:10.1136/bmjopen-2023-072453)
Supplement: Author's manuscript [file bmjopen-2023-072453.draft_revisions.pdf]

BMJ Open is committed to open peer review. As part of this commitment we make the peer review history of every article we publish publicly available.

When an article is published we post the peer reviewers' comments and the authors' responses online. We also post the versions of the paper that were used during peer review. These are the versions that the peer review comments apply to.

The versions of the paper that follow are the versions that were submitted during the peer review process. They are not the versions of record or the final published versions. They should not be cited or distributed as the published version of this manuscript.

BMJ Open is an open access journal and the full, final, typeset and author-corrected version of record of the manuscript is available on our site with no access controls, subscription charges or pay-per-view fees (http://bmjopen.bmj.com).

If you have any questions on BMJ Open's open peer review process please email info.bmjopen@bmj.com

BMJ Open

Development of a codesigned complex intervention to prevent the risks of pressure ulcers, malnutrition, poor oral health and falls among older persons in nursing homes in Sweden - The STAIR OF KNOWLEDGE

Journal:	*BMJ Open*
Manuscript ID	bmjopen-2023-072453
Article Type:	Original research
Date Submitted by the Author:	02-Feb-2023

Complete List of Authors:	Neziraj, Merita; Malmo University, Department of Care Science, Faculty of Health and Society<br>Axelsson, Malin; Malmö University, Department of Care Science, Faculty of Health and Society<br>Kumlien, Christine ; Malmo University, Department of Care Science, Faculty of Health and Society; Skanes universitetssjukhus Malmo, Department of Cardio-Thoracic and Vascular Surgery, Skane University Hospital, Malmö, Sweden<br>Hellman, Peter; Malmo University, Department of Care Science, Faculty of Health and Society<br>Andersson, Magdalena; Unit of Research and development and competence centre, City of Malmö, Sweden, Health and Social Care, Strategic Development

Keywords:	PREVENTIVE MEDICINE, GERIATRIC MEDICINE, Aging

SCHOLARONE™
Manuscripts

I, the Submitting Author has the right to grant and does grant on behalf of all authors of the Work (as defined in the below author licence), an exclusive licence and/or a non-exclusive licence for contributions from authors who are: i) UK Crown employees; ii) where BMJ has agreed a CC-BY licence shall apply, and/or iii) in accordance with the terms applicable for US Federal Government officers or employees acting as part of their official duties; on a worldwide, perpetual, irrevocable, royalty-free basis to BMJ Publishing Group Ltd ("BMJ") its licensees and where the relevant Journal is co-owned by BMJ to the co-owners of the Journal, to publish the Work in this journal and any other BMJ products and to exploit all rights, as set out in our licence.

The Submitting Author accepts and understands that any supply made under these terms is made by BMJ to the Submitting Author unless you are acting as an employee on behalf of your employer or a postgraduate student of an affiliated institution which is paying any applicable article publishing charge ("APC") for Open Access articles. Where the Submitting Author wishes to make the Work available on an Open Access basis (and intends to pay the relevant APC), the terms of reuse of such Open Access shall be governed by a Creative Commons licence – details of these licences and which Creative Commons licence will apply to this Work are set out in our licence referred to above.

Other than as permitted in any relevant BMJ Author's Self Archiving Policies, I confirm this Work has not been accepted for publication elsewhere, is not being considered for publication elsewhere and does not duplicate material already published. I confirm all authors consent to publication of this Work and authorise the granting of this licence.

For peer review only

Title

Development of a codesigned complex intervention to prevent the risks of pressure ulcers, malnutrition, poor oral health and falls among older persons in nursing homes in Sweden - The STAIR OF KNOWLEDGE

Merita Neziraj [1]

Email: merita.neziraj@mau.se

Malin Axelsson[1]

Email: malin.axelsson@mau.se

Christine Kumlien[1, 2]

Email: christine.kumlien@mau.se

Peter Hellman[1]

Email: peter.hellman@mau.se

Magdalena Andersson[3]

Email: magdalena.andersson@malmo.se

[1]*Department of Care Science, Faculty of Health and Society, Malmö University, Sweden*

[2]*Department of Cardio-Thoracic and Vascular Surgery, Skane University Hospital, Malmö, Sweden*

[3]*Department of Health and Social Care, Strategic Development, Unit of Research and development and competence centre, City of Malmö, Sweden*

Corresponding Author: Merita Neziraj. merita.neziraj@mau.se

For peer review only - http://bmjopen.bmj.com/site/about/guidelines.xhtml

Abstract

Objectives: To describe the development of a codesigned complex intervention intended to prevent the risks of pressure ulcers, malnutrition, poor oral health and falls among older persons in nursing homes.

Design: This part of a two-arm pragmatic cluster randomized controlled trial focused on developing a codesigned complex intervention. The development of the current intervention was inspired by the Medical Research Council guidelines for complex interventions and the action part of the knowledge-to-action framework and was intended to work in a real-world setting.

Setting: Nursing homes in southern Sweden.

Participants: Nurse aides, registered nurses and managers in nursing homes codesigned the intervention together with the research group in workshops. Key persons working in the municipality who were considered to play an important role in the development of the intervention also participated throughout this process in a dynamic and iterative way.

Results: The codesigned intervention, the STAIR OF KNOWLEDGE, is intended to bridge the evidence-practice gap regarding the entire preventive care process of the risks of pressure ulcers, malnutrition, poor oral health and falls among older persons in nursing homes. The intervention is aimed at nurse aides, registered nurses and managers. The intervention lasts for three weeks and is divided into two parts. In part one, nurse aides, registered nurses and managers obtain knowledge on their own by following written instructions. In part two, they meet, interact and discuss the knowledge acquired during part one.

Conclusion: The current codesign complex intervention, the STAIR OF KNOWLEDGE, which aims to prevent the risks of pressure ulcers, malnutrition, poor oral health and falls among older persons in nursing homes, is robustly developed and thoroughly described. The description of the entire development process may enhance the replicability of this intervention. The intervention needs to be tested and evaluated in an upcoming feasibility study.

Trial number: Clinical Trial NCT05308862.

For peer review only

Strengths

- Inspired by the Medical Research Council guidelines for complex intervention, a robust development process was undertaken based on the literature and research conducted in the local context prior to developing the complex intervention.
- A complex intervention was codesigned both with and for nurse aides, registered nurses and managers in workshops. Additionally, key persons working in the municipality were engaged in the development of this tailored intervention.
- To bridge the evidence-practice gap regarding the risks of pressure ulcers, malnutrition, poor oral health and falls among older persons in nursing homes, knowledge translation strategies were applied during the development process in accordance with the action part of the knowledge-to-action framework.
- A thorough description of the entire development process may enhance the replicability of the current intervention.

Limitations

- One limitation of the development process was that this design is time- and resource-consuming. On the other hand, this was necessary to develop a tailored complex intervention that might enhance the likelihood of successful implementation.

For peer review only

Introduction

There remains an evidence-practice gap in preventing the risks of pressure ulcers, malnutrition, poor oral health and falls among older persons in nursing homes[1 2]. These health risks cause a major burden for older persons[3] and they are costly for the health care system[4]. Since older persons are more vulnerable to these health risks[5] and considering the increasing ageing population globally, particularly with regard to older persons aged 80 years or older[6], evidence-based preventive work is crucial to manage this demographic challenge and, importantly, these health risks among older persons.

In Sweden, there is a national quality register, Senior Alert, providing an individualized, standardized, structured and systematic preventive care work process for older persons 65 years or older who are at risk of pressure ulcers, malnutrition, poor oral health and falls[7]. Senior Alert provides evidence-based knowledge aimed at preventing these health risks to enable a healthy ageing among older persons[8]; in addition, it can increase cost efficiency[9]. However, a lack of knowledge among those working with older persons has been identified as one major challenge regarding to preventive work[2 10]. As a result, these health risks continue to be prevalent[7]. For instance, approximately every third older person living in a nursing home faces at least one of these health risks, and every tenth older person faces all four of these health risks[1]. Additionally, not all older persons who are at risk have planned care interventions[11 12,] and there is a mismatch between identified risks and planned and performed care interventions[13 14], thus indicating an evidence-practice gap and consequently, highlighting the urgent need of translating knowledge into practice.

Nevertheless, this is not unique to Sweden or this context; in contrast, health systems worldwide face the shared challenge of translating knowledge into practice[15]. Knowledge translation has been defined as *"a dynamic and iterative process that includes synthesis, dissemination, exchange and ethically sound application of knowledge to improve health care of people in the country, provide more effective health service and products and strengthen the health care system",* p. 165[16]. Ineffective knowledge translation can result in an evidence-practice gap[17] and, worryingly, lead to situations in which patients are denied interventions that have been proven to be beneficial[18], which in turn can result in a reduction in their quality of life[19].

For peer review only - http://bmjopen.bmj.com/site/about/guidelines.xhtml

To bridge this evidence-practice gap, conceptual frameworks are recommended[20]. The knowledge-to-action (KTA) framework is intended to help the parties involved in the process of knowledge translation[18]. The KTA framework is also appropriate when addressing an evidence-practice gap[15] and conducting pragmatic research[18].

As a part of translating knowledge into practice and promoting knowledge use by end users[21], the engagement of both researchers and stakeholders in research is crucial[22]. Engaging stakeholders at an early stage in the development of solutions that can be applied to real world settings is essential according to the Medical Research Council´s (MRC) framework for complex interventions[23]. Complex interventions have multiple components, target multiple groups or levels of an organization and attempt to affect multiple outcomes[23]. Additionally, for complex interventions to be most useful to end users, the local context must be taken into account[24]. Since it is well underpinned that organizational factors hinder preventive work in nursing homes[2 25], considering and understanding the local context and integrating it into the process of intervention development is crucial[26].

Consequently, change in the practices of nursing homes is considered to be complex[27], but if complex interventions are tailored to the local context[28], including the targets of the intervention[23 24] and is directly relevant to them[29,] such interventions could be successful.

Aim

The aim of this article is to describe the development of a codesigned complex intervention intended to prevent the risks of pressure ulcers, malnutrition, poor oral health and falls among older persons in nursing homes.

Methods

Definitions

Nursing homes were defined based on the definition provided by Neziraj et al. (2021)[1]: older persons receiving municipal health care in residential care homes.

Health care personnel and managers were defined based on the definition provided by Neziraj et al. (2021) as follows[2]:

Nurse aide: a person with a secondary degree in nursing, involves three years of study in high school *or* a person without any formal education in nursing.

For peer review only - http://bmjopen.bmj.com/site/about/guidelines.xhtml

Registered nurse: a person with a bachelor's degree in nursing, which involves three years of study at university.

Manager: a person who is in charge of nurse aides or registered nurses.

End-users: nurse aides, registered nurses and managers working in nursing homes.

Stakeholders: key persons working in the municipality who are considered to play an important role in the development and implementation of the intervention.

Study context and setting

In nursing homes, nurse aides are the main providers of care and services and are on duty around the clock. Nurse aides work under the regulations of the Social and Services Act (SFS)[30] but are also delegated tasks according to the Health and Medical Services Act (HSL)[31], usually by registered nurses. Registered nurses guide care in nursing homes and work under the regulations of HSL[31]. In the current setting, a large town located in southern Sweden, one registered nurse (or occasionally more depending on the size of the nursing home) is located in the nursing home during office hours but is also available at any other time. Managers who are in charge of the care and services provided by the nurse aides are located at their respective nursing homes during office hours.

Study design

This part of the PROSENIOR project and a two-arm pragmatic cluster randomized controlled trial focused on the development of a codesigned complex intervention. The development of the current complex intervention (hereafter called simply the intervention) was conducted in a pragmatic paradigm as it is intended to work in a real-world setting [29]; this process was inspired by the MRC guidelines for complex interventions[24], applied the KTA framework[18] and engaged end-users and stakeholders in the process of codesign[32].

We follow the guidance for reporting intervention development studies (GUIDED)[33] when describing the development of the intervention and the template for intervention description and replication (TIDieR) checklist and guide[34] when describing the intervention. We use "development" to refer to the whole process of intervention development and "design" to indicate the intervention content, format and delivery.

Patient and public involvement

For peer review only - http://bmjopen.bmj.com/site/about/guidelines.xhtml

Patients were not involved in the research process. End users codesigned the intervention with the research group in workshops. Stakeholders were also involved in this research; they supported the research group throughout the entire development of the intervention by contributing their valuable knowledge. All engagement is described in detail in the section "Development of the intervention" below.

Development of the intervention

We developed the intervention in three phases and applied the KTA framework in all phases (Figure 1).

Theory

The KTA framework takes implementation strategies into account already in the development phase[18], which promotes and sustains practice change[15]. We applied the KTA framework because it offers a structured and systematic approach to translate knowledge into practice[18]. It comprises two parts: knowledge creation and the action cycle. Since evidence-based knowledge is already available to end users in the quality register Senior Alert, the action cycle was applied during the development of the current intervention. The action cycle consists of the following steps: *1. Identify the problem, identify and review selected knowledge, 2. Adapt knowledge to the local context, 3. Assess barriers to knowledge use, 4. Select and tailor implementation strategies, 5. Monitor knowledge use, 6. Evaluate the outcomes* and *7. Sustain knowledge use*[18 35]. Steps 1-4 the action cycle were applied throughout the development process of developing the intervention in an iterative, dynamic and permeable way.

Phase one

During this phase, we established contact with stakeholders in the municipality, searched for relevant literature and conducted studies in the local context.

Establishing contact with stakeholders in the municipality

Initially, we established contact and met with the head of the nursing homes in the municipality. In addition, a reference group was created, which consisted of experts drawn from the local context.

Searching for literature and conducting studies in the local context

For peer review only - http://bmjopen.bmj.com/site/about/guidelines.xhtml

As a part of step 1 in the KTA framework*, identify the problem, identify and review selected knowledge*, we first searched for literature regarding the risks of pressure ulcers, malnutrition, poor oral health and falls and intervention studies in this area (unpublished project plan). Subsequently, we conducted a cross-sectional study to determine the prevalence of the risks of pressure ulcers, malnutrition, poor oral health and falls in nursing homes in southern Sweden[1].

As a part of steps 2-3 in the KTA framework, *adapt knowledge to the local context and assess barriers to knowledge use,* we conducted focus group interviews with end users who worked in nursing homes to prevent pressure ulcers, malnutrition, poor oral health and falls[2]. A detailed description of this study and its participants is provided in Neziraj et al[2]. Additionally, we asked the end users included in this study how an optimal intervention could be designed to prevent the risks of pressure ulcers, malnutrition, poor oral health and falls among older persons in nursing homes. These data were specifically targeted at the current part of the trial and were therefore not included in our published study, i.e., Neziraj et al[2].

Phase one suggested that individuals working with older persons in nursing homes need increased knowledge concerning how to prevent these health risks. Since existing evidence and knowledge concerning how to prevent these health risks is already contained in Senior Alert, the challenge seems to lie in the evidence-practice gap, which highlights the need for support to be provided to end users with regard to translating evidence into practice[1 2].

Phase two

During this phase, we recruited and randomized nursing homes, invited end users in the intervention arm to participate in workshops, and planned and conducted the workshops. We also analysed the specific data regarding intervention design drawn from the focus group interviews (see the previous paragraph on phase one for clarification) and the workshops.

Recruiting and randomizing nursing homes

Inclusion criteria for the trial were nursing homes working with and registered in the quality register Senior Alert. We recruited eligible nursing homes (n=21) to participate in the trial via digital meetings. In total, eight nursing homes agreed to participate and were cluster randomized using a computerized program to either the intervention (n=4) or control arm (n=4). The nursing home is the cluster and the unit of allocation. Due to the nature of the design, the cluster randomization of nursing homes was unblinded. Subsequently, we invited

For peer review only

end users (n=118) working in nursing homes in the intervention arm to participate in workshops intended to develop a tailored intervention together with the research group; the invitations were extended both via a digital information video and in written form. The remaining end users (n=184) working in the nursing homes who were allocated to the control arm continued with their usual care routine (Figure 2).

Conducting workshops

As a part of steps 2-4 in the KTA framework, *adapt knowledge to the local context, assess barriers to knowledge use* and *select and tailor implementation strategies*, we conducted workshops with end users. In total, four workshops were conducted, which featured two nurse aides, one registered nurse and one manager in each workshop; the workshops were conducted over the course of four weeks (March-April 2022). A majority of the end users (n=16) were women (n=13), between the ages of 28-63 years (mean 53), and had worked for 3-41 years (mean 18). The workshops were kept small to offer the end users the possibility of exhibiting activity and creativity[36]. The first author (MN) led the workshops together with one of the coauthors (all coauthors participated in one workshop each). The workshops were intended to serve as a place in which participants could learn together and discuss the design of the intervention in four different stations (Table 1). The end users engaged in active discussion and wrote creative ideas and suggestions on the walls and the board in a lecture hall designed for the purpose of encouraging creative pedagogy (Figure 3). In the first station, the end users were asked to discuss the risks of pressure ulcers, malnutrition, poor oral health and falls and the care interventions that should be applied. In the second station, they were asked to discuss and identify barriers and facilitators they had encountered in their own work regarding the preventive care process stipulated by Senior Alert (identify a risk, assess causes and plan, undertake and evaluate care intervention). Barriers were written down on pink post-it notes, while facilitators were written down on green post-it notes. These post-it notes were subsequently placed at the appropriate location on the board with regard to the predawn preventive care process. The focus of the discussions at station three was on the end-users´ needs and the support they needed throughout the preventive care process. In the fourth station, they were asked to discuss the core components of the intervention, how to provide follow-ups and implementation strategies. After completing each workshop, MN photographed and briefly summarized the written data from each station. This summary was used if the end users in the subsequent workshop reached an impasse and/or discussed and

For peer review only - http://bmjopen.bmj.com/site/about/guidelines.xhtml

Table 1. Workshop content (n=4).

Workshop	Station	Content	Examples of questions to discuss
Workshop 1-4	Station 1	Case regarding an older person at risk of pressure ulcers, malnutrition, poor oral health and falls living in a nursing home	• What would you have done in this case regarding these four risks?<br>• Are there any good examples? What can you learn from good examples?<br>• What additional knowledge do you need regarding these four risks in order to produce a risk assessment and provide adequate care interventions?
	Station 2	Senior Alert´s care process	• Place green/pink post-it notes on the care process regarding what works/what can be improved in your own work and workplace.
	Station 3	End-users needs´ and the support they need regarding preventive work	• What do you need in your preventive work?<br>• Why is this important, and what is most important (rank 1-3)?<br>• Who needs help in the context of preventive work?<br>• Who should be involved and in what way?<br>• What is necessary for it to be feasible?<br>• How can you work better/smarter?<br>• How can you work in a more sustainable way?
	Station 4	Core components of the intervention	• What should be included in the intervention?<br>• Who should it target?<br>• How should it be designed?<br>• How much/often/for how long should the intervention take place?<br>• How should it be followed up?<br>• Where should it be implemented?<br>• How should it be implemented?

For peer review only

For peer review only - http://bmjopen.bmj.com/site/about/guidelines.xhtml

wrote similar suggestions and ideas to those proposed by the end users in the previous workshop. Each workshop lasted for three hours, and the discussions were audio recorded to support the written data collection during the analysis.

Analysing the data from the focus group interviews and the workshops

The analysis was guided by the six phases of reflexive thematic analysis described by Braun and Clarke[37 38]: *1. Familiarizing with the data, 2. Coding, 3. Generating initial themes, 4. Reviewing the identified themes, 5. Defining and naming the themes* and *6. Producing the report.* Thematic analysis was chosen because it facilitates a flexible analysis process but simultaneously provides researchers with the core skills they need to conduct the analysis.

To familiarize ourselves with the data, MN and MA read the transcripts from the focus group interviews, including the data specifically collected for the current part of the trial, and the written data collected from the workshops. In addition, MN listened to all the audio-recorded discussions from the workshops meticulously. During the process of reading the data, MN and MA reflected on and generated initial codes. Subsequently, MN and MA met and discussed these initial codes (1). Thereafter, MN and MA separately engaged in a process of identifying and coding entities of interest in relation to the design of the intervention, giving equal attention to all the data (2). The initial codes were then sorted into their core components in relation to the design of the intervention (3). Next, the core components were reviewed by MN to determine whether any relevant data regarding the design of the intervention had been missed (4). Subsequently, MN designed an outline of the intervention. This outline contained the intervention's proposed design, including its content, format, plan for delivery and duration. In the following step of the analysis, the entire research group met and discussed the design of the outline of the intervention. During this step, MN continuously revised the outline of the intervention following discussions within the research group (5). Then, the outline of the intervention was redesigned by MN. The redesigned outline of the intervention was then presented to the research group before it was presented to the stakeholders. The process of producing the final design of the intervention is described in phase three below (6).

Phase three

As part of steps 2-4 in the KTA framework, *adapt knowledge to the local context, assess barriers to knowledge use* and *select and tailor implementation strategies,* MN and MA met regularly with stakeholders to present and discuss the outline of the intervention. MA works

For peer review only - http://bmjopen.bmj.com/site/about/guidelines.xhtml

within the municipality and thus facilitated contact with stakeholders who were considered to play an important role in this part of designing the intervention. Since this part of the process was dynamic and iterative and because all relevant uncertainties had not been addressed in the redesigned outline of the intervention, it was helpful to meet stakeholders for the purpose of identifying and addressing the remaining uncertainties regarding the content, format, delivery and duration of the intervention. This part of the process was time-consuming and required a back-and-forth process involving meetings and discussions between MN and MA, within the entire research group and with the stakeholders. Next, the redesigned outline of the intervention was adjusted by MN in accordance with the results of these meetings and discussions (Figure 4). Finally, MN investigated whether any data from the focus group interviews and the workshops had been missed, since these data were intended to serve as the foundation for designing the final outline of the intervention. The final outline of the intervention, the STAIR OF KNOWLEDGE (Figure 5), is described below.

Results

The final design of the intervention

The STAIR OF KNOWLEDGE consists of *the foundation and stairs 1-6,* lasts for three weeks and is divided into two parts. Part one, including *the foundation and stairs 1-5*, takes place throughout the entire intervention period (weeks 1-3) and is delivered digitally to end users in the nursing homes via their workplace email addresses. Part two includes *stair 6* and takes place during the last week of the intervention period (week 3) in the nursing homes in question.

In part one, *the foundation and stairs 1-5*, end users obtain knowledge on their own by following written instructions. *The foundation and stairs 1-5* provide end users with website links that allow them to both read texts and watch videos. *The foundation* provide end users with knowledge and awareness of how to work preventively in the context of an existing local working routine and is intended to represent "the ground to stand on". *Stairs 1-4* provide the end users with general knowledge drawn from the quality register Senior Alert regarding the risks of pressure ulcers, malnutrition, poor oral health and falls *(stair 1),* risk assessment instruments *(stair 2),* the underlying causes of these risks *(stair 3)* and preventive care interventions (*stair 4)*. *Stairs 1-4* are mandatory for all professionals. S*tair 5* provides end users with knowledge of how to register in the quality register Senior Alert and is mandatory

For peer review only - http://bmjopen.bmj.com/site/about/guidelines.xhtml

only for users who have access to and the responsibility to register in the quality register Senior Alert.

In part two, *stair 6*, MN meets with end users to interact with them and discuss the knowledge acquired during part one. Part two is intended to inspire end users to prevent the risks of pressure ulcers, malnutrition, poor oral health and falls. MN moderates the sessions, which last approximately 30 minutes each and are held Monday to Friday in the nursing homes in question based on the content of part one. In these sessions, end users are, for instance, asked to perform risk assessments of different cases, identify the underlying causes and plan for accurate care interventions. End users are also asked to identify environmental risk factors related to the risks of pressure ulcers, malnutrition, poor oral health and falls in their own workplace and to discuss and generate ideas concerning how to follow up on the preventive care process at the organizational level.

Discussion

The current codesigned complex intervention, the STAIR OF KNOWLEDGE, was developed together with end users in workshops in an active and creative way. Stakeholders were also engaged in an iterative and dynamic way throughout the development of the intervention, as an important part of undertaking implementation strategies already in the development phase[39]. As recommended by the MRC framework[24], we meticulously considered the relationship between the intervention and its context when developing the intervention. Furthermore, we followed the strategies for knowledge translation included in the KTA framework[18]. Hence, the strengths exhibited by the development of this complex intervention lie in the fact that it was developed both together with and for end users and engaged stakeholders who are considered to play an important role in the development and implementation process. The current intervention is intended to work in a real-world setting and aims to bridge the evidence-practice gap regarding the process of preventing the risks of pressure ulcers, malnutrition, poor oral health and falls; ultimately, this intervention may reduce these risks among older persons in nursing homes.

The benefits of codesign are potentially substantial[40]. For instance, engaging end users and stakeholders as design partners to the research group could ensure that the intervention exhibits a better fit to their needs[32]. Engaging end users and stakeholders early enables their experiences and requirements to be taken into account at the start rather than a situation in which the researchers presume to know what is required[39]. In the current development

For peer review only - http://bmjopen.bmj.com/site/about/guidelines.xhtml

process, although end users´ and stakeholders´ engagement ranged in intensity from relatively passive to highly active, their engagement pervaded the entire development process, and important decisions regarding the intervention design were made by considering their input. Furthermore, because we engaged end users and stakeholders, the current intervention was based on their own experiences regarding the evidence and knowledge that are necessary throughout the entire process of preventing the risks of pressure ulcers, malnutrition, poor oral health and falls. Engaging end users and stakeholders during the developing process[41] was also important in light of the local context since this enabled us to identify facilitators and barriers in the environment in which the intervention will eventually be implemented[26].

Considering and understanding the local context is also crucial when addressing an evidence-practice gap[24]. In this case, knowledge concerning the process of preventing the risks of pressure ulcers, malnutrition, poor oral health and falls is already contained in the quality register Senior Alert, but this evidence has not been fully translated into practice. Thus, we focused on translating the existing knowledge contained in Senior Alert into practice. However, if this knowledge is to be implemented effectively[42], it is crucial to employ a conceptual framework[20]. Therefore, we chose the KTA framework because it provided us with knowledge translation strategies to reduce the evidence-practice gap[18], and it was suitable since the quality register Senior Alert is already in use. Furthermore, adapting knowledge to the local context and assessing barriers to knowledge use may enable the research to have a greater impact[43], which could in turn reduce the evidence-practice gap.

Successful intervention development is characterized as rigorous and scientific and leads to an intervention that can be implemented in a real-world setting[33]. The robust research process used to develop the STAIR OF KNOWLEDGE intervention incorporates existing evidence, the views of end users and stakeholders[40], the local context and knowledge translation strategies. Consequently, the use of knowledge translation strategies and the engagement of end users who are embedded in the local context in the development of a tailored complex intervention both for and with them could contribute to increased knowledge and awareness of the entire process of preventive care. This may, in turn, reduce the evidence-practice gap among end users and, importantly, reduce the risk of pressure ulcers, malnutrition, poor oral health and falls among older persons in nursing homes. Furthermore, the engagement of stakeholders already in the development process is likely to facilitate the implementation of the current intervention.

For peer review only - http://bmjopen.bmj.com/site/about/guidelines.xhtml

Limitations

Although the development of this complex intervention has been completed, it is important to acknowledge the limitations of the development process. First, only four clusters were included in the development process. Nevertheless, since this part of the trial focused on the development of an intervention rather than its evaluation and because the clusters were recruited pragmatically, the clusters included in the trial could be considered sufficient. Second, although all end users in the intervention arm (n=118) were invited to participate in workshops, only 16 participated. However, different professionals participated in the workshops, and the discussions were energetic, active and creative. Third, although this design is creative and can generate new ideas, it is time- and rescore-consuming for all parties involved. It requires end users and stakeholders to set aside time and expend extra effort in their daily work. For researchers, this process requires careful planning to enable them to coordinate, meet with many different persons repeatedly and be responsive to all parties involved. However, although this design required the expenditure of time and resources, the engagement of end users, stakeholders and researchers is meaningful and necessary to develop successful interventions; ultimately, this design might have an impact on to prevent the risks of pressure ulcers, malnutrition, poor oral health and falls among older persons in nursing homes. Furthermore, the current intervention might offer value when used by others and could likely be adjusted to and tested in similar contexts.

Conclusion

The current codesign complex intervention, the STAIR OF KNOWLEDGE, which aims to prevent the risks of pressure ulcers, malnutrition, poor oral health and falls among older persons in nursing homes, is robustly developed and thoroughly described. A careful description of the entire development process and the intervention itself can enhance the replicability of the current intervention. This article highlights the extensive process that is necessary for the development of tailored complex interventions. Finally, this codesigned complex intervention might result in more evidence-based practice concerning the entire process of preventing the risks of pressure ulcers, malnutrition, poor oral health and falls and, importantly, reduce these health risks among older persons in nursing homes. However, uncertainties regarding the intervention itself remain. Thus, the STAIR OF KNOWLEDGE must be tested and evaluated in an upcoming feasibility study before we continue to the stage of conducting a full trial evaluation.

For peer review only - http://bmjopen.bmj.com/site/about/guidelines.xhtml

Ethical considerations

This trial was approved by the Swedish Ethical Review Authority (DNR 2019-06414). In addition, written approval was requested and granted by the head of the department of elderly care homes in the municipality in which this trial was conducted. All end users working in eligible nursing homes were invited to participate in the workshops. Moreover, end users had the right to withdraw from participation at any stage without providing reasons and bearing any consequences. Participation in the workshops was based on written consent. The results of this trial may be considered to contribute to scientific value on good ethical grounds, and the benefits of participating in the trial outweigh the corresponding risks.

Funding statement

This research received finical support with regard to conducting the workshops from the Derbrings and Stöltens research and development foundation. The funders had no role in the research manuscript's design, conduct, analysis, interpretation or drafting.

Authors' contributors

All the authors contributed to this part of the trial in terms of conception, planning and design. MN coordinated the workshops and meetings with stakeholders. MN led all the workshops, and all coauthors participated in one workshop each. MN and MA analysed the data collected from the focus group interviews (i.e., the data that were specifically collected for inclusion in the current part of the trial) and the workshops. All authors interpreted the results. MN drafted the design of the intervention, and all coauthors revised the intervention in regard to its design. MN drafted the manuscript, and all coauthors supervised and revised the manuscript with respect to important intellectual content. All the authors read and approved the final version of the manuscript.

Competing interests

The authors have no conflicts of interest.

Participant consent

All participation in the workshops was based on written consent.

Acknowledgements

For peer review only

We thank the nurse aides, registered nurses and managers who participated in the development of the intervention. We also thank the stakeholders who participated during this process. Their enthusiastic participation was helpful in developing the STAIR OF KNOWLEDGE intervention. The Derbring and Stölten foundation is acknowledged for its financial support.

Data sharing statement

The data that support the development of the STAIR OF KNOWLEDGE intervention are not publicly available to ensure confidentiality. All data relevant to the development are included in the article. All figures and tables included in this article are original.

For peer review only

1. Neziraj M, Hellman P, Kumlien C, et al. Prevalence of risk for pressure ulcers, malnutrition, poor oral health and falls: a register study among older persons receiving municipal health care in southern Sweden. *BMC Geriatrics* 2021;21(1) doi: 10.1186/s12877-021-02205-x
2. Neziraj N, Andersson M, Hellman P, et al. Prevention of pressure ulcers, malnutrition, poor oral health and falls in nursing homes: A focus group study with nurse aides, registered nurses and managers. *International Journal of Nursing Studies Advances* 2021;3(100056-) doi: 10.1016/j.ijnsa.2021.100056
3. Gorecki C, Brown JM, Nelson EA, et al. Impact of pressure ulcers on quality of life in older patients: a systematic review. *Journal of the American Geriatrics Society* 2009;57(7):1175-83.
4. Marjolein van der V, Juanita AH, Geraerds AJLM, et al. Health care costs of injury in the older population: a prospective multicentre cohort study in the Netherlands. *BMC Geriatrics* 2020;20(1):1-11. doi: 10.1186/s12877-020-01825-z
5. Bravell ME, Westerlind B, Midlov P, et al. How to assess frailty and the need for care? Report from the Study of Health and Drugs in the Elderly (SHADES) in community dwellings in Sweden. *ARCHIVES OF GERONTOLOGY AND GERIATRICS* 2011;53(1):40-45. doi: 10.1016/j.archger.2010.06.011
6. WHO. Ageing well must be a global priority 2014 [accessed 10-23 2020.
7. Trinks A, Hägglin C, Dennis N, et al. The impact of a national quality register in the analysis of risks and adverse events among older adults in nursing homes and hospital wards: a Swedish senior Alertsurvey. *Safety in Health* 2018;4(10) doi: 10.1186/s40886-018-0077-x
8. Edvinsson J, Rahm M, Trinks A, et al. Senior alert: a quality registry to support a standardized, structured, and systematic preventive care process for older adults. *Quality management in health care* 2015;24(2):96-101.
9. Rubenstein LZ, Harker JO, Salvà A, et al. Screening for undernutrition in geriatric practice: developing the short-form mini-nutritional assessment (MNA-SF). *The journals of gerontology Series A, Biological sciences and medical sciences* 2001;56(6):M366-M72.
10. Dalvand S, Ebadi A, Ghanei Gheshlagh R. Nurses' knowledge on pressure injury prevention: a systematic review and meta-analysis based on the Pressure Ulcer Knowledge Assessment Tool. *Clinical, Cosmetic and Investigational Dermatology* 2018;ume 11:613-20.
11. Gusdal A, Johansson-Pajala R-M, Arkkukangas M, et al. Preventing Falls and Malnutrition among Older Adults in Municipal Residential Care in Sweden: A Registry Study. *SAGE Open Nursing* 2021;7 doi: 10.1177/23779608211026161
12. Backlund A, Holmbeck O, Kumlien C, et al. A registry study of nursing assessments, interventions and evaluations according to nutrition for persons living in municipal residential care homes. *Nursing open* 2018;5(3) doi: 10.1002/nop2.144
13. Witt S, Englander E, Kumlien C, et al. Mismatch between risk factors and preventive interventions? A register study of fall prevention among older people in one Swedish county. *International journal of older people nursing* 2018;13(4):e12209.
14. Hopewell S, Adedire O, Copsey BJ, et al. Multifactorial and multiple component interventions for preventing falls in older people living in the community. *Cochrane Database of Systematic Reviews* 2018(7) doi: 10.1002/14651858.CD012221.pub2
15. Field B, Booth A, Ilott I, et al. Using the Knowledge to Action Framework in practice: a citation analysis and systematic review. *Implement Sci* 2014;9:172. doi: 10.1186/s13012-014-0172-2 [published Online First: 2014/11/25]
16. Straus SE, Tetroe J, Graham I. Defining knowledge translation. *Cmaj* 2009;181(3-4):165-8. doi: 10.1503/cmaj.081229 [published Online First: 20090720]

For peer review only - http://bmjopen.bmj.com/site/about/guidelines.xhtml

17. Brownson RC, Eyler AA, Harris JK, et al. Getting the Word Out: New Approaches for Disseminating Public Health Science. *J Public Health Manag Pract* 2018;24(2):102-11. doi: 10.1097/phh.0000000000000673
18. Graham ID, Logan J, Harrison MB, et al. Lost in knowledge translation: time for a map? *J Contin Educ Health Prof* 2006;26(1):13-24. doi: 10.1002/chp.47 [published Online First: 2006/03/25]
19. Davis D, Evans M, Jadad A, et al. The case for knowledge translation: shortening the journey from evidence to effect. *Bmj* 2003;327(7405):33-5. doi: 10.1136/bmj.327.7405.33
20. Bowen S, Graham ID. Backwards design or looking sideways? knowledge translation in the real world Comment on "A call for a backward design to knowledge translation". *Int J Health Policy Manag* 2015;4(8):545-7. doi: 10.15171/ijhpm.2015.71 [published Online First: 20150324]
21. Eljiz K, Greenfield D, Hogden A, et al. Improving knowledge translation for increased engagement and impact in healthcare. *BMJ Open Qual* 2020;9(3) doi: 10.1136/bmjoq-2020-000983
22. Graham ID, Kothari A, McCutcheon C. Moving knowledge into action for more effective practice, programmes and policy: protocol for a research programme on integrated knowledge translation. *Implement Sci* 2018;13(1):22. doi: 10.1186/s13012-017-0700-y [published Online First: 20180202]
23. Craig P, Dieppe P, Macintyre S, et al. Developing and evaluating complex interventions: the new Medical Research Council guidance. *Bmj* 2008;337:a1655. doi: 10.1136/bmj.a1655 [published Online First: 2008/10/01]
24. Skivington K, Matthews L, Simpson SA, et al. A new framework for developing and evaluating complex interventions: update of Medical Research Council guidance. *BMJ-BRITISH MEDICAL JOURNAL* 2021;374:n2061. doi: 10.1136/bmj.n2061
25. Lannering C, Ernsth-Bravell M, Johansson L. Prevention of falls, malnutrition and pressure ulcers among older persons: nursing staff's experiences of a structured preventive care process. *Health & Social Care in the Community* 2017;25(3):1011-20. doi: 10.1111/hsc.12400
26. Beran D, Lazo-Porras M, Cardenas MK, et al. Moving from formative research to co-creation of interventions: insights from a community health system project in Mozambique, Nepal and Peru. *BMJ Global Health* 2018;3(6):e001183. doi: 10.1136/bmjgh-2018-001183
27. Rantz MJ, Flesner MK, Zwygart-Stauffacher M. Improving care in nursing homes using quality measures/indicators and complexity science. *J Nurs Care Qual* 2010;25(1):5-12. doi: 10.1097/NCQ.0b013e3181c12b0f [published Online First: 2009/12/17]
28. Nilsen P, Bernhardsson S. Context matters in implementation science: a scoping review of determinant frameworks that describe contextual determinants for implementation outcomes. *BMC Health Services Research* 2019;19(1):1-21. doi: 10.1186/s12913-019-4015-3
29. Zwarenstein M, Treweek S, Gagnier JJ, et al. Improving the reporting of pragmatic trials: an extension of the CONSORT statement. *BMJ* 2008;337:a2390. doi: 10.1136/bmj.a2390
30. SFS. The Social and Services Act (2001:453). Socialtjänstlag (2001:453) Svensk författningssamling 2001:2001:453 t.o.m. SFS 2022:1034 - Riksdagen Accessed January 31 2023.
31. HSL. The Health and Medical Services Act (2017:30). Hälso- och sjukvårdslag (2017:30) Svensk författningssamling 2017:2017:30 t.o.m. SFS 2022:1255 - Riksdagen Accessed 31 January 2023.
32. Slattery P, Saeri AK, Bragge P. Research co-design in health: a rapid overview of reviews. *Health Res Policy Syst* 2020;18(1):17. doi: 10.1186/s12961-020-0528-9 [published Online First: 20200211]
33. Duncan E, O'Cathain A, Rousseau N, et al. Guidance for reporting intervention development studies in health research (GUIDED): an evidence-based consensus study. *BMJ Open* 2020;10(4):e033516. doi: 10.1136/bmjopen-2019-033516 [published Online First: 20200408]
34. Hoffmann TC, Glasziou PP, Boutron I, et al. [Better Reporting of Interventions: Template for Intervention Description and Replication (TIDieR) Checklist and Guide]. *Gesundheitswesen* 2016;78(3):175-88. doi: 10.1055/s-0041-111066 [published Online First: 20160129]

For peer review only - http://bmjopen.bmj.com/site/about/guidelines.xhtml

35. Graham ID, Logan J. Innovations in knowledge transfer and continuity of care. *Can J Nurs Res* 2004;36(2):89-103.

36. Ørngreen R, Levinsen K. Workshops as a Research Methodology. *Electronic Journal of e-Learning* 2017;15(1):70-81.

37. Braun V, Clarke V. Using thematic analysis in psychology. *Qualitative Research in Psychology* 2006;3(2):77-101. doi: 10.1191/1478088706qp063oa

38. Braun V, Clarke V. Reflecting on reflexive thematic analysis. *Qualitative Research in Sport, Exercise and Health* 2019;11(4):589-97. doi: 10.1080/2159676X.2019.1628806

39. O'Cathain A, Croot L, Duncan E, et al. Guidance on how to develop complex interventions to improve health and healthcare. *BMJ Open* 2019;9(8):e029954. doi: 10.1136/bmjopen-2019-029954 [published Online First: 20190815]

40. Turner KM, Rousseau N, Croot L, et al. Understanding successful development of complex health and healthcare interventions and its drivers from the perspective of developers and wider stakeholders: an international qualitative interview study. *BMJ Open* 2019;9(5):e028756. doi: 10.1136/bmjopen-2018-028756 [published Online First: 20190530]

41. O'Cathain A, Thomas KJ, Drabble SJ, et al. What can qualitative research do for randomised controlled trials? A systematic mapping review. *BMJ Open* 2013;3(6):e002889. doi: 10.1136/bmjopen-2013-002889

42. Moore JB, Maddock JE, Brownson RC. The Role of Dissemination in Promotion and Tenure for Public Health. *J Public Health Manag Pract* 2018;24(1):1-3. doi: 10.1097/phh.0000000000000691

43. Wolfenden L, Mooney K, Gonzalez S, et al. Increased use of knowledge translation strategies is associated with greater research impact on public health policy and practice: an analysis of trials of nutrition, physical activity, sexual health, tobacco, alcohol and substance use interventions. *Health Res Policy Syst* 2022;20(1):15. doi: 10.1186/s12961-022-00817-2 [published Online First: 20220131]

For peer review only

For peer review only - http://bmjopen.bmj.com/site/about/guidelines.xhtml

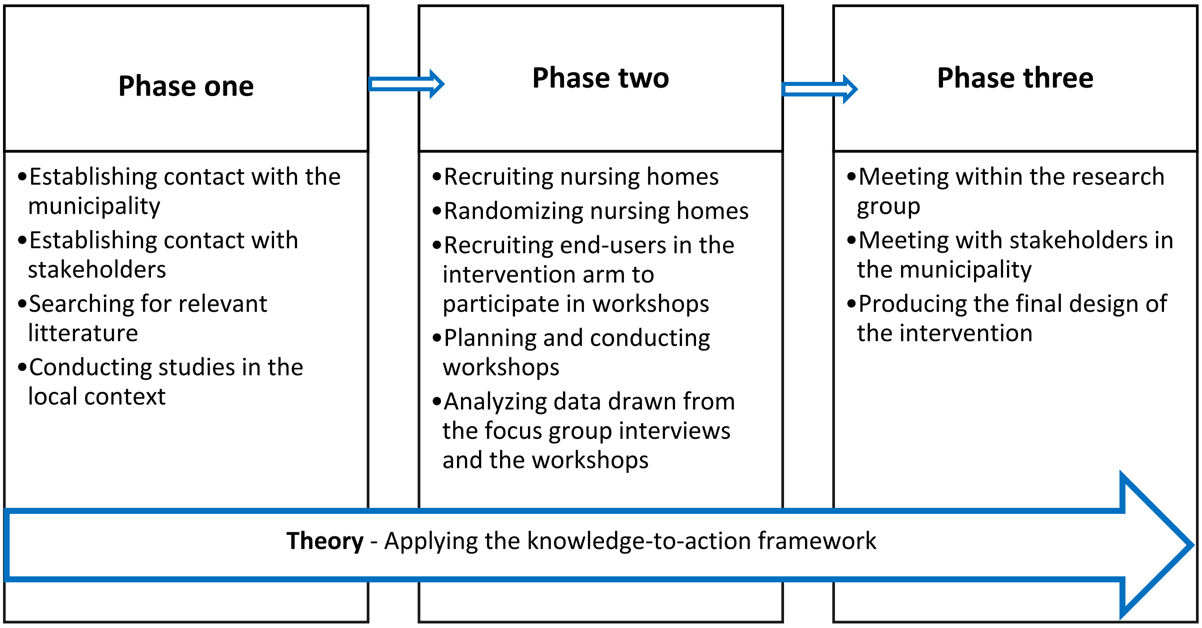

Figure 1. Phases one-three illustrate the process of developing the STAIR OF KNOWLEDGE intervention, which took place between 2019 and 2022. Although the knowledge-to-action (KTA) framework is viewed as a cycle by Graham and colleagues (2006), in this figure, the arrow illustrates the fact that the KTA framework was applied throughout phases one-three of the development process. The KTA framework was applied in an iterative and dynamic way in each phase and is described in detail in the text.

For peer review only

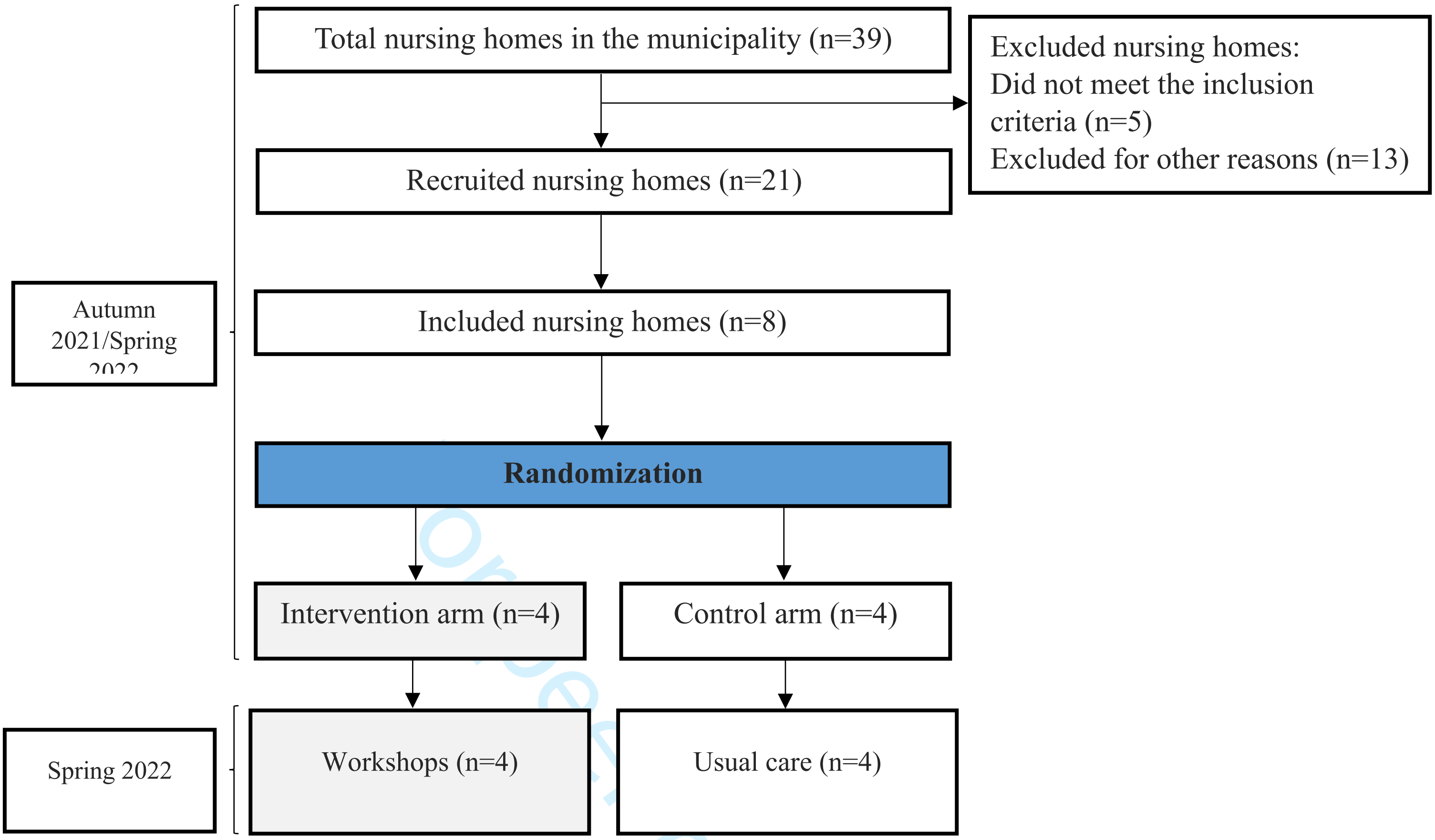

Figure 2. Flowchart of the two-arm pragmatic cluster randomized controlled trial. The flowchart illustrates phase two of the development of the STAIR OF KNOWLEDGE intervention.

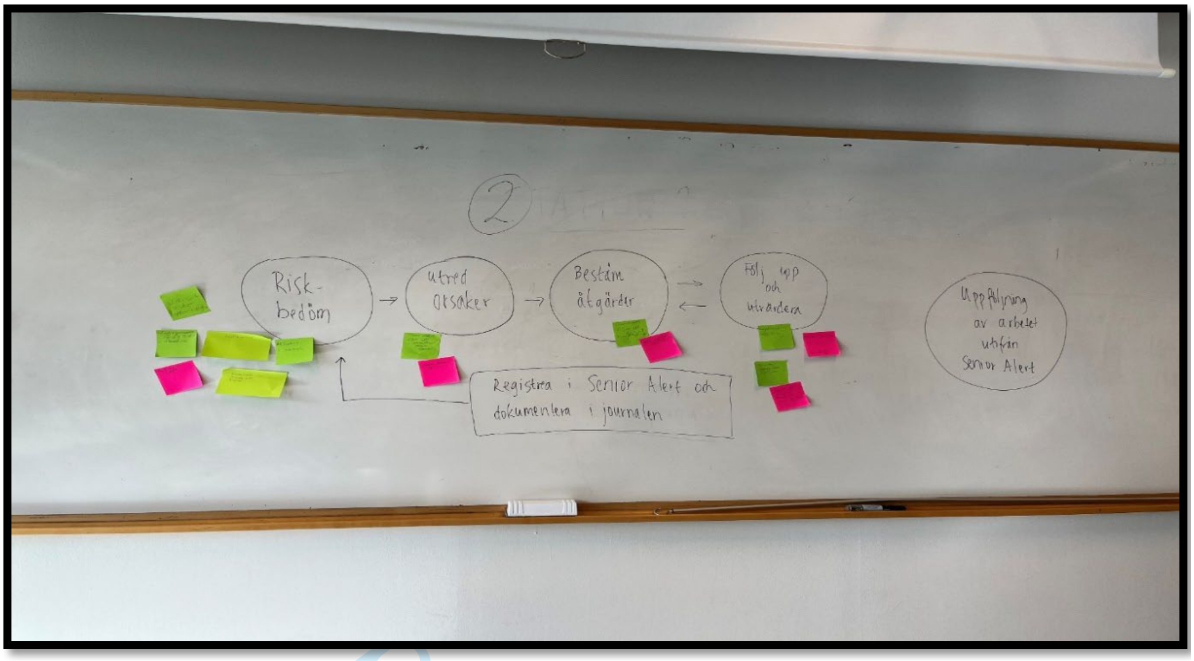

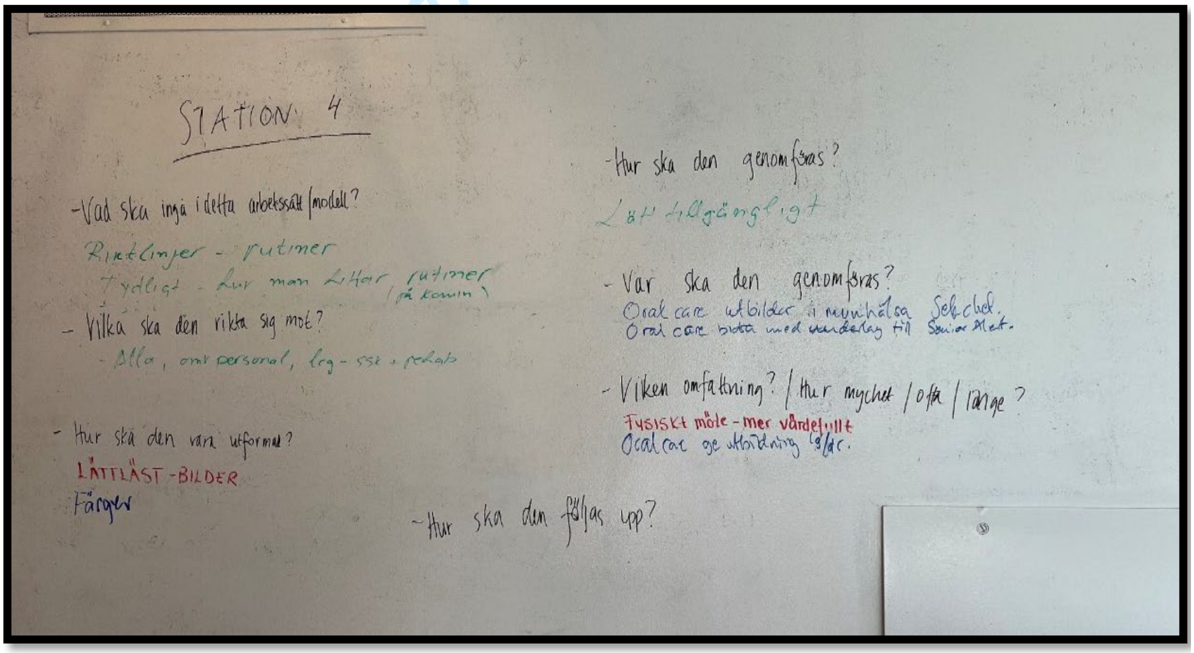

Figure 3. These pictures illustrate the end-users´ participation in stations two and four in one of the four workshops.

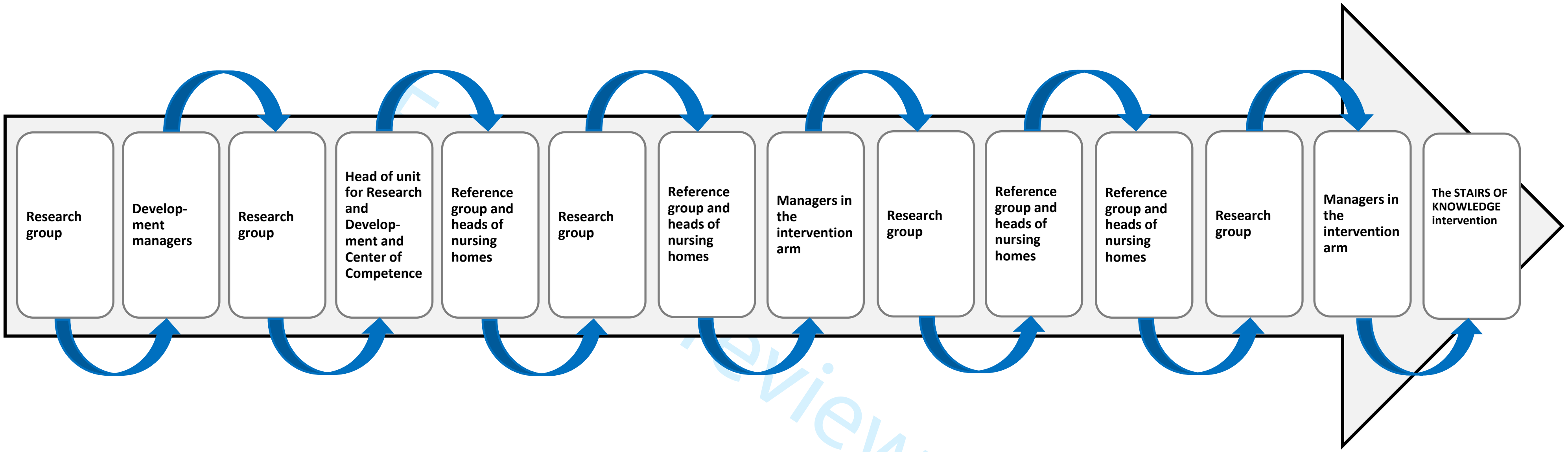

Figure 4. The iterative and dynamic process of designing the final outline of the STAIR OF KNOWLEDGE intervention between April and September 2022, including meetings and discussions with stakeholders. The blue arrows illustrate that adjustments were made following each meeting.

The STAIR OF KNOWLEDGE

The STAIR OF KNOWLEDGE is addressed to all nurse aides, registered nurses and managers who work with older persons in nursing homes. The STAIR OF KNOWLEDGE aims to increase knowledge regarding the preventive care necessary to prevent the risks of falls, pressure ulcers, malnutrition and poor oral health.

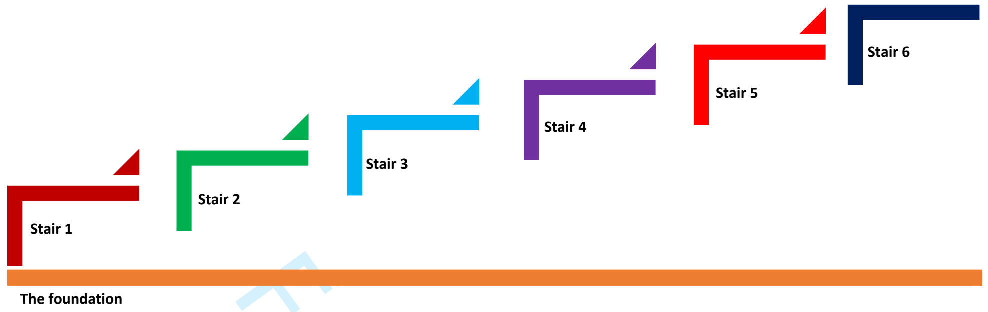

The foundation is mandatory for nurse aides, registered nurses and managers. **Stairs 1-4** and **stair 6** are mandatory for all nurse aides, registered nurses and managers. **Stair 5** is mandatory for individuals who register in Senior Alert. Follow the instructions below.

For your convenience, click the boxes as you progress through the STAIR OF KNOWLEDGE.

Foundation	☐	Stair 4a-d	☐
Stair 1a-d	☐	Stair 5	☐
Stair 2a-d	☐	Stair 6	☐
Stair 3	☐		

The foundation. Local working description of the entire preventive care working process.

- Link to the local working routine.

Stair 1a-d. General information regarding falls, pressure ulcers, malnutrition and poor oral health.

- Links to texts and videos regarding falls, pressure ulcers, malnutrition and poor oral health.

Stair 2a-d. Risk assessment of falls, pressure ulcers, malnutrition and poor oral health.

- Links to texts and videos regarding the risk assessment of falls, pressure ulcers, malnutrition and poor oral health.

Stair 3. Causes of falls, pressure ulcers, malnutrition and poor oral health.

- Link to text regarding the causes of falls, pressure ulcers, malnutrition and poor oral health.

Stair 4a-d. Preventive care interventions for falls, pressure ulcers, malnutrition and poor oral health.

- Links to texts regarding preventive care interventions for falls, pressure ulcers, malnutrition and poor oral health.

Stair 5. Registering in Senior Alert

- Links to texts and videos regarding how to register in Senior Alert.

Stair 6. Inspiration week

- Inspiration week focuses on preventive care intended to prevent the risks of falls, pressure ulcers, malnutrition and poor oral health in an inspiring and motivating way. The inspiration week will be organized by and for employees and managers. The inspiration week is preferably organized twice per year.

Figure 5. Final design of the STAIR OF KNOWLEDGE intervention.

For peer review only

GUIDED – a guideline for reporting for intervention development studies.

Supplementary File 1: Blank Checklist

Item description	Explanation	Page in manuscript where item is located	Other*
1. Report the context for which the intervention was developed.	Understanding the context in which an intervention was developed informs readers about the suitability and transferability of the intervention to the context in which they are considering evaluating, adapting or using the intervention. Context here can include place, organisational and wider socio-political factors that may influence the development and/or delivery of the intervention (15).	Page 5 (Study context)	
2. Report the purpose of the intervention development process.	Clearly describing the purpose of the intervention specifies what it sets out to achieve. The purpose may be informed by research priorities, for example those identified in systematic reviews, evidence gaps set out in practice guidance such as The National Institute for Health and Care Excellence or specific prioritisation exercises such as those undertaken with patients and practitioners through the James Lind Alliance.	Page 3-4 (Introduction) Page 6-10) Development of the intervention)	
3. Report the target population for the intervention development process.	The target population is the population that will potentially benefit from the intervention – this may include patients, clinicians, and/or members of the public. If the target population is clearly described then readers will be able to understand the relevance of the intervention to their own research or practice. Health inequalities, gender and ethnicity are features of the target population that may be relevant to intervention development processes.	Page 6-10 (Development of the intervention) Page 10 (Results)	
4. Report how any published intervention development approach contributed to the development process	Many formal intervention development approaches exist and are used to guide the intervention development process (e.g. 6Squid (16) or The Person Based Approach to Intervention Development (17)). Where a formal intervention development approach is used, it is helpful to describe the process that was followed, including any deviations. More general approaches to intervention development also exist and have been categorised as follows (3):- Target Population-centred intervention development; evidence and theory-based intervention development; partnership intervention development; implementation-based intervention development; efficacy-based intervention development; step or phased-based intervention development; and intervention-specific intervention development (3). These approaches do not always have specific guidance that describe their use. Nevertheless, it is helpful to give a rich description of how any published approach was operationalised	Page 3-4 (Introduction) Page 5 (Study design)	
5. Report how evidence from different sources informed the intervention development process.	Intervention development is often based on published evidence and/or primary data that has been collected to inform the intervention development process. It is useful to describe and reference all forms of evidence and data that have informed the development of the intervention because evidence bases can change rapidly, and to explain the manner in which the evidence and/or data was used. Understanding what evidence was and was not available at the time of intervention development can help readers to assess transferability to their current situation.	Page 6-10 (Development of the intervention)	
6. Report how/if published theory informed the intervention development process.	Reporting whether and how theory informed the intervention development process aids the reader's understanding of the theoretical rationale that underpins the intervention. Though not mentioned in the e-Delphi or consensus meeting, it became increasingly apparent through the development of our guidance that this theory item could relate to either existing published theory or programme theory	Page 3-4 (Introduction) Page 5 (Study design) Page 6 (Theory)	
7. Report any use of components from an existing intervention in the current intervention development process.	Some interventions are developed with components that have been adopted from existing interventions. Clearly identifying components that have been adopted or adapted and acknowledging their original source helps the reader to understand and distinguish between the novel and adopted components of the new intervention.	The intervention is based on exising evidence-based knowledge in Senior Alert. See figure 5.	
8. Report any guiding principles, people or factors that were prioritised when making decisions during the intervention development process.	Reporting any guiding principles that governed the development of the application helps the reader to understand the authors' reasoning behind the decisions that were made. These could include the examples of particular populations who views are being considered when designing the intervention, the modality that is viewed as being most appropriate, design features considered important for the target population, or the potential for the intervention to be scaled up.	Page 6-10 (Development of the intervention)	

1

For peer review only

Duncan E, *et al. BMJ Open* 2020; 10:e033516. doi: 10.1136/bmjopen-2019-033516

Item description	Explanation	Page in manuscript where item is located	Other*
9. Report how stakeholders contributed to the intervention development process.	Potential stakeholders can include patient and community representatives, local and national policy makers, health care providers and those paying for or commissioning health care. Each of these groups may influence the intervention development process in different ways. Specifying how differing groups of stakeholders contributed to the intervention development process helps the reader to understand how stakeholders were involved and the degree of influence they had on the overall process. Further detail on how to integrate stakeholder contributions within intervention reporting are available (19).	Page 6-10 (Development of the intervention)	
10. Report how the intervention changed in content and format from the start of the intervention development process.	Intervention development is frequently an iterative process. The conclusion of the initial phase of intervention development does not necessarily mean that all uncertainties have been addressed. It is helpful to list remaining uncertainties such as the intervention intensity, mode of delivery, materials, procedures, or type of location that the intervention is most suitable for. This can guide other researchers to potential future areas of research and practitioners about uncertainties relevant to their healthcare context.	Page 6 (Analysed the data from the focus group interviews and workshops) + Page 7 (Third phase)	
11. Report any changes to interventions required or likely to be required for subgroups.	Specifying any changes that the intervention development team perceive are required for the intervention to be delivered or tailored to specific sub groups enables readers to understand the applicability of the intervention to their target population or context. These changes could include changes to personnel delivering the intervention, to the content of the intervention, or to the mode of delivery of the intervention.	Page 8-9 (Analysed the data from the focus group interviews and workshops) + Page 9 (Third phase) + Page 10 (Results)	
12. Report important uncertainties at the end of the intervention development process.	Intervention development is frequently an iterative process. The conclusion of the initial phase of intervention development does not necessarily mean that all uncertainties have been addressed. It is helpful to list remaining uncertainties such as the intervention intensity, mode of delivery, materials, procedures, or type of location that the intervention is most suitable for. This can guide other researchers to potential future areas of research and practitioners about uncertainties relevant to their healthcare context.	Page 11-12 (Discussion)	
13. Follow TIDieR guidance when describing the developed intervention.	Interventions have been poorly reported for a number of years. In response to this, internationally recognized guidance has been published to support the high quality reporting of health care? interventions[5]and public health interventions[14]. This guidance should therefore be followed when describing a developed intervention.	The description of the intervention followsTIDieR	
14. Report the intervention development process in an open access format.	Unless reports of intervention development are available people considering using an intervention cannot understand the process that was undertaken and make a judgement about its appropriateness to their context. It also limits cumulative learning about intervention development methodology and observed consequences at later evaluation, translation and implementation stages. Reporting intervention development in an open access (Gold or Green) publishing format increases the accessibility and visibility of intervention development research and makes it more likely to be read and used. Potential platforms for open access publication of intervention development include open access journal publications, freely accessible funder reports or a study web-page that details the intervention development process.	Submitted to the BMJ Open	

*e.g. if item is reported elsewhere, then the location of this information can be stated here.

For peer review only - http://bmjopen.bmj.com/site/about/guidelines.xhtml

Duncan E, *et al. BMJ Open* 2020; 10:e033516. doi: 10.1136/bmjopen-2019-033516

BMJ Open

The STAIR OF KNOWLEDGE – a codesigned intervention to prevent pressure ulcers, malnutrition, poor oral health and falls among older persons in nursing homes: development of a complex intervention

Journal:	*BMJ Open*
Manuscript ID	bmjopen-2023-072453.R1
Article Type:	Original research
Date Submitted by the Author:	04-Apr-2023
Complete List of Authors:	Neziraj, Merita; Malmo University, Department of Care Science, Faculty of Health and Society<br>Axelsson, Malin; Malmo University, Department of Care Science, Faculty of Health and Society<br>Kumlien, Christine ; Malmo University, Department of Care Science, Faculty of Health and Society; Skanes universitetssjukhus Malmo, Department of Cardio-Thoracic and Vascular Surgery, Skane University Hospital, Malmö, Sweden<br>Hellman, Peter; Malmo University, Department of Care Science, Faculty of Health and Society<br>Andersson, Magdalena; Unit of Research and development and competence centre, City of Malmö, Sweden, Health and Social Care, Strategic Development
**Primary Subject Heading**:	Nursing
Secondary Subject Heading:	Nursing
Keywords:	PREVENTIVE MEDICINE, Aging, Nursing Care

SCHOLARONE™
Manuscripts

I, the Submitting Author has the right to grant and does grant on behalf of all authors of the Work (as defined in the below author licence), an exclusive licence and/or a non-exclusive licence for contributions from authors who are: i) UK Crown employees; ii) where BMJ has agreed a CC-BY licence shall apply, and/or iii) in accordance with the terms applicable for US Federal Government officers or employees acting as part of their official duties; on a worldwide, perpetual, irrevocable, royalty-free basis to BMJ Publishing Group Ltd ("BMJ") its licensees and where the relevant Journal is co-owned by BMJ to the co-owners of the Journal, to publish the Work in this journal and any other BMJ products and to exploit all rights, as set out in our licence.

The Submitting Author accepts and understands that any supply made under these terms is made by BMJ to the Submitting Author unless you are acting as an employee on behalf of your employer or a postgraduate student of an affiliated institution which is paying any applicable article publishing charge ("APC") for Open Access articles. Where the Submitting Author wishes to make the Work available on an Open Access basis (and intends to pay the relevant APC), the terms of reuse of such Open Access shall be governed by a Creative Commons licence – details of these licences and which Creative Commons licence will apply to this Work are set out in our licence referred to above.

Other than as permitted in any relevant BMJ Author's Self Archiving Policies, I confirm this Work has not been accepted for publication elsewhere, is not being considered for publication elsewhere and does not duplicate material already published. I confirm all authors consent to publication of this Work and authorise the granting of this licence.

For peer review only

Title

The STAIR OF KNOWLEDGE – a codesigned intervention to prevent pressure ulcers, malnutrition, poor oral health and falls among older persons in nursing homes: development of a complex intervention

Merita Neziraj[1]

Email: merita.neziraj@mau.se

Malin Axelsson[1]

Email: malin.axelsson@mau.se

Christine Kumlien[1, 2]

Email: christine.kumlien@mau.se

Peter Hellman[1]

Email: peter.hellman@mau.se

Magdalena Andersson[3]

Email: magdalena.andersson@malmo.se

[1]*Department of Care Science, Faculty of Health and Society, Malmö University, Sweden*

[2]*Department of Cardio-Thoracic and Vascular Surgery, Skane University Hospital, Malmö, Sweden*

[3]*Department of Health and Social Care, Strategic Development, Unit of Research and development and competence centre, City of Malmö, Sweden*

Corresponding Author: Merita Neziraj. merita.neziraj@mau.se

For peer review only

For peer review only - http://bmjopen.bmj.com/site/about/guidelines.xhtml

Abstract

Objectives: To describe the development of a codesigned complex intervention intended to prevent the risks of pressure ulcers, malnutrition, poor oral health and falls among older persons in nursing homes.

Design: A complex intervention development study. The development of the intervention was conducted in three phases. We established contact with stakeholders in the municipality, updated us of current status of the literature in this area and conducted studies in the local context (1). We codesigned the intervention in workshops together with end users (2). We codesigned the final outline of the intervention in an iterative process with stakeholders (3).

Setting: Nursing homes in the municipality in southern Sweden.

Participants: End users (n=16) in nursing homes (n=4) codesigned the intervention together with the research group in workshops (n=4) in March-April 2022. Additionally, stakeholders (n=17) who were considered to play an important role in developing the intervention participated throughout this process. Data were analysed using reflexive thematic analysis.

Results: Four workshops were conducted with end users (n=16) and thirteen meetings with stakeholders (n=12) were held during the development process. The intervention aims to bridge the evidence-practice gap regarding the preventive care process of the risks of pressure ulcers, malnutrition, poor oral health and falls among older persons in nursing homes. The intervention is aimed at end users, lasts for three weeks and is divided into two parts. First, end users obtain knowledge on their own by following written instructions. Second, they meet, interact and discuss the knowledge acquired during part one.

Conclusion: The intervention is robustly developed and thoroughly described. The study highlights the extensive process that is necessary for developing tailored complex interventions. The description of the entire development process may enhance the replicability of this intervention. The intervention needs to be tested and evaluated in an upcoming feasibility study.

Trial number: Clinical Trial NCT05308862.

For peer review only

For peer review only - http://bmjopen.bmj.com/site/about/guidelines.xhtml

Strengths

- Inspired by the Medical Research Council guidelines for complex intervention, a robust development process was undertaken based on the literature and research conducted in the local context prior to developing the complex intervention.
- A complex intervention was codesigned both with and for nurse aides, registered nurses and managers in workshops. Additionally, key persons working in the municipality were engaged in the development of this tailored intervention.
- To bridge the evidence-practice gap regarding the risks of pressure ulcers, malnutrition, poor oral health and falls among older persons in nursing homes, knowledge translation strategies were applied during the development process in accordance with the action part of the knowledge-to-action framework.
- A thorough description of the entire development process may enhance the replicability of the current intervention.

Limitations

- One limitation of the development process was that this design is time- and resource-consuming. On the other hand, this was necessary to develop a tailored complex intervention that might enhance the likelihood of successful implementation. The transferability of the tailored intervention to other nursing homes might also be a limitation.

For peer review only - http://bmjopen.bmj.com/site/about/guidelines.xhtml

Introduction

There remains an evidence-practice gap in preventing the risks of pressure ulcers, malnutrition, poor oral health and falls among older persons in nursing homes[1 2]. These health risks cause a major burden for older persons[3] and they are costly for the health care system[4]. Since older persons are more vulnerable to these health risks[5] and considering the increasing ageing population globally, particularly with regard to older persons aged 80 years or older[6], evidence-based preventive work is crucial to manage this demographic challenge and, importantly, these health risks among older persons.

In Sweden, there is a national quality register, Senior Alert, providing an individualized, standardized, structured and systematic preventive care work process for older persons 65 years or older who are at risk of pressure ulcers, malnutrition, poor oral health and falls[7]. Senior Alert provides evidence-based knowledge aimed at preventing these health risks to enable a healthy ageing among older persons[8]; in addition, it can increase cost efficiency[9]. However, a lack of knowledge among those working with older persons has been identified as one major challenge regarding to preventive work[2 10]. As a result, these health risks continue to be prevalent[7]. For instance, approximately every third older person living in a nursing home faces at least one of these health risks, and every tenth older person faces all four of these health risks[1]. Additionally, not all older persons who are at risk have planned care interventions[11 12] and there is a mismatch between identified risks and planned and performed care interventions[13 14], thus indicating an evidence-practice gap and consequently, highlighting the urgent need of translating knowledge into practice.

Nevertheless, this is not unique to Sweden or this context; in contrast, health systems worldwide face the shared challenge of translating knowledge into practice[15]. Knowledge translation has been defined as *"a dynamic and iterative process that includes synthesis, dissemination, exchange and ethically sound application of knowledge to improve health care of people in the country, provide more effective health service and products and strengthen the health care system",* p. 165[16]. Ineffective knowledge translation can result in an evidence-practice gap[17] and, worryingly, lead to situations in which patients are denied interventions that have been proven to be beneficial[18], which in turn can result in a reduction in their quality of life[19].

For peer review only - http://bmjopen.bmj.com/site/about/guidelines.xhtml

To bridge this evidence-practice gap, conceptual frameworks are recommended[20]. The knowledge-to-action (KTA) framework is intended to help the parties involved in the process of knowledge translation[18]. The KTA framework is also appropriate when addressing an evidence-practice gap[15] and conducting pragmatic research[18].

As a part of translating knowledge into practice and promoting knowledge use by end users[21], the engagement of both researchers and stakeholders in research is crucial[22]. Engaging stakeholders at an early stage in the development of solutions that can be applied to real world settings is essential according to the Medical Research Council´s (MRC) framework for complex interventions[23]. Complex interventions have multiple components, target multiple groups or levels of an organization and attempt to affect multiple outcomes[23]. Additionally, for complex interventions to be most useful to end users, the local context must be taken into account[24]. Since it is well underpinned that organizational factors hinder preventive work in nursing homes[2 25], considering and understanding the local context and integrating it into the process of intervention development is crucial[26].

Consequently, change in the practices of nursing homes is considered to be complex[27], but if complex interventions are tailored to the local context[28], including the targets of the intervention[23 24] and is directly relevant to them[29], such interventions could be successful.

Aim

The aim of this study was to describe the development of a codesigned complex intervention intended to prevent pressure ulcers, malnutrition, poor oral health and falls among older persons in nursing homes.

Methods

Definitions

Nursing homes were defined based on the definition provided by Neziraj et al. (2021)[1]: older persons receiving municipal health care in residential care homes.

Health care personnel and managers were defined based on the definition provided by Neziraj et al. (2021) as follows[2]:

Nurse aide: a person with a secondary degree in nursing, involves three years of study in high school *or* a person without any formal education in nursing.

For peer review only - http://bmjopen.bmj.com/site/about/guidelines.xhtml

Registered nurse: a person with a bachelor's degree in nursing, which involves three years of study at university.

Manager: a person who is in charge of nurse aides or registered nurses.

End users: nurse aides, registered nurses and managers working in nursing homes.

Stakeholders: key persons working in the municipality who are considered to play an important role in the development and implementation of the intervention.

Study context and setting

In nursing homes, nurse aides are the main providers of care and services and are on duty around the clock. Nurse aides work under the regulations of the Social and Services Act (SFS)[30] but are also delegated tasks according to the Health and Medical Services Act (HSL)[31], usually by registered nurses. Registered nurses guide care in nursing homes and work under the regulations of HSL[31]. In the current setting, a large town located in southern Sweden with 39 nursing homes, one registered nurse (or occasionally more depending on the size of the nursing home) is located in the nursing home during office hours but is also available at any other time. Managers who are in charge of the care and services provided by the nurse aides are located at their respective nursing homes during office hours.

For transparency, the research group (n=5) positions are reported; four of the researchers hold positions as either doctoral students (MN), associated professors (MAX), professors (CK) or senior lectures (PH) at the affiliated university. The last author (MA) is a PhD and hold the position as a research and development coordinator in the municipality where the study was conducted. All the authors are registered nurses, and two of them (MN, MA) specialize in elderly care and have worked in this context previously,

In addition, a reference group was created, which consisted of experts (n=7) drawn from the local context; nurse aide (n=1), managers in charge of nursing homes (n=2), head of managers in charge of registered nurses (n=1), development managers (n=2) and head of the nursing homes in the municipality (n=1).

Study design

The current study was a part of the PROSENIOR program focusing on prevention of pressure ulcers, malnutrition, poor oral health and falls among older persons in nursing homes (PROSENIOR: Prevention of falls, pressure ulcers, malnutrition and poor oral health in older

For peer review only - http://bmjopen.bmj.com/site/about/guidelines.xhtml

persons in Skåne | Malmö University (mau.se)). This part of the PROSENIOR program is designed as a two-arm pragmatic cluster randomized trial where the overall aim is to develop, test and evaluate a codesigned complex intervention to prevent pressure ulcers, malnutrition, poor oral health and falls among older persons living in nursing homes. However, the current study focused on the development of a codesigned complex intervention (hereafter called the intervention). The development of the intervention was conducted in three phases. The phases are described below. The development of the current intervention was conducted in a pragmatic paradigm as it is intended to work in a real-world setting [29]; this process was inspired by the MRC guidelines for complex interventions[24], applied the KTA framework[18] and engaged end users and stakeholders in the process of codesign[32].

We follow the guidance for reporting intervention development studies (GUIDED)[33] when describing the development of the intervention and the template for intervention description and replication (TIDieR) checklist and guide[34] when describing the intervention. We use "development" to refer to the whole process of intervention development and "design" to indicate the intervention content, format and delivery.

Patient and public involvement

Patients or informal caregivers were not involved in the research process. End users codesigned the intervention with the research group in workshops. Stakeholders were also involved in this research; they supported the research group throughout the entire development of the intervention by contributing their valuable knowledge. All engagement is described in detail in the section "Development of the intervention" below.

Development of the intervention

We developed the intervention in three phases and applied the KTA framework in all phases (Figure 1).

Theory

The KTA framework takes implementation strategies into account already in the development phase[18], which promotes and sustains practice change[15]. We applied the KTA framework because it offers a structured and systematic approach to translate knowledge into practice[18]. It comprises two parts: knowledge creation and the action cycle. Since evidence-based knowledge is already available to end users in the quality register Senior Alert, the action

For peer review only - http://bmjopen.bmj.com/site/about/guidelines.xhtml

cycle was applied during the development of the current intervention. The action cycle consists of the following steps: *1. Identify the problem, identify and review selected knowledge, 2. Adapt knowledge to the local context, 3. Assess barriers to knowledge use, 4. Select and tailor implementation strategies, 5. Monitor knowledge use, 6. Evaluate the outcomes* and *7. Sustain knowledge use*[18 35]. Steps 1-4 the action cycle were applied throughout the development process of developing the intervention in an iterative, dynamic and permeable way.

Phase one

During this phase, we established contact with stakeholders in the municipality, updated us the of current status of the literature in this area and conducted studies in the local context.

Establishing contact with stakeholders in the municipality

Initially, we established contact and met with the head of the nursing homes in the municipality. The reference group was created in this phase (described in the paragraph "Study context and setting" above).

Searching for literature and conducting studies in the local context

As a part of step 1 in the KTA framework*, identify the problem, identify and review selected knowledge*, firstly, we updated us of current status of the literature regarding prevention of pressure ulcers, malnutrition, poor oral health and falls and intervention studies in this area. Subsequently, we conducted a cross-sectional study to determine the prevalence of the risks of pressure ulcers, malnutrition, poor oral health and falls in nursing homes in southern Sweden[1].

As a part of steps 2-3 in the KTA framework, *adapt knowledge to the local context and assess barriers to knowledge use,* we conducted focus group interviews (n=5) with end users (n=21) who worked in nursing homes to prevent pressure ulcers, malnutrition, poor oral health and falls[2]. The focus group interviews lasted between 63 and 106 min (mean 83 min). A detailed description of this study and its participants is provided in Neziraj et al[2]. Additionally, we asked the end users included in our previous study, i.e., Neziraj et al[2], how an optimal intervention could be designed to prevent the risks of pressure ulcers, malnutrition, poor oral health and falls among older persons in nursing homes. These particular data were targeted for

For peer review only - http://bmjopen.bmj.com/site/about/guidelines.xhtml

the current study. Hence, these data were not reported in our previous study, but are included, analysed and reported in our current study.

Phase one suggested that individuals working with older persons in nursing homes need increased knowledge concerning how to prevent these health risks. Since existing evidence and knowledge concerning how to prevent these health risks is already contained in Senior Alert, the challenge seems to lie in the evidence-practice gap, which highlights the need for support to be provided to end users with regard to translating evidence into practice[1 2].

Phase two

During this phase, we recruited and randomized nursing homes. Subsequently, we invited end users in the intervention arm to participate in workshops, and planned and conducted the workshops. We also analysed the specific data regarding intervention design drawn from the focus group interviews (see the previous paragraph on phase one for clarification) and the workshops.

Recruiting and randomizing nursing homes

For the current study's purpose, randomization was only conducted to invite end users in nursing homes allocated to the intervention arm to codesign an intervention together with the research group in workshops (Figure 2). The nursing home is the cluster and the unit of allocation. Due to the nature of the design, the cluster randomization of nursing homes was unblinded. The randomization procedure will be further reported in detail in an upcoming feasibility study.

Inclusion criteria for the study were nursing homes working with and registered in the quality register Senior Alert. We recruited eligible nursing homes (n=21) to participate in the study via digital meetings. In total, eight nursing homes agreed to participate and were cluster randomized using a computerized program to either the intervention (n=4) or control arm (n=4). Subsequently, we invited end users (n=118) working in nursing homes in the intervention arm to participate in workshops intended to develop a tailored intervention together with the research group; the invitations were extended both via a digital information video and in written form. The remaining end users (n=184) working in the nursing homes who were allocated to the control arm continued with their usual care routine.

Conducting workshops

For peer review only - http://bmjopen.bmj.com/site/about/guidelines.xhtml

As a part of steps 2-4 in the KTA framework, *adapt knowledge to the local context, assess barriers to knowledge use* and *select and tailor implementation strategies*, we conducted workshops with end users. In total, four workshops were conducted, which featured two nurse aides, one registered nurse and one manager in each workshop; the workshops were conducted over the course of four weeks (March-April 2022). The workshops were kept small to offer the end users the possibility of exhibiting activity and creativity[36]. The first author (MN) led the workshops together with one of the coauthors (all coauthors participated in one workshop each). The workshops were intended to serve as a place in which participants could learn together and discuss the design of the intervention in four different stations (Table 1). The end users engaged in active discussion and wrote creative ideas and suggestions on the walls and the board in a lecture hall designed for the purpose of encouraging creative pedagogy (Figure 3). In the first station, the end users were asked to discuss the risks of pressure ulcers, malnutrition, poor oral health and falls and the care interventions that should be applied. In the second station, they were asked to discuss and identify barriers and facilitators they had encountered in their own work regarding the preventive care process stipulated by Senior Alert (identify a risk, assess causes and plan, undertake and evaluate care intervention). Barriers were written down on pink post-it notes, while facilitators were written down on green post-it notes. These post-it notes were subsequently placed at the appropriate location on the board with regard to the predawn preventive care process. The focus of the discussions at station three was on the end users´ needs and the support they needed throughout the preventive care process. In the fourth station, they were asked to discuss the core components of the intervention, how to provide follow-ups and implementation strategies. After completing each workshop, MN photographed and briefly summarized the written data from each station. This summary was used if the end users in the subsequent workshop reached an impasse and/or discussed and wrote similar suggestions and ideas to those proposed by the end users in the previous workshop. Each workshop lasted for three hours, and the discussions were audio recorded to support the written data collection during the analysis.

For peer review only - http://bmjopen.bmj.com/site/about/guidelines.xhtml

Table 1. Workshop content (n=4).

Workshop	Station	Content	Examples of questions to discuss
Workshop 1-4	Station 1	Case regarding an older person at risk of pressure ulcers, malnutrition, poor oral health and falls living in a nursing home	• What would you have done in this case regarding these four risks?<br>• Are there any good examples? What can you learn from good examples?<br>• What additional knowledge do you need regarding these four risks in order to produce a risk assessment and provide adequate care interventions?
	Station 2	Senior Alert´s care process	• Place green/pink post-it notes on the care process regarding what works/what can be improved in your own work and workplace.
	Station 3	End users needs´ and the support they need regarding preventive work	• What do you need in your preventive work?<br>• Why is this important, and what is most important (rank 1-3)?<br>• Who needs help in the context of preventive work?<br>• Who should be involved and in what way?<br>• What is necessary for it to be feasible?<br>• How can you work better/smarter?<br>• How can you work in a more sustainable way?
	Station 4	Core components of the intervention	• What should be included in the intervention?<br>• Who should it target?<br>• How should it be designed?<br>• How much/often/for how long should the intervention take place?<br>• How should it be followed up?<br>• Where should it be implemented?<br>• How should it be implemented?

For peer review only

Analysing the data from the focus group interviews and the workshops

The analysis was guided by the six phases of reflexive thematic analysis described by Braun and Clarke[37 38]: *1. Familiarizing with the data, 2. Coding, 3. Generating initial themes, 4. Reviewing the identified themes, 5. Defining and naming the themes* and *6. Producing the report.* Thematic analysis was chosen because it facilitates a flexible analysis process but simultaneously provides researchers with the core skills they need to conduct the analysis.

To familiarize ourselves with the data, MN and MA read the transcripts from the focus group interviews, including the data specifically collected for the current study, and the written data collected from the workshops. In addition, MN listened to all the audio-recorded discussions from the workshops meticulously. During the process of reading the data, MN and MA reflected on and generated initial codes. Subsequently, MN and MA met and discussed these initial codes (1). Thereafter, MN and MA separately engaged in a process of identifying and coding entities of interest in relation to the design of the intervention, giving equal attention to all the data (2). The initial codes were then sorted into their core components in relation to the design of the intervention (3). Next, the core components were reviewed by MN to determine whether any relevant data regarding the design of the intervention had been missed (4). Subsequently, MN designed an outline of the intervention. This outline contained the intervention's proposed design, including its content, format, plan for delivery and duration. In the following step of the analysis, the entire research group met and discussed the design of the outline of the intervention. During this step, MN continuously revised the outline of the intervention following discussions within the research group (5). Then, the outline of the intervention was redesigned by MN. The redesigned outline of the intervention was then presented to the research group before it was presented to the stakeholders. The process of producing the final design of the intervention is described in phase three below (6).

Phase three

As part of steps 2-4 in the KTA framework, *adapt knowledge to the local context, assess barriers to knowledge use* and *select and tailor implementation strategies,* MN and MA met regularly with stakeholders in structured meetings to present and discuss the outline of the intervention. MN documented all the meetings. MA works within the municipality and thus facilitated contact with stakeholders who were considered to play an important role in this part of designing the intervention. Since this part of the process was dynamic and iterative and because all relevant uncertainties had not been addressed in the redesigned outline of the

For peer review only - http://bmjopen.bmj.com/site/about/guidelines.xhtml

intervention, it was helpful to meet stakeholders for the purpose of identifying and addressing the remaining uncertainties regarding the content, format, delivery and duration of the intervention. This part of the process was time-consuming and required a back-and-forth process involving meetings and discussions between MN and MA, within the entire research group and with the stakeholders. Next, the redesigned outline of the intervention was adjusted by MN in accordance with the results of these meetings and discussions (Figure 4). Finally, MN investigated whether any data from the focus group interviews and the workshops had been missed, since these data were intended to serve as the foundation for designing the final outline of the intervention. The final design of the intervention, the STAIR OF KNOWLEDGE (Figure 5), is described below.

Results

A majority of the end users (n=16) in workshops (n=4) were women (n=13), between the ages of 28-63 years (mean 53), and had worked for 3-41 years (mean 18). The meetings (n=13) with stakeholders (n=12) lasted between 60-180 min (mean 134 min).

The final design of the intervention

The final design of the intervention was described in line with the TIDieR checklist[34] (Supplementary file).

The STAIR OF KNOWLEDGE consists of *the foundation and stairs 1-6,* lasts for three weeks and is divided into two parts. Part one, including *the foundation and stairs 1-5*, takes place throughout the entire intervention period (weeks 1-3) and is delivered digitally to end users in the nursing homes via their workplace email addresses. Part two includes *stair 6* and takes place during the last week of the intervention period (week 3) in the nursing homes in question (Figure 5).

The content of the intervention

Part one of the intervention: the foundation and stairs 1-5

End users emphasized uncertainties of different professionals' responsibilities regarding the preventive work. For instance, they highlighted that it is highly relevant for respective professional to know "who does what" regarding the preventive working. Hence, *the foundation* is intended to facilitate for different professionals regarding responsibilities for respective profession and working routine in the local context. *The foundation* provide end

For peer review only - http://bmjopen.bmj.com/site/about/guidelines.xhtml

users with knowledge and awareness of how to work preventively in the context of an existing local working routine and is intended to represent “the ground to stand on”.

Furthermore, end users expressed a need of increased knowledge regarding the health risks and the entire preventive working process. They stressed the importance of basic knowledge when working with older persons in nursing homes. According to end users, not all of them has basic knowledge in how to prevent these health risks among older persons. This was particularly common among temporary workers. To meet their need, *stairs 1-4* provide the end users with general knowledge about risks of pressure ulcers, malnutrition, poor oral health and falls according to the care process suggested by the quality register Senior Alert *(stair 1),* risk assessment instruments *(stair 2),* the underlying causes of these risks *(stair 3)* and preventive care interventions (*stair 4)*. *Stairs 1-5* provide end users with website links that allow them to both read texts and watch videos. *Stairs 1-4* are mandatory for all professionals. *Stair 5* provides end users with knowledge of how to register in the quality register Senior Alert and is mandatory only for users who have access to and the responsibility to register in the quality register Senior Alert.

Part two of the intervention: stair 6

Although it was necessary for end users to increase their knowledge on their own regarding the preventive work, they particularly highlighted the importance of physical meetings. This was also stressed as important by stakeholders and was considered as a complement to the first part of the intervention. Therefore, in part two, *stair 6*, a facilitator (MN) meets with end users to interact with them and discuss the knowledge acquired during part one. The meetings will be structured including discussions based on different cases related to pressure ulcers, malnutrition, poor oral health and falls. End users will also perform risk assessments, identify the underlying causes and plan accurate care interventions based on these cases. Additionally, end users will identify environmental risk factors related to the risks of pressure ulcers, malnutrition, poor oral health and falls in their own workplace. They will also discuss and generate ideas how to follow up on the preventive care process on an organizational level. This part of the intervention is intended to inspire end users to prevent pressure ulcers, malnutrition, poor oral health and falls among older persons in nursing homes.

The format of the intervention

Part one of the intervention: the foundation and stairs 1-5

For peer review only - http://bmjopen.bmj.com/site/about/guidelines.xhtml

From end users' perspective, it was important with a clear format. They expressed a need of a structured, readable and colourful working "manual". Hence, the format of the intervention is designed as colourful stair with the intention to visualize the entire preventive working process. To enhance the structure, end users are provided with written instructions in respective stair. Furthermore, stakeholder emphasized the need of a "self-check box" for end users when completing the foundation and stairs in the intervention. Stakeholder believed that this could increase participation and involvement among end users. Since both end users and stakeholders stressed that there are many end users that do not have the Swedish language as their native language, the language is adjusted to suit the local context. Furthermore, end users expressed that the format of the intervention should consider different ways of learning. This was also highlighted as important by stakeholders. Hence, the format consist of both reading texts and watching videos. Moreover, end users and stakeholders emphasized that a digital intervention could be a sustainable solution.

Part two of the intervention: stair 6

End users and stakeholder were in agreement that it is necessary to meet and discuss. Therefore, in part two of the intervention, end users meet in their respective nursing home. Also, the format of this part of the intervention was designed as an inspiration to raise awareness of the preventive work among end users.

The delivery of the intervention

Part one of the intervention: the foundation and stairs 1-5

The intervention will be delivered via email to managers in nursing homes. Subsequently, respective manager will forward the intervention via workplace email addresses to nurse aides and registered nurses. The end users highlighted that some learn better individually while others learn better in group. Therefore, they are permitted to choose if they want to read texts and watch videos individually and/or in group. *The foundation and stairs 1-5* is anticipated to take approximately 10 minutes, 60 minutes, 20 minutes, 10 minutes, 30 minutes and 60 minutes respectively for end users to complete. End users can choose to complete this part of intervention at once or divide it during working hours.

Part two of the intervention: stair 6

For peer review only - http://bmjopen.bmj.com/site/about/guidelines.xhtml

Part two of the intervention will be delivered by a facilitator (MN) who will moderate sessions lasting approximately 30 minutes each, Monday-Friday, in the nursing homes in question. If end users participate in all the sessions during this week, the planned amount of time is two and half hours for each end user.

Discussion

The current codesigned complex intervention, the STAIR OF KNOWLEDGE, was developed together with end users in workshops in an active and creative way. Stakeholders were also engaged in an iterative and dynamic way throughout the development of the intervention, as an important part of undertaking implementation strategies already in the development phase[39]. As recommended by the MRC framework[24], we meticulously considered the relationship between the intervention and its context when developing the intervention. Furthermore, we followed the strategies for knowledge translation included in the KTA framework[18]. Hence, the strengths exhibited by the development of this complex intervention lie in the fact that it was developed both together with and for end users and engaged stakeholders who are considered to play an important role in the development and implementation process. The current intervention is intended to work in a real-world setting and aims to bridge the evidence-practice gap regarding the process of preventing the risks of pressure ulcers, malnutrition, poor oral health and falls; ultimately, this intervention may reduce these risks among older persons in nursing homes.

When developing new intervention, the value of the used design process cannot be understated[40]. In fact, engagement of end users in a creative environment have been linked to more robust research and development efforts, which in turn may drive more successful interventions outcome[40]. Hence, the benefits of codesign are potentially substantial[41]. For instance, engaging end users and stakeholders as design partners to the research group could ensure that the intervention exhibits a better fit to their needs[32]. Engaging end users and stakeholders early enables their experiences and requirements to be taken into account at the start rather than a situation in which the researchers presume to know what is required[39]. In the current development process, although end users´ and stakeholders´ engagement ranged in intensity from relatively passive to highly active, their engagement pervaded the entire development process, and important decisions regarding the intervention design were made by considering their input. Furthermore, because we engaged end users and stakeholders, the current intervention was based on their own experiences regarding the evidence and

For peer review only - http://bmjopen.bmj.com/site/about/guidelines.xhtml

knowledge that are necessary throughout the entire process of preventing the risks of pressure ulcers, malnutrition, poor oral health and falls. Engaging end users and stakeholders during the developing process[42] was also important in light of the local context since this enabled us to identify facilitators and barriers in the environment in which the intervention will eventually be implemented[26].

A recent scoping review investigating education interventions for health professionals on fall prevention in health care settings[43], highlighted that health professional education to prevent fall is important. Nevertheless, the scoping review concluded that there are no uniform education design principles utilized to date[43]. Another review found that it was uncertain whether education delivered in different format such as didactic or video-based format makes a difference to health professionals´ knowledge of pressure ulcers prevention. However, education format in the current developed intervention was designed to fit end users' needs and suit the local context, which may have benefits for the outcome.

Considering and understanding the local context is also crucial when addressing an evidence-practice gap[24]. In this case, knowledge concerning the process of preventing the risks of pressure ulcers, malnutrition, poor oral health and falls is already contained in the quality register Senior Alert, but this evidence has not been fully translated into practice. Thus, we focused on translating the existing knowledge contained in Senior Alert into practice. However, if this knowledge is to be implemented effectively[44], it is crucial to employ a conceptual framework[20]. Therefore, we chose the KTA framework because it provided us with knowledge translation strategies to reduce the evidence-practice gap[18], and it was suitable since the quality register Senior Alert is already in use. Furthermore, adapting knowledge to the local context and assessing barriers to knowledge use may enable the research to have a greater impact[45], which could in turn reduce the evidence-practice gap.

Successful intervention development is characterized as rigorous and scientific and leads to an intervention that can be implemented in a real-world setting[33]. The robust research process used to develop the STAIR OF KNOWLEDGE intervention incorporates existing evidence, the views of end users and stakeholders[41], the local context and knowledge translation strategies. Consequently, the use of knowledge translation strategies and the engagement of end users who are embedded in the local context in the development of a tailored complex intervention both for and with them could contribute to increased knowledge and awareness of the entire process of preventive care. This may, in turn, reduce the evidence-practice gap

For peer review only - http://bmjopen.bmj.com/site/about/guidelines.xhtml

among end users and, importantly, reduce the risk of pressure ulcers, malnutrition, poor oral health and falls among older persons in nursing homes. Furthermore, the engagement of stakeholders already in the development process is likely to facilitate the implementation of the current intervention.

Limitations

Although the development of this complex intervention has been completed, it is important to acknowledge the limitations of the development process. First, only four clusters were included in the development process. Nevertheless, since this part of the trial focused on the development of an intervention rather than its evaluation and because the clusters were recruited pragmatically, the clusters included in the trial could be considered sufficient. Second, although all end users in the intervention arm (n=118) were invited to participate in workshops, only 16 participated. However, different professionals participated in the workshops, and the discussions were energetic, active and creative. Third, although this design is creative and can generate new ideas, it is time- and rescore-consuming for all parties involved. It requires end users and stakeholders to set aside time and expend extra effort in their daily work. For researchers, this process requires careful planning to enable them to coordinate, meet with many different persons repeatedly and be responsive to all parties involved. However, although this design required the expenditure of time and resources, the engagement of end users, stakeholders and researchers is meaningful and necessary to develop successful interventions; ultimately, this design might have an impact on to prevent the risks of pressure ulcers, malnutrition, poor oral health and falls among older persons in nursing homes. Furthermore, the current intervention might offer value when used by others and could likely be adjusted to and tested in similar contexts.

Conclusion

The current codesign complex intervention, the STAIR OF KNOWLEDGE, which aims to prevent the risks of pressure ulcers, malnutrition, poor oral health and falls among older persons in nursing homes, is robustly developed and thoroughly described. A careful description of the entire development process and the intervention itself can enhance the replicability of the current intervention. This article highlights the extensive process that is necessary for the development of tailored complex interventions. Finally, this codesigned complex intervention might result in more evidence-based practice concerning the entire

For peer review only - http://bmjopen.bmj.com/site/about/guidelines.xhtml

process of preventing the risks of pressure ulcers, malnutrition, poor oral health and falls and, importantly, reduce these health risks among older persons in nursing homes. However, uncertainties regarding the intervention itself remain. Thus, the STAIR OF KNOWLEDGE must be tested and evaluated in an upcoming feasibility study before we continue to the stage of conducting a full trial evaluation.

Ethical considerations

This trial was approved by the Swedish Ethical Review Authority (DNR 2019-06414). In addition, written approval was requested and granted by the head of the department of elderly care homes in the municipality in which this trial was conducted. All end users working in eligible nursing homes were invited to participate in the workshops. Moreover, end users had the right to withdraw from participation at any stage without providing reasons and bearing any consequences. Participation in the workshops was based on written consent. The results of this trial may be considered to contribute to scientific value on good ethical grounds, and the benefits of participating in the trial outweigh the corresponding risks.

Funding statement

This research received finical support with regard to conducting the workshops from the Derbrings and Stöltens research and development foundation. The funders had no role in the research manuscript's design, conduct, analysis, interpretation or drafting.

CRediT authorship contribution statement

Merita Neziraj: Conceptualization, Methodology, Investigation, Writing – original draft, Writing – review & editing, Validation, Formal analysis, Visualization. **Malin Axelsson:** Conceptualization, Methodology, Investigation, Writing – review & editing, Validation, Supervision. **Christine Kumlien:** Conceptualization, Methodology, Investigation, Writing – review & editing, Validation, Supervision. **Peter Hellman:** Conceptualization, Methodology, Investigation, Writing – review & editing, Validation, Supervision. **Magdalena Andersson:** Conceptualization, Methodology, Investigation, Writing – review & editing, Validation, Formal analysis, Supervision. All the authors read and approved the final version of the manuscript.

Competing interests

The authors have no conflicts of interest.

For peer review only - http://bmjopen.bmj.com/site/about/guidelines.xhtml

Participant consent

All participation in the workshops was based on written consent.

Acknowledgements

We thank the nurse aides, registered nurses and managers who participated in the development of the intervention. We also thank the stakeholders who participated during this process. Their enthusiastic participation was helpful in developing the STAIR OF KNOWLEDGE intervention. The Derbring and Stölten foundation is acknowledged for its financial support.

Data sharing statement

The data that support the development of the STAIR OF KNOWLEDGE intervention are not publicly available to ensure confidentiality. All data relevant to the development are included in the article. All figures and tables included in this article are original.

For peer review only

For peer review only - http://bmjopen.bmj.com/site/about/guidelines.xhtml

References

1. Neziraj M, Hellman P, Kumlien C, et al. Prevalence of risk for pressure ulcers, malnutrition, poor oral health and falls: a register study among older persons receiving municipal health care in southern Sweden. *BMC Geriatrics* 2021;21(1) doi: 10.1186/s12877-021-02205-x
2. Merita N, Magdalena A, Peter H, et al. Prevention of pressure ulcers, malnutrition, poor oral health and falls in nursing homes: A focus group study with nurse aides, registered nurses and managers. *International Journal of Nursing Studies Advances* 2021;3(100056-) doi: 10.1016/j.ijnsa.2021.100056
3. Gorecki C, Brown JM, Nelson EA, et al. Impact of pressure ulcers on quality of life in older patients: a systematic review. *Journal of the American Geriatrics Society* 2009;57(7):1175-83.
4. Marjolein van der V, Juanita AH, Geraerds AJLM, et al. Health care costs of injury in the older population: a prospective multicentre cohort study in the Netherlands. *BMC Geriatrics* 2020;20(1):1-11. doi: 10.1186/s12877-020-01825-z
5. Bravell ME, Westerlind B, Midlov P, et al. How to assess frailty and the need for care? Report from the Study of Health and Drugs in the Elderly (SHADES) in community dwellings in Sweden. *ARCHIVES OF GERONTOLOGY AND GERIATRICS* 2011;53(1):40-45. doi: 10.1016/j.archger.2010.06.011
6. WHO. Ageing well must be a global priority 2014 [accessed 10-23 2020.
7. Trinks A, Hägglin C, Dennis N, et al. The impact of a national quality register in the analysis of risks and adverse events among older adults in nursing homes and hospital wards: a Swedish senior Alertsurvey. *Safety in Health* 2018;4(10) doi: 10.1186/s40886-018-0077-x
8. Edvinsson J, Rahm M, Trinks A, et al. Senior alert: a quality registry to support a standardized, structured, and systematic preventive care process for older adults. *Quality management in health care* 2015;24(2):96-101.
9. Rubenstein LZ, Harker JO, Salvà A, et al. Screening for undernutrition in geriatric practice: developing the short-form mini-nutritional assessment (MNA-SF). *The journals of gerontology Series A, Biological sciences and medical sciences* 2001;56(6):M366-M72.
10. Dalvand S, Ebadi A, Ghanei Gheshlagh R. Nurses' knowledge on pressure injury prevention: a systematic review and meta-analysis based on the Pressure Ulcer Knowledge Assessment Tool. *Clinical, Cosmetic and Investigational Dermatology* 2018;ume 11:613-20.
11. Annelie KGP, Rose-Marie Johansson-Pajala Ph D, Marina Arkkukangas Ph D, et al. Preventing Falls and Malnutrition among Older Adults in Municipal Residential Care in Sweden: A Registry Study. *SAGE Open Nursing* 2021;7 doi: 10.1177/23779608211026161
12. Backlund A, Holmbeck O, Kumlien C, et al. A registry study of nursing assessments, interventions and evaluations according to nutrition for persons living in municipal residential care homes. *Nursing open* 2018;5(3) doi: 10.1002/nop2.144
13. Witt S, Englander E, Kumlien C, et al. Mismatch between risk factors and preventive interventions? A register study of fall prevention among older people in one Swedish county. *International journal of older people nursing* 2018;13(4):e12209.
14. Hopewell S, Adedire O, Copsey BJ, et al. Multifactorial and multiple component interventions for preventing falls in older people living in the community. *Cochrane Database of Systematic Reviews* 2018(7) doi: 10.1002/14651858.CD012221.pub2
15. Field B, Booth A, Ilott I, et al. Using the Knowledge to Action Framework in practice: a citation analysis and systematic review. *Implement Sci* 2014;9:172. doi: 10.1186/s13012-014-0172-2 [published Online First: 2014/11/25]
16. Straus SE, Tetroe J, Graham I. Defining knowledge translation. *Cmaj* 2009;181(3-4):165-8. doi: 10.1503/cmaj.081229 [published Online First: 20090720]
17. Brownson RC, Eyler AA, Harris JK, et al. Getting the Word Out: New Approaches for Disseminating Public Health Science. *J Public Health Manag Pract* 2018;24(2):102-11. doi: 10.1097/phh.0000000000000673

For peer review only - http://bmjopen.bmj.com/site/about/guidelines.xhtml

18. Graham ID, Logan J, Harrison MB, et al. Lost in knowledge translation: time for a map? *J Contin Educ Health Prof* 2006;26(1):13-24. doi: 10.1002/chp.47 [published Online First: 2006/03/25]
19. Davis D, Evans M, Jadad A, et al. The case for knowledge translation: shortening the journey from evidence to effect. *Bmj* 2003;327(7405):33-5. doi: 10.1136/bmj.327.7405.33
20. Bowen S, Graham ID. Backwards design or looking sideways? knowledge translation in the real world Comment on "A call for a backward design to knowledge translation". *Int J Health Policy Manag* 2015;4(8):545-7. doi: 10.15171/ijhpm.2015.71 [published Online First: 20150324]
21. Eljiz K, Greenfield D, Hogden A, et al. Improving knowledge translation for increased engagement and impact in healthcare. *BMJ Open Qual* 2020;9(3) doi: 10.1136/bmjoq-2020-000983
22. Graham ID, Kothari A, McCutcheon C. Moving knowledge into action for more effective practice, programmes and policy: protocol for a research programme on integrated knowledge translation. *Implement Sci* 2018;13(1):22. doi: 10.1186/s13012-017-0700-y [published Online First: 20180202]
23. Craig P, Dieppe P, Macintyre S, et al. Developing and evaluating complex interventions: the new Medical Research Council guidance. *Bmj* 2008;337:a1655. doi: 10.1136/bmj.a1655 [published Online First: 2008/10/01]
24. Skivington K, Matthews L, Simpson SA, et al. A new framework for developing and evaluating complex interventions: update of Medical Research Council guidance. *BMJ-BRITISH MEDICAL JOURNAL* 2021;374:n2061. doi: 10.1136/bmj.n2061
25. Lannering C, Ernsth-Bravell M, Johansson L. Prevention of falls, malnutrition and pressure ulcers among older persons: nursing staff's experiences of a structured preventive care process. *Health & Social Care in the Community* 2017;25(3):1011-20. doi: 10.1111/hsc.12400
26. Beran D, Lazo-Porras M, Cardenas MK, et al. Moving from formative research to co-creation of interventions: insights from a community health system project in Mozambique, Nepal and Peru. *BMJ Global Health* 2018;3(6):e001183. doi: 10.1136/bmjgh-2018-001183
27. Rantz MJ, Flesner MK, Zwygart-Stauffacher M. Improving care in nursing homes using quality measures/indicators and complexity science. *J Nurs Care Qual* 2010;25(1):5-12. doi: 10.1097/NCQ.0b013e3181c12b0f [published Online First: 2009/12/17]
28. Per N, Susanne B. Context matters in implementation science: a scoping review of determinant frameworks that describe contextual determinants for implementation outcomes. *BMC Health Services Research* 2019;19(1):1-21. doi: 10.1186/s12913-019-4015-3
29. Zwarenstein M, Treweek S, Gagnier JJ, et al. Improving the reporting of pragmatic trials: an extension of the CONSORT statement. *BMJ* 2008;337:a2390. doi: 10.1136/bmj.a2390
30. Geurden B. Screening for Malnutrition in Healthcare. Universiteit Antwerpen (Belgium), 2014.
31. Lauque S, Arnaud-Battandier F, Mansourian R, et al. Protein-energy oral supplementation in malnourished nursing-home residents. A controlled trial. *Age & Ageing* 2000;29(1):51-56.
32. Slattery P, Saeri AK, Bragge P. Research co-design in health: a rapid overview of reviews. *Health Res Policy Syst* 2020;18(1):17. doi: 10.1186/s12961-020-0528-9 [published Online First: 20200211]
33. Duncan E, O'Cathain A, Rousseau N, et al. Guidance for reporting intervention development studies in health research (GUIDED): an evidence-based consensus study. *BMJ Open* 2020;10(4):e033516. doi: 10.1136/bmjopen-2019-033516 [published Online First: 20200408]
34. Hoffmann TC, Glasziou PP, Boutron I, et al. [Better Reporting of Interventions: Template for Intervention Description and Replication (TIDieR) Checklist and Guide]. *Gesundheitswesen* 2016;78(3):175-88. doi: 10.1055/s-0041-111066 [published Online First: 20160129]
35. Graham ID, Logan J. Innovations in knowledge transfer and continuity of care. *Can J Nurs Res* 2004;36(2):89-103.
36. Ørngreen R, Levinsen K. Workshops as a Research Methodology. *Electronic Journal of e-Learning* 2017;15(1):70-81.

For peer review only

For peer review only - http://bmjopen.bmj.com/site/about/guidelines.xhtml

37. Braun V, Clarke V. Using thematic analysis in psychology. *Qualitative Research in Psychology* 2006;3(2):77-101. doi: 10.1191/1478088706qp063oa
38. Braun V, Clarke V. Reflecting on reflexive thematic analysis. *Qualitative Research in Sport, Exercise and Health* 2019;11(4):589-97. doi: 10.1080/2159676X.2019.1628806
39. O'Cathain A, Croot L, Duncan E, et al. Guidance on how to develop complex interventions to improve health and healthcare. *BMJ Open* 2019;9(8):e029954. doi: 10.1136/bmjopen-2019-029954 [published Online First: 20190815]
40. Sanders EN, Stappers P. Co-creation and the new landscapes of design. *CoDesign* 2008;4(1):5-18. doi: 10.1080/15710880701875068
41. Turner KM, Rousseau N, Croot L, et al. Understanding successful development of complex health and healthcare interventions and its drivers from the perspective of developers and wider stakeholders: an international qualitative interview study. *BMJ Open* 2019;9(5):e028756. doi: 10.1136/bmjopen-2018-028756 [published Online First: 20190530]
42. O'Cathain A, Thomas KJ, Drabble SJ, et al. What can qualitative research do for randomised controlled trials? A systematic mapping review. *BMJ Open* 2013;3(6):e002889. doi: 10.1136/bmjopen-2013-002889
43. Shaw L, Kiegaldie D, Farlie MK. Education interventions for health professionals on falls prevention in health care settings: a 10-year scoping review. *BMC Geriatr* 2020;20(1):460. doi: 10.1186/s12877-020-01819-x [published Online First: 20201109]
44. Moore JB, Maddock JE, Brownson RC. The Role of Dissemination in Promotion and Tenure for Public Health. *J Public Health Manag Pract* 2018;24(1):1-3. doi: 10.1097/phh.0000000000000691
45. Wolfenden L, Mooney K, Gonzalez S, et al. Increased use of knowledge translation strategies is associated with greater research impact on public health policy and practice: an analysis of trials of nutrition, physical activity, sexual health, tobacco, alcohol and substance use interventions. *Health Res Policy Syst* 2022;20(1):15. doi: 10.1186/s12961-022-00817-2 [published Online First: 20220131]

For peer review only

For peer review only - http://bmjopen.bmj.com/site/about/guidelines.xhtml

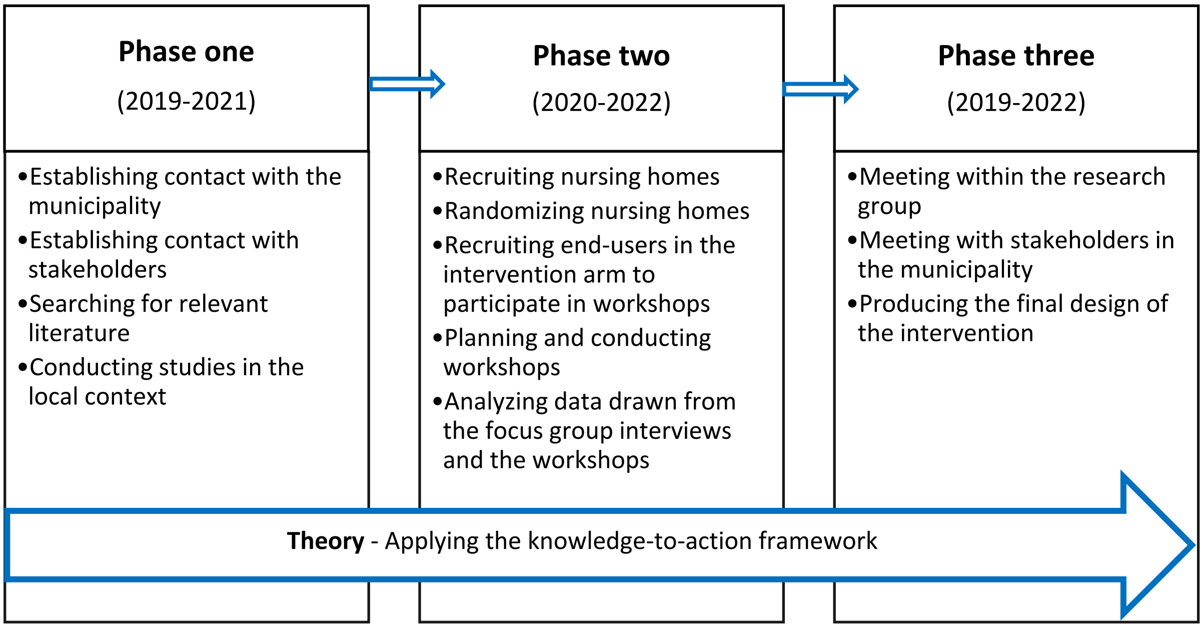

Figure 1. Phases one-three illustrate the process of developing the STAIR OF KNOWLEDGE intervention, which took place between 2019 and 2022. Although the knowledge-to-action (KTA) framework is viewed as a cycle by Graham and colleagues (2006), in this figure, the arrow illustrates the fact that the KTA framework was applied throughout phases one-three of the development process. The KTA framework was applied in an iterative and dynamic way in each phase and is described in detail in the text.

For peer review only - http://bmjopen.bmj.com/site/about/guidelines.xhtml

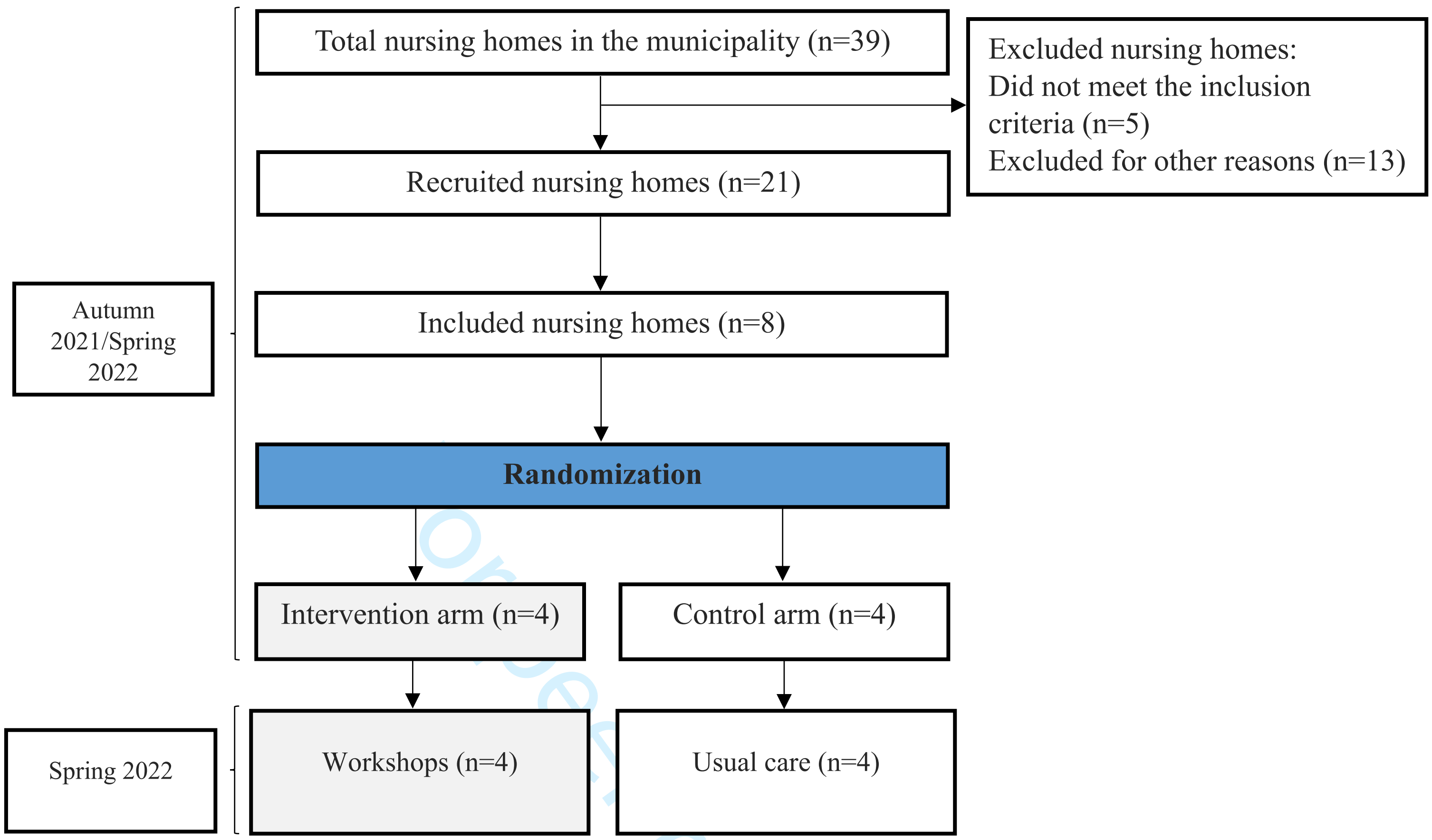

Figure 2. Flowchart of the two-arm pragmatic cluster randomized controlled trial. The flowchart illustrates phase two of the development of the STAIR OF KNOWLEDGE intervention.

For peer review only - http://bmjopen.bmj.com/site/about/guidelines.xhtml

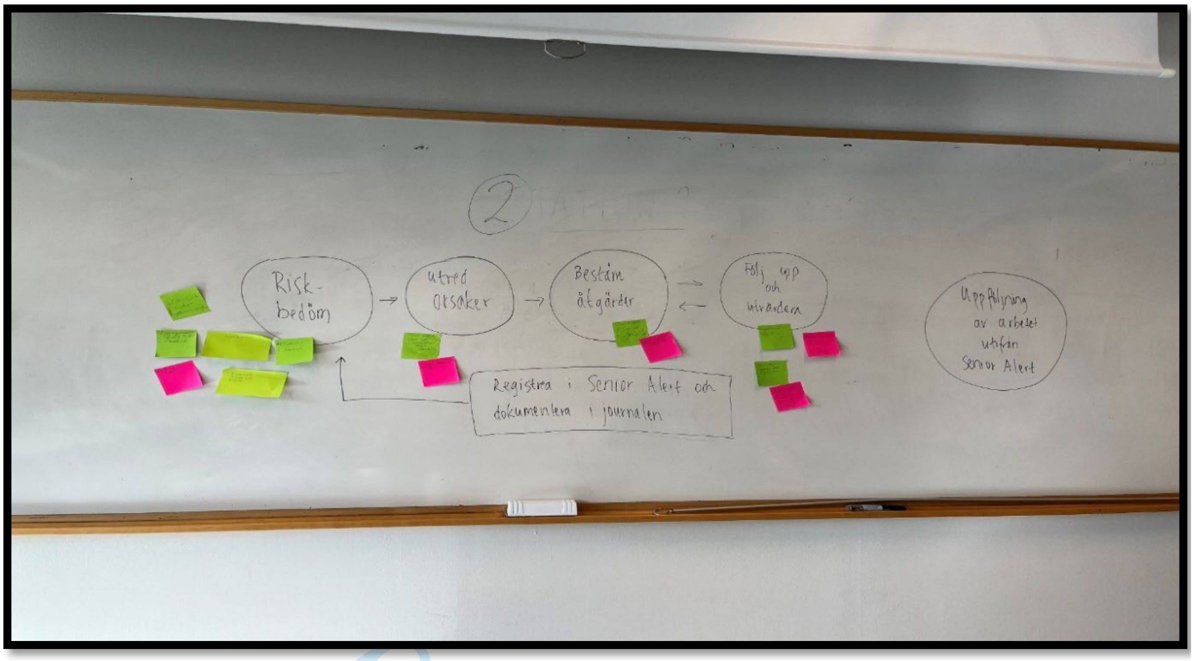

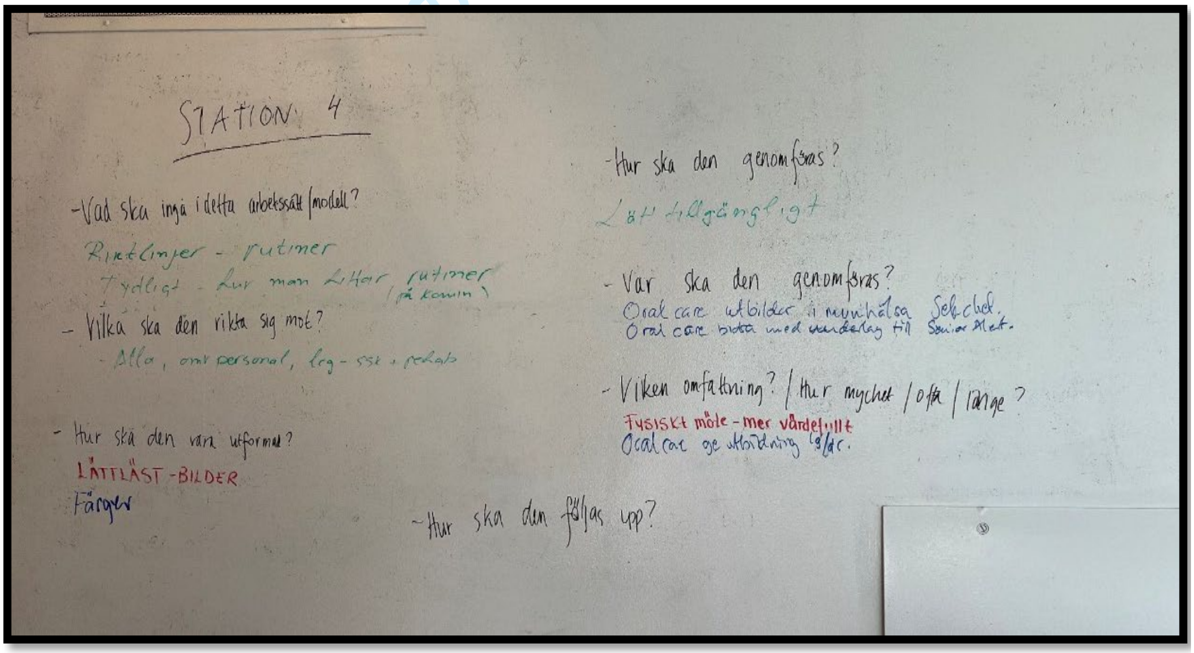

Figure 3. These pictures illustrate the end-users´ participation in stations two and four in one of the four workshops.

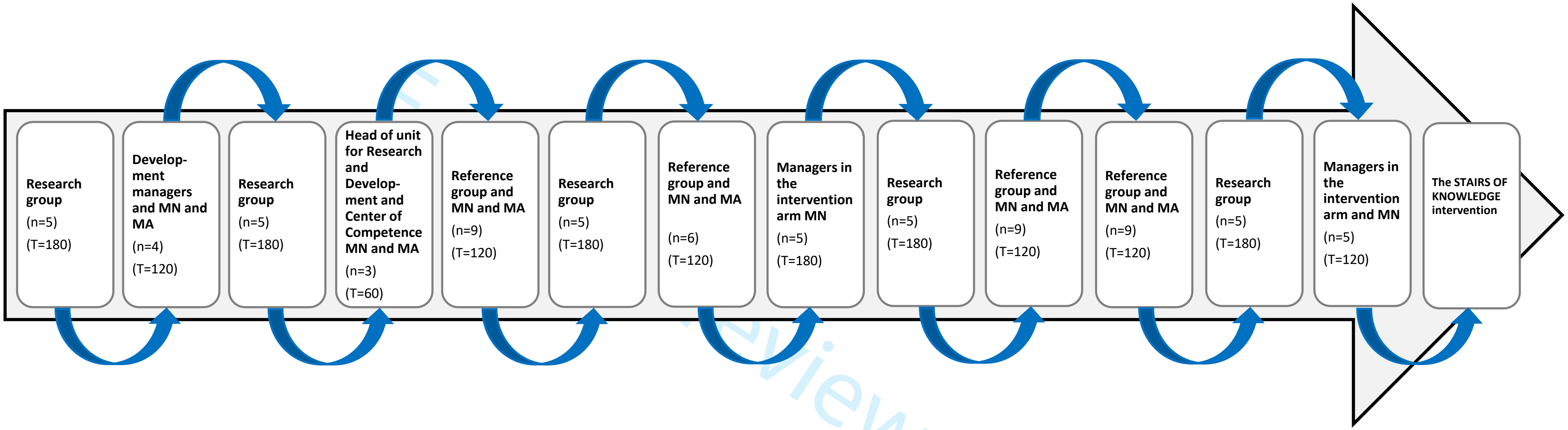

Figure 4. Note: T=how long the meeting lasted for, reported in minutes. MN=the first author. MA=the last author.
The iterative and dynamic process of designing the final outline of the STAIR OF KNOWLEDGE intervention between April and September 2022, including meetings and discussions with stakeholders. In all the meetings, the first author participated. In addition, in some meetings also the last author participated. The blue arrows illustrate that adjustments were made following each meeting.

The STAIR OF KNOWLEDGE

The STAIR OF KNOWLEDGE is addressed to all nurse aides, registered nurses and managers who work with older persons in nursing homes. The STAIR OF KNOWLEDGE aims to increase knowledge regarding the preventive care necessary to prevent the risks of falls, pressure ulcers, malnutrition and poor oral health.

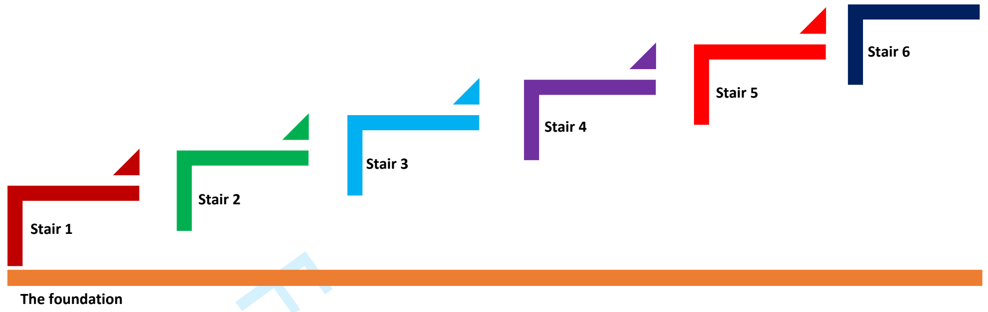

The foundation is mandatory for nurse aides, registered nurses and managers. **Stairs 1-4** and **stair 6** are mandatory for all nurse aides, registered nurses and managers. **Stair 5** is mandatory for individuals who register in Senior Alert. Follow the instructions below.

For your convenience, click the boxes as you progress through the STAIR OF KNOWLEDGE.

Foundation ☐ Stair 4a-d ☐
Stair 1a-d ☐ Stair 5 ☐
Stair 2a-d ☐ Stair 6 ☐
Stair 3 ☐

The foundation. Local working description of the entire preventive care working process.

- Link to the local working routine.

Stair 1a-d. General information regarding falls, pressure ulcers, malnutrition and poor oral health.

- Links to texts and videos regarding falls, pressure ulcers, malnutrition and poor oral health.

Stair 2a-d. Risk assessment of falls, pressure ulcers, malnutrition and poor oral health.

- Links to texts and videos regarding the risk assessment of falls, pressure ulcers, malnutrition and poor oral health.

Stair 3. Causes of falls, pressure ulcers, malnutrition and poor oral health.

- Link to text regarding the causes of falls, pressure ulcers, malnutrition and poor oral health.

Stair 4a-d. Preventive care interventions for falls, pressure ulcers, malnutrition and poor oral health.

- Links to texts regarding preventive care interventions for falls, pressure ulcers, malnutrition and poor oral health.

Stair 5. Registering in Senior Alert.

- Links to texts and videos regarding how to register in Senior Alert.

Stair 6. Inspiration week.

- Inspiration week focuses on preventive care intended to prevent the risks of falls, pressure ulcers, malnutrition and poor oral health in an inspiring and motivating way. The inspiration week will be organized by and for employees and managers. The inspiration week is preferably organized twice per year.

Figure 5. Final design of the STAIR OF KNOWLEDGE intervention.

For peer review only

TIDieR
Template for Intervention Description and Replication

The TIDieR (Template for Intervention Description and Replication) Checklist*:

Information to include when describing an intervention and the location of the information

Item number	Item<br>The codesigned STAIR OF KNOWLEDGE intervention to prevent pressure ulcers, malnutrition, poor oral health and falls among older persons in nursing homes: a complex intervention development study	Where located **<br>Primary paper (page or appendix number)	 Other † (details)
	**BRIEF NAME**		
**1.**	Provide the name or a phrase that describes the intervention.	1____________	______________
	**WHY**		
**2.**	Describe any rationale, theory, or goal of the elements essential to the intervention.	4-5 + 7______	______________
	**WHAT**		
**3.**	Materials: Describe any physical or informational materials used in the intervention, including those provided to participants or used in intervention delivery or in training of intervention providers. Provide information on where the materials can be accessed (e.g. online appendix, URL).	13-15_______	______________
**4.**	Procedures: Describe each of the procedures, activities, and/or processes used in the intervention, including any enabling or support activities.	13-15_______	______________
	**WHO PROVIDED**		
**5.**	For each category of intervention provider (e.g. psychologist, nursing assistant), describe their expertise, background and any specific training given.	5-6 + 13 + Figure 4___	______________
	**HOW**		
**6.**	Describe the modes of delivery (e.g. face-to-face or by some other mechanism, such as internet or telephone) of the intervention and whether it was provided individually or in a group.	13-15________	______________
	**WHERE**		

For peer review only - http://bmjopen.bmj.com/site/about/guidelines.xhtml

**7.**	Describe the type(s) of location(s) where the intervention occurred, including any necessary infrastructure or relevant features.	13-15________	______________
	**WHEN and HOW MUCH**		
**8.**	Describe the number of times the intervention was delivered and over what period of time including the number of sessions, their schedule, and their duration, intensity or dose.	13-15________	______________
	**TAILORING**		
**9.**	If the intervention was planned to be personalised, titrated or adapted, then describe what, why, when, and how.	15___________	______________
	**MODIFICATIONS**		
**10.‡**	If the intervention was modified during the course of the study, describe the changes (what, why, when, and how).	N/A_________	______________
	**HOW WELL**		
**11.**	Planned: If intervention adherence or fidelity was assessed, describe how and by whom, and if any strategies were used to maintain or improve fidelity, describe them.	N/A________	______________
**12.‡**	Actual: If intervention adherence or fidelity was assessed, describe the extent to which the intervention was delivered as planned.	N/A__________	______________

For peer review only

** **Authors** - use N/A if an item is not applicable for the intervention being described. **Reviewers** – use '?' if information about the element is not reported/not sufficiently reported.

† If the information is not provided in the primary paper, give details of where this information is available. This may include locations such as a published protocol or other published papers (provide citation details) or a website (provide the URL).

‡ If completing the TIDieR checklist for a protocol, these items are not relevant to the protocol and cannot be described until the study is complete.

* We strongly recommend using this checklist in conjunction with the TIDieR guide (see *BMJ* 2014;348:g1687) which contains an explanation and elaboration for each item.

* The focus of TIDieR is on reporting details of the intervention elements (and where relevant, comparison elements) of a study. Other elements and methodological features of studies are covered by other reporting statements and checklists and have not been duplicated as part of the TIDieR checklist. When a **randomised trial** is being reported, the TIDieR checklist should be used in conjunction with the CONSORT statement (see www.consort-statement.org) as an extension of **Item 5 of the CONSORT 2010 Statement.** When a **clinical trial protocol** is being reported, the TIDieR checklist should be used in conjunction with the SPIRIT statement as an extension of **Item 11 of the SPIRIT 2013**

TIDieR checklist

For peer review only - http://bmjopen.bmj.com/site/about/guidelines.xhtml

Statement (see www.spirit-statement.org). For alternate study designs, TIDieR can be used in conjunction with the appropriate checklist for that study design (see www.equator-network.org).

For peer review only

For peer review only - http://bmjopen.bmj.com/site/about/guidelines.xhtml

GUIDED – a guideline for reporting for intervention development studies.

Supplementary File 1: Blank Checklist

Item description	Explanation	Page in manuscript where item is located	Other*
1. Report the context for which the intervention was developed.	Understanding the context in which an intervention was developed informs readers about the suitability and transferability of the intervention to the context in which they are considering evaluating, adapting or using the intervention. Context here can include place, organisational and wider socio-political factors that may influence the development and/or delivery of the intervention (15).	Page 6 (Study context and setting)	
2. Report the purpose of the intervention development process.	Clearly describing the purpose of the intervention specifies what it sets out to achieve. The purpose may be informed by research priorities, for example those identified in systematic reviews, evidence gaps set out in practice guidance such as The National Institute for Health and Care Excellence or specific prioritisation exercises such as those undertaken with patients and practitioners through the James Lind Alliance.	Page 4-5 (Introduction) Page 7-13) Development of the intervention)	
3. Report the target population for the intervention development process.	The target population is the population that will potentially benefit from the intervention – this may include patients, clinicians, and/or members of the public. If the target population is clearly described then readers will be able to understand the relevance of the intervention to their own research or practice. Health inequalities, gender and ethnicity are features of the target population that may be relevant to intervention development processes.	Page 7-13 (Development of the intervention) Page 13-16 (Results)	
4. Report how any published intervention development approach contributed to the development process	Many formal intervention development approaches exist and are used to guide the intervention development process (e.g. 6Squid (16) or The Person Based Approach to Intervention Development (17)). Where a formal intervention development approach is used, it is helpful to describe the process that was followed, including any deviations. More general approaches to intervention development also exist and have been categorised as follows (3):- Target Population-centred intervention development; evidence and theory-based intervention development; partnership intervention development; implementation-based intervention development; efficacy-based intervention development; step or phased-based intervention development; and intervention-specific intervention development (3). These approaches do not always have specific guidance that describe their use. Nevertheless, it is helpful to give a rich description of how any published approach was operationalised	Page 4-5 (Introduction) Page 6 (Study design)	
5. Report how evidence from different sources informed the intervention development process.	Intervention development is often based on published evidence and/or primary data that has been collected to inform the intervention development process. It is useful to describe and reference all forms of evidence and data that have informed the development of the intervention because evidence bases can change rapidly, and to explain the manner in which the evidence and/or data was used. Understanding what evidence was and was not available at the time of intervention development can help readers to assess transferability to their current situation.	Page 7-13 (Development of the intervention)	
6. Report how/if published theory informed the intervention development process.	Reporting whether and how theory informed the intervention development process aids the reader's understanding of the theoretical rationale that underpins the intervention. Though not mentioned in the e-Delphi or consensus meeting, it became increasingly apparent through the development of our guidance that this theory item could relate to either existing published theory or programme theory	Page 4-5 (Introduction) Page 6 (Study design) Page 7 (Theory)	
7. Report any use of components from an existing intervention in the current intervention development process.	Some interventions are developed with components that have been adopted from existing interventions. Clearly identifying components that have been adopted or adapted and acknowledging their original source helps the reader to understand and distinguish between the novel and adopted components of the new intervention.	The intervention is based on exising evidence-based knowledge in Senior Alert. See figure 5.	
8. Report any guiding principles, people or factors that were prioritised when making decisions during the intervention development process.	Reporting any guiding principles that governed the development of the application helps the reader to understand the authors' reasoning behind the decisions that were made. These could include the examples of particular populations who views are being considered when designing the intervention, the modality that is viewed as being most appropriate, design features considered important for the target population, or the potential for the intervention to be scaled up.	Page 7-13 (Development of the intervention)	

For peer review only

For peer review only - http://bmjopen.bmj.com/site/about/guidelines.xhtml

Duncan E, *et al. BMJ Open* 2020; 10:e033516. doi: 10.1136/bmjopen-2019-033516

For peer review only

Item description	Explanation	Page in manuscript where item is located	Other*
9. Report how stakeholders contributed to the intervention development process.	Potential stakeholders can include patient and community representatives, local and national policy makers, health care providers and those paying for or commissioning health care. Each of these groups may influence the intervention development process in different ways. Specifying how differing groups of stakeholders contributed to the intervention development process helps the reader to understand how stakeholders were involved and the degree of influence they had on the overall process. Further detail on how to integrate stakeholder contributions within intervention reporting are available (19).	Page 7-13 (Development of the intervention)	
10. Report how the intervention changed in content and format from the start of the intervention development process.	Intervention development is frequently an iterative process. The conclusion of the initial phase of intervention development does not necessarily mean that all uncertainties have been addressed. It is helpful to list remaining uncertainties such as the intervention intensity, mode of delivery, materials, procedures, or type of location that the intervention is most suitable for. This can guide other researchers to potential future areas of research and practitioners about uncertainties relevant to their healthcare context.	Page 12 (Analysed the data from the focus group interviews and workshops) + Page 12-13 (Third phase)	
11. Report any changes to interventions required or likely to be required for subgroups.	Specifying any changes that the intervention development team perceive are required for the intervention to be delivered or tailored to specific sub groups enables readers to understand the applicability of the intervention to their target population or context. These changes could include changes to personnel delivering the intervention, to the content of the intervention, or to the mode of delivery of the intervention.	Page 12 (Analysed the data from the focus group interviews and workshops) + Page 12-13 (Third phase) + Page 13-16 (Results)	
12. Report important uncertainties at the end of the intervention development process.	Intervention development is frequently an iterative process. The conclusion of the initial phase of intervention development does not necessarily mean that all uncertainties have been addressed. It is helpful to list remaining uncertainties such as the intervention intensity, mode of delivery, materials, procedures, or type of location that the intervention is most suitable for. This can guide other researchers to potential future areas of research and practitioners about uncertainties relevant to their healthcare context.	Page 16-18 (Discussion)	
13. Follow TIDieR guidance when describing the developed intervention.	Interventions have been poorly reported for a number of years. In response to this, internationally recognized guidance has been published to support the high quality reporting of health care? interventions[5]and public health interventions[14]. This guidance should therefore be followed when describing a developed intervention.	The description of the intervention followsTIDieR	
14. Report the intervention development process in an open access format.	Unless reports of intervention development are available people considering using an intervention cannot understand the process that was undertaken and make a judgement about its appropriateness to their context. It also limits cumulative learning about intervention development methodology and observed consequences at later evaluation, translation and implementation stages. Reporting intervention development in an open access (Gold or Green) publishing format increases the accessibility and visibility of intervention development research and makes it more likely to be read and used. Potential platforms for open access publication of intervention development include open access journal publications, freely accessible funder reports or a study web-page that details the intervention development process.	Submitted to the BMJ Open	

*e.g. if item is reported elsewhere, then the location of this information can be stated here.

For peer review only - http://bmjopen.bmj.com/site/about/guidelines.xhtml

Duncan E, *et al. BMJ Open* 2020; 10:e033516. doi: 10.1136/bmjopen-2019-033516

BMJ Open

The STAIR OF KNOWLEDGE – a codesigned intervention to prevent pressure ulcers, malnutrition, poor oral health and falls among older persons in nursing homes in Sweden: development of a complex intervention

Journal:	*BMJ Open*
Manuscript ID	bmjopen-2023-072453.R2
Article Type:	Original research
Date Submitted by the Author:	24-May-2023
Complete List of Authors:	Neziraj, Merita; Malmo University, Department of Care Science, Faculty of Health and Society<br>Axelsson, Malin; Malmo University, Department of Care Science, Faculty of Health and Society<br>Kumlien, Christine ; Malmo University, Department of Care Science, Faculty of Health and Society; Skanes universitetssjukhus Malmo, Department of Cardio-Thoracic and Vascular Surgery, Skane University Hospital, Malmö, Sweden<br>Hellman, Peter; Malmo University, Department of Care Science, Faculty of Health and Society<br>Andersson, Magdalena; Unit of Research and development and competence centre, City of Malmö, Sweden, Health and Social Care, Strategic Development
**Primary Subject Heading**:	Nursing
Secondary Subject Heading:	Nursing
Keywords:	PREVENTIVE MEDICINE, Aging, Nursing Care

SCHOLARONE™
Manuscripts

I, the Submitting Author has the right to grant and does grant on behalf of all authors of the Work (as defined in the below author licence), an exclusive licence and/or a non-exclusive licence for contributions from authors who are: i) UK Crown employees; ii) where BMJ has agreed a CC-BY licence shall apply, and/or iii) in accordance with the terms applicable for US Federal Government officers or employees acting as part of their official duties; on a worldwide, perpetual, irrevocable, royalty-free basis to BMJ Publishing Group Ltd ("BMJ") its licensees and where the relevant Journal is co-owned by BMJ to the co-owners of the Journal, to publish the Work in this journal and any other BMJ products and to exploit all rights, as set out in our licence.

The Submitting Author accepts and understands that any supply made under these terms is made by BMJ to the Submitting Author unless you are acting as an employee on behalf of your employer or a postgraduate student of an affiliated institution which is paying any applicable article publishing charge ("APC") for Open Access articles. Where the Submitting Author wishes to make the Work available on an Open Access basis (and intends to pay the relevant APC), the terms of reuse of such Open Access shall be governed by a Creative Commons licence – details of these licences and which Creative Commons licence will apply to this Work are set out in our licence referred to above.

Other than as permitted in any relevant BMJ Author's Self Archiving Policies, I confirm this Work has not been accepted for publication elsewhere, is not being considered for publication elsewhere and does not duplicate material already published. I confirm all authors consent to publication of this Work and authorise the granting of this licence.

For peer review only

Title

The STAIR OF KNOWLEDGE – a codesigned intervention to prevent pressure ulcers, malnutrition, poor oral health and falls among older persons in nursing homes in Sweden: development of a complex intervention

Merita Neziraj[1]

Email: merita.neziraj@mau.se

Malin Axelsson[1]

Email: malin.axelsson@mau.se

Christine Kumlien[1, 2]

Email: christine.kumlien@mau.se

Peter Hellman[1]

Email: peter.hellman@mau.se

Magdalena Andersson[3]

Email: magdalena.andersson@malmo.se

[1]*Department of Care Science, Faculty of Health and Society, Malmö University, Sweden*

[2]*Department of Cardio-Thoracic and Vascular Surgery, Skane University Hospital, Malmö, Sweden*

[3]*Department of Health and Social Care, Strategic Development, Unit of Research and development and competence centre, City of Malmö, Sweden*

Corresponding Author: Merita Neziraj. merita.neziraj@mau.se

For peer review only

Abstract

Objectives: To describe the development of a codesigned complex intervention intended to prevent the risks of pressure ulcers, malnutrition, poor oral health and falls among older persons in nursing homes.

Design: A complex intervention development study. The development of the intervention was conducted in three phases. We established contact with stakeholders in the municipality, updated us of current status of the literature in this area and conducted studies in the local context (1). We codesigned the intervention in workshops together with end users (2). We codesigned the final outline of the intervention in an iterative process with stakeholders (3).

Setting: Nursing homes in the municipality in southern Sweden.

Participants: End users (n=16) in nursing homes (n=4) codesigned the intervention together with the research group in workshops (n=4) in March-April 2022. Additionally, stakeholders (n=17) who were considered to play an important role in developing the intervention participated throughout this process. Data were analysed using reflexive thematic analysis.

Results: Four workshops were conducted with end users (n=16) and thirteen meetings with stakeholders (n=12) were held during the development process. The intervention aims to bridge the evidence-practice gap regarding the preventive care process of the risks of pressure ulcers, malnutrition, poor oral health and falls among older persons in nursing homes. The intervention is aimed at end users, lasts for three weeks and is divided into two parts. First, end users obtain knowledge on their own by following written instructions. Second, they meet, interact and discuss the knowledge acquired during part one.

Conclusion: The intervention is robustly developed and thoroughly described. The study highlights the extensive process that is necessary for developing tailored complex interventions. The description of the entire development process may enhance the replicability of this intervention. The intervention needs to be tested and evaluated in an upcoming feasibility study.

Trial number: Clinical Trial NCT05308862.

For peer review only - http://bmjopen.bmj.com/site/about/guidelines.xhtml

Strengths

- Inspired by the Medical Research Council guidelines for complex intervention, a robust development process was undertaken based on the literature and research conducted in the local context prior to developing the complex intervention.
- A complex intervention was codesigned both with and for nurse aides, registered nurses and managers in workshops. Additionally, key persons working in the municipality were engaged in the development of this tailored intervention.
- To bridge the evidence-practice gap regarding the risks of pressure ulcers, malnutrition, poor oral health and falls among older persons in nursing homes, knowledge translation strategies were applied during the development process in accordance with the action part of the knowledge-to-action framework.
- A thorough description of the entire development process may enhance the replicability of the current intervention.

Limitations

- One limitation of the development process was that this design is time- and resource-consuming. On the other hand, this was necessary to develop a tailored complex intervention that might enhance the likelihood of successful implementation. The transferability of the tailored intervention to other nursing homes might also be a limitation.

For peer review only

Introduction

There remains an evidence-practice gap in preventing the risks of pressure ulcers, malnutrition, poor oral health and falls among older persons in nursing homes[1 2]. These health risks cause a major burden for older persons[3] and they are costly for the health care system[4]. Since older persons are more vulnerable to these health risks[5] and considering the increasing ageing population globally, particularly with regard to older persons aged 80 years or older[6], evidence-based preventive work is crucial to manage this demographic challenge and, importantly, these health risks among older persons.

In Sweden, there is a national quality register, Senior Alert, providing an individualized, standardized, structured and systematic preventive care work process for older persons 65 years or older who are at risk of pressure ulcers, malnutrition, poor oral health and falls[7]. Senior Alert provides evidence-based knowledge aimed at preventing these health risks to enable a healthy ageing among older persons[8]; in addition, it can increase cost efficiency[9]. However, a lack of knowledge among those working with older persons has been identified as one major challenge regarding to preventive work[2 10]. As a result, these health risks continue to be prevalent[7]. For instance, approximately every third older person living in a nursing home faces at least one of these health risks, and every tenth older person faces all four of these health risks[1]. Additionally, not all older persons who are at risk have planned care interventions[11 12] and there is a mismatch between identified risks and planned and performed care interventions[13 14], thus indicating an evidence-practice gap and consequently, highlighting the urgent need of translating knowledge into practice.

Nevertheless, this is not unique to Sweden or this context; in contrast, health systems worldwide face the shared challenge of translating knowledge into practice[15]. Knowledge translation has been defined as *"a dynamic and iterative process that includes synthesis, dissemination, exchange and ethically sound application of knowledge to improve health care of people in the country, provide more effective health service and products and strengthen the health care system",* p. 165[16]. Ineffective knowledge translation can result in an evidence-practice gap[17] and, worryingly, lead to situations in which patients are denied interventions that have been proven to be beneficial[18], which in turn can result in a reduction in their quality of life[19].

For peer review only

For peer review only - http://bmjopen.bmj.com/site/about/guidelines.xhtml

To bridge this evidence-practice gap, conceptual frameworks are recommended[20]. The knowledge-to-action (KTA) framework is intended to help the parties involved in the process of knowledge translation[18]. The KTA framework is also appropriate when addressing an evidence-practice gap[15] and conducting pragmatic research[18].

As a part of translating knowledge into practice and promoting knowledge use by end users[21], the engagement of both researchers and stakeholders in research is crucial[22]. Engaging stakeholders at an early stage in the development of solutions that can be applied to real world settings is essential according to the Medical Research Council´s (MRC) framework for complex interventions[23]. Complex interventions have multiple components, target multiple groups or levels of an organization and attempt to affect multiple outcomes[23]. Additionally, for complex interventions to be most useful to end users, the local context must be taken into account[24]. Since it is well underpinned that organizational factors hinder preventive work in nursing homes[2 25], considering and understanding the local context and integrating it into the process of intervention development is crucial[26].

Consequently, change in the practices of nursing homes is considered to be complex[27], but if complex interventions are tailored to the local context[28], including the targets of the intervention[23 24] and is directly relevant to them[29], such interventions could be successful.

Aim

The aim of this study was to describe the development of a codesigned complex intervention intended to prevent pressure ulcers, malnutrition, poor oral health and falls among older persons in nursing homes.

Methods

Definitions

Nursing homes were defined based on the definition provided by Neziraj et al. (2021)[1]: residential care homes where older persons live and receive municipal health care.

Health care personnel and managers were defined based on the definition provided by Neziraj et al. (2021) as follows[2]:

Nurse aide: a person with a secondary degree in nursing, involves three years of study in high school *or* a person without any formal education in nursing.

For peer review only - http://bmjopen.bmj.com/site/about/guidelines.xhtml

Registered nurse: a person with a bachelor's degree in nursing, which involves three years of study at university.

Manager: a person who is in charge of nurse aides or registered nurses.

End users: nurse aides, registered nurses and managers working in nursing homes.

Stakeholders: key persons working in the municipality who are considered to play an important role in the development and implementation of the intervention.

Study context and setting

In nursing homes, nurse aides are the main providers of care and services and are on duty around the clock. Nurse aides work under the regulations of the Social and Services Act (SFS)[30] but are also delegated tasks according to the Health and Medical Services Act (HSL)[31], usually by registered nurses. Registered nurses guide care in nursing homes and work under the regulations of HSL[31]. In the current setting, a large town located in southern Sweden with 39 nursing homes, one registered nurse (or occasionally more depending on the size of the nursing home) is located in the nursing home during office hours but is also available at any other time. Managers who are in charge of the care and services provided by the nurse aides are located at their respective nursing homes during office hours.

For transparency, the research group (n=5) positions are reported; four of the researchers hold positions as either doctoral students (MN), associated professors (MAX), professors (CK) or senior lectures (PH) at the affiliated university. The last author (MA) is a PhD and hold the position as a research and development coordinator in the municipality where the study was conducted. All the authors are registered nurses, and two of them (MN, MA) specialize in elderly care and have worked in this context previously,

In addition, a reference group was created, which consisted of experts (n=7) drawn from the local context; nurse aide (n=1), managers in charge of nursing homes (n=2), head of managers in charge of registered nurses (n=1), development managers (n=2) and head of the nursing homes in the municipality (n=1).

Study design

The current study describes the development of a codesigned complex intervention and is a part of the PROSENIOR program (https://mau.se/en/research/projects/prosenior/). This part of the PROSENIOR program aims to develop, test and evaluate a codesigned complex

For peer review only - http://bmjopen.bmj.com/site/about/guidelines.xhtml

intervention to prevent pressure ulcers, malnutrition, poor oral health and falls among older persons living in nursing homes in a two-arm pragmatic cluster randomized trial. The main purpose of the randomization was to assess the feasibility of the procedure and will be reported in an upcoming study. The control arm was therefore not included in the current study. In the current study, the randomization aimed to invite end users allocated to the intervention arm to develop a codesigned complex intervention.

The development of the codesigned complex intervention (hereafter called the intervention) was conducted in three phases. The phases are described below. The development of the current intervention was conducted in a pragmatic paradigm as it is intended to work in a real-world setting [29]; this process was inspired by the MRC guidelines for complex interventions[24], applied the KTA framework[18] and engaged end users and stakeholders in the process of codesign[32].

We follow the guidance for reporting intervention development studies (GUIDED)[33] when describing the development of the intervention and the template for intervention description and replication (TIDieR) checklist and guide[34] when describing the intervention. We use "development" to refer to the whole process of intervention development and "design" to indicate the intervention content, format and delivery.

Patient and public involvement

Patients or informal caregivers were not involved in the research process. End users codesigned the intervention with the research group in workshops. Stakeholders were also involved in this research; they supported the research group throughout the entire development of the intervention by contributing their valuable knowledge. All engagement is described in detail in the section "Development of the intervention" below.

Development of the intervention

We developed the intervention in three phases and applied the KTA framework in all phases (Figure 1).

Theory

The KTA framework takes implementation strategies into account already in the development phase[18], which promotes and sustains practice change[15]. We applied the KTA framework because it offers a structured and systematic approach to translate knowledge into practice[18].

For peer review only - http://bmjopen.bmj.com/site/about/guidelines.xhtml

It comprises two parts: knowledge creation and the action cycle. Since evidence-based knowledge is already available to end users in the quality register Senior Alert, the action cycle was applied during the development of the current intervention. The action cycle consists of the following steps: *1. Identify the problem, identify and review selected knowledge, 2. Adapt knowledge to the local context, 3. Assess barriers to knowledge use, 4. Select and tailor implementation strategies, 5. Monitor knowledge use, 6. Evaluate the outcomes* and *7. Sustain knowledge use*[18 35]. Steps 1-4 the action cycle were applied throughout the development process of developing the intervention in an iterative, dynamic and permeable way.

Phase one

During this phase, we established contact with stakeholders in the municipality, updated us of the current status of the literature in this area and conducted studies in the local context.

Establishing contact with stakeholders in the municipality

Initially, we established contact and met with the head of the nursing homes in the municipality. The reference group was created in this phase (described in the paragraph "Study context and setting" above).

Searching for literature and conducting studies in the local context

As a part of step 1 in the KTA framework*, identify the problem, identify and review selected knowledge*, firstly, we updated us of the current status of the literature regarding prevention of pressure ulcers, malnutrition, poor oral health and falls and intervention studies in this area. Subsequently, we conducted a cross-sectional study to determine the prevalence of the risks of pressure ulcers, malnutrition, poor oral health and falls in nursing homes in southern Sweden[1].

As a part of steps 2-3 in the KTA framework, *adapt knowledge to the local context and assess barriers to knowledge use,* we conducted focus group interviews (n=5) with end users (n=21) who worked in nursing homes to prevent pressure ulcers, malnutrition, poor oral health and falls[2]. The focus group interviews lasted between 63 and 106 min (mean 83 min). A detailed description of this study and its participants is provided in Neziraj et al[2]. Additionally, we asked the end users included in our previous study[2] how an optimal intervention could be designed to prevent the risks of pressure ulcers, malnutrition, poor oral health and falls among

For peer review only - http://bmjopen.bmj.com/site/about/guidelines.xhtml

older persons in nursing homes. These particular data were targeted for the current study. Hence, these data were not reported in our previous study, but are included, analysed and reported in our current study.

Phase two

During this phase, we recruited and randomized nursing homes. Subsequently, we invited end users in the intervention arm to participate in workshops, and planned and conducted the workshops. We also analysed the specific data regarding intervention design drawn from the focus group interviews (see the previous paragraph on phase one for clarification) and the workshops.

Recruiting and randomizing nursing homes

Since the current study is a part of a two-arm pragmatic cluster randomized trial, randomization was primarily conducted at this stage to assess the feasibility of the procedure in an upcoming study. In the current study, however, randomization aimed to recruit end users in nursing homes allocated to the intervention arm to codesign an intervention together with the research group in workshops (Figure 2). The nursing home is the cluster and the unit of allocation. Due to the nature of the design, the cluster randomization of nursing homes was unblinded.

Inclusion criteria for the study were nursing homes working with and registered in the quality register Senior Alert. We recruited eligible nursing homes (n=21) to participate in the study via digital meetings. In total, eight nursing homes agreed to participate and were cluster randomized using a computerized program to either the intervention (n=4) or control arm (n=4). Subsequently, we invited end users (n=118) working in nursing homes in the intervention arm to participate in workshops intended to develop a tailored intervention together with the research group; the invitations were extended both via a digital information video and in written form. The remaining end users (n=184) working in the nursing homes who were allocated to the control arm continued with their usual care routine.

Conducting workshops

As a part of steps 2-4 in the KTA framework, *adapt knowledge to the local context, assess barriers to knowledge use* and *select and tailor implementation strategies*, we conducted workshops with end users. In total, four workshops were conducted, which featured two nurse

For peer review only - http://bmjopen.bmj.com/site/about/guidelines.xhtml

aides, one registered nurse and one manager in each workshop; the workshops were conducted over the course of four weeks (March-April 2022). The workshops were kept small to offer the end users the possibility of exhibiting activity and creativity[36]. The first author (MN) led the workshops together with one of the coauthors (all coauthors participated in one workshop each). The workshops were intended to serve as a place in which participants could learn together and discuss the design of the intervention in four different stations (Table 1). The end users engaged in active discussion and wrote creative ideas and suggestions on the walls and the board in a lecture hall designed for the purpose of encouraging creative pedagogy. In the first station, the end users were asked to discuss the risks of pressure ulcers, malnutrition, poor oral health and falls and the care interventions that should be applied. In the second station, they were asked to discuss and identify barriers and facilitators they had encountered in their own work regarding the preventive care process stipulated by Senior Alert (identify a risk, assess causes and plan, undertake and evaluate care intervention). Barriers were written down on pink post-it notes, while facilitators were written down on green post-it notes. These post-it notes were subsequently placed at the appropriate location on the board with regard to the predawn preventive care process. The focus of the discussions at station three was on the end users´ needs and the support they needed throughout the preventive care process. In the fourth station, they were asked to discuss the core components of the intervention, how to provide follow-ups and implementation strategies. After completing each workshop, MN photographed and briefly summarized the written data from each station. This summary was used if the end users in the subsequent workshop reached an impasse and/or discussed and wrote similar suggestions and ideas to those proposed by the end users in the previous workshop. Each workshop lasted for three hours, and the discussions were audio recorded to support the written data collection during the analysis.

For peer review only - http://bmjopen.bmj.com/site/about/guidelines.xhtml

Table 1. Workshop content (n=4).

Workshop	Station	Content	Examples of questions to discuss
Workshop 1-4	Station 1	Case regarding an older person at risk of pressure ulcers, malnutrition, poor oral health and falls living in a nursing home	• What would you have done in this case regarding these four risks?<br>• Are there any good examples? What can you learn from good examples?<br>• What additional knowledge do you need regarding these four risks in order to produce a risk assessment and provide adequate care interventions?
	Station 2	Senior Alert´s care process	• Place green/pink post-it notes on the care process regarding what works/what can be improved in your own work and workplace.
	Station 3	End users needs´ and the support they need regarding preventive work	• What do you need in your preventive work?<br>• Why is this important, and what is most important (rank 1-3)?<br>• Who needs help in the context of preventive work?<br>• Who should be involved and in what way?<br>• What is necessary for it to be feasible?<br>• How can you work better/smarter?<br>• How can you work in a more sustainable way?
	Station 4	Core components of the intervention	• What should be included in the intervention?<br>• Who should it target?<br>• How should it be designed?<br>• How much/often/for how long should the intervention take place?<br>• How should it be followed up?<br>• Where should it be implemented?<br>• How should it be implemented?

For peer review only - http://bmjopen.bmj.com/site/about/guidelines.xhtml

Analysing the data from the focus group interviews and the workshops

The analysis was guided by the six phases of reflexive thematic analysis described by Braun and Clarke[37 38]: *1. Familiarizing with the data, 2. Coding, 3. Generating initial themes, 4. Reviewing the identified themes, 5. Defining and naming the themes* and *6. Producing the report.* Thematic analysis was chosen because it facilitates a flexible analysis process but simultaneously provides researchers with the core skills they need to conduct the analysis.

To familiarize ourselves with the data, MN and MA read the transcripts from the focus group interviews, including the data specifically collected for the current study, and the written data collected from the workshops. In addition, MN listened to all the audio-recorded discussions from the workshops meticulously. During the process of reading the data, MN and MA reflected on and generated initial codes. Subsequently, MN and MA met and discussed these initial codes (1). Thereafter, MN and MA separately engaged in a process of identifying and coding entities of interest in relation to the design of the intervention, giving equal attention to all the data (2). The initial codes were then sorted into their core components in relation to the design of the intervention (3). Next, the core components were reviewed by MN to determine whether any relevant data regarding the design of the intervention had been missed (4). Subsequently, MN designed an outline of the intervention. This outline contained the intervention's proposed design, including its content, format, plan for delivery and duration. In the following step of the analysis, the entire research group met and discussed the design of the outline of the intervention. During this step, MN continuously revised the outline of the intervention following discussions within the research group (5). Then, the outline of the intervention was redesigned by MN. The redesigned outline of the intervention was then presented to the research group before it was presented to the stakeholders. The process of producing the final design of the intervention is described in phase three below (6).

Phase three

As part of steps 2-4 in the KTA framework, *adapt knowledge to the local context, assess barriers to knowledge use* and *select and tailor implementation strategies,* MN and MA met regularly with stakeholders in structured meetings to present and discuss the outline of the intervention. MN documented all the meetings. MA works within the municipality and thus facilitated contact with stakeholders who were considered to play an important role in this part of designing the intervention. Since this part of the process was dynamic and iterative and because all relevant uncertainties had not been addressed in the redesigned outline of the

For peer review only - http://bmjopen.bmj.com/site/about/guidelines.xhtml

intervention, it was helpful to meet stakeholders for the purpose of identifying and addressing the remaining uncertainties regarding the content, format, delivery and duration of the intervention. This part of the process was time-consuming and required a back-and-forth process involving meetings and discussions between MN and MA, within the entire research group and with the stakeholders. Next, the redesigned outline of the intervention was adjusted by MN in accordance with the results of these meetings and discussions (Figure 3). Finally, MN investigated whether any data from the focus group interviews and the workshops had been missed, since these data were intended to serve as the foundation for designing the final outline of the intervention. The final design of the intervention, the STAIR OF KNOWLEDGE (Figure 4), is described below.

Results

Findings from our previous studies[1 2] in phase one showed that the prevalence of the risk for pressure ulcer, malnutrition, poor oral health and falls is still high in the local context. Furthermore, findings from phase one suggested that individuals working with older persons in nursing homes need increased knowledge concerning how to prevent these health risks. Since existing evidence and knowledge concerning how to prevent these health risks is already contained in Senior Alert, the challenge seems to lie in the evidence-practice gap. Consequently, in phase two and three, a tailored intervention was codesigned with end users, stakeholders and the research group to reduce the evidence-practice gap. The final design of the intervention is presented below.

A majority of the end users (n=16) in workshops (n=4) were women (n=13), between the ages of 28-63 years (mean 53), and had worked for 3-41 years (mean 18). The meetings (n=13) with stakeholders (n=12) lasted between 60-180 min (mean 134 min).

The final design of the intervention

The final design of the intervention was described in line with the TIDieR checklist[34] (Supplementary file).

The STAIR OF KNOWLEDGE consists of *the foundation and stairs 1-6,* lasts for three weeks and is divided into two parts. Part one, including *the foundation and stairs 1-5*, takes place throughout the entire intervention period (weeks 1-3) and is delivered digitally to end users in the nursing homes via their workplace email addresses. Part two includes *stair 6* and

For peer review only - http://bmjopen.bmj.com/site/about/guidelines.xhtml

takes place during the last week of the intervention period (week 3) in the nursing homes in question (Figure 4).

The content of the intervention

Part one of the intervention: the foundation and stairs 1-5

End users emphasized uncertainties of different professionals' responsibilities regarding the preventive work. For instance, they highlighted that it is highly relevant for respective professional to know "who does what" regarding the preventive working. Hence, *the foundation* is intended to facilitate for different professionals regarding responsibilities for respective profession and working routine in the local context. *The foundation* provide end users with knowledge and awareness of how to work preventively in the context of an existing local working routine and is intended to represent "the ground to stand on".

Furthermore, end users expressed a need of increased knowledge regarding the health risks and the entire preventive working process. They stressed the importance of basic knowledge when working with older persons in nursing homes. According to end users, not all of them has basic knowledge in how to prevent these health risks among older persons. This was particularly common among temporary workers. To meet their need, *stairs 1-4* provide the end users with general knowledge about risks of pressure ulcers, malnutrition, poor oral health and falls according to the care process suggested by the quality register Senior Alert *(stair 1),* risk assessment instruments *(stair 2),* the underlying causes of these risks *(stair 3)* and preventive care interventions (*stair 4)*. *Stairs 1-5* provide end users with website links that allow them to both read texts and watch videos. *Stairs 1-4* are mandatory for all professionals. *Stair 5* provides end users with knowledge of how to register in the quality register Senior Alert and is mandatory only for users who have access to and the responsibility to register in the quality register Senior Alert.

Part two of the intervention: stair 6

Although it was necessary for end users to increase their knowledge on their own regarding the preventive work, they particularly highlighted the importance of physical meetings. This was also stressed as important by stakeholders and was considered as a complement to the first part of the intervention. Therefore, in part two, *stair 6*, a facilitator (MN) meets with end users to interact with them and discuss the knowledge acquired during part one. The meetings will be structured including discussions based on different cases related to pressure ulcers,

For peer review only - http://bmjopen.bmj.com/site/about/guidelines.xhtml

malnutrition, poor oral health and falls. End users will also perform risk assessments, identify the underlying causes and plan accurate care interventions based on these cases. Additionally, end users will identify environmental risk factors related to the risks of pressure ulcers, malnutrition, poor oral health and falls in their own workplace. They will also discuss and generate ideas how to follow up on the preventive care process on an organizational level. This part of the intervention is intended to inspire end users to prevent pressure ulcers, malnutrition, poor oral health and falls among older persons in nursing homes.

The format of the intervention

Part one of the intervention: the foundation and stairs 1-5

From end users' perspective, it was important with a clear format. They expressed a need of a structured, readable and colourful working "manual". Hence, the format of the intervention is designed as colourful stair with the intention to visualize the entire preventive working process. To enhance the structure, end users are provided with written instructions in respective stair. Furthermore, stakeholder emphasized the need of a "self-check box" for end users when completing the foundation and stairs in the intervention. Stakeholder believed that this could increase participation and involvement among end users. Since both end users and stakeholders stressed that there are many end users that do not have the Swedish language as their native language, the language is adjusted to suit the local context. Furthermore, end users expressed that the format of the intervention should consider different ways of learning. This was also highlighted as important by stakeholders. Hence, the format consist of both reading texts and watching videos. Moreover, end users and stakeholders emphasized that a digital intervention could be a sustainable solution.

Part two of the intervention: stair 6

End users and stakeholder were in agreement that it is necessary to meet and discuss. Therefore, in part two of the intervention, end users meet in their respective nursing home. Also, the format of this part of the intervention was designed as an inspiration to raise awareness of the preventive work among end users.

The delivery of the intervention

Part one of the intervention: the foundation and stairs 1-5

For peer review only - http://bmjopen.bmj.com/site/about/guidelines.xhtml

The intervention will be delivered via email to managers in nursing homes. Subsequently, respective manager will forward the intervention via workplace email addresses to nurse aides and registered nurses. The end users highlighted that some learn better individually while others learn better in group. Therefore, they are permitted to choose if they want to read texts and watch videos individually and/or in group. *The foundation and stairs 1-5* is anticipated to take approximately 10 minutes, 60 minutes, 20 minutes, 10 minutes, 30 minutes and 60 minutes respectively for end users to complete. End users can choose to complete this part of intervention at once or divide it during working hours.

Part two of the intervention: stair 6

Part two of the intervention will be delivered by a facilitator (MN) who will moderate sessions lasting approximately 30 minutes each, Monday-Friday, in the nursing homes in question. If end users participate in all the sessions during this week, the planned amount of time is two and half hours for each end user.

Discussion

The current codesigned complex intervention, the STAIR OF KNOWLEDGE, was developed together with end users in workshops in an active and creative way. Stakeholders were also engaged in an iterative and dynamic way throughout the development of the intervention, as an important part of undertaking implementation strategies already in the development phase[39]. As recommended by the MRC framework[24], we meticulously considered the relationship between the intervention and its context when developing the intervention. Furthermore, we followed the strategies for knowledge translation included in the KTA framework[18]. Hence, the strengths exhibited by the development of this complex intervention lie in the fact that it was developed both together with and for end users and engaged stakeholders who are considered to play an important role in the development and implementation process. The current intervention is intended to work in a real-world setting and aims to bridge the evidence-practice gap regarding the process of preventing the risks of pressure ulcers, malnutrition, poor oral health and falls; ultimately, this intervention may reduce these risks among older persons in nursing homes.

When developing new intervention, the value of the used design process cannot be understated[40]. In fact, engagement of end users in a creative environment have been linked to more robust research and development efforts, which in turn may drive more successful

For peer review only - http://bmjopen.bmj.com/site/about/guidelines.xhtml

interventions outcome[40]. Hence, the benefits of codesign are potentially substantial[41]. For instance, engaging end users and stakeholders as design partners to the research group could ensure that the intervention exhibits a better fit to their needs[32]. Engaging end users and stakeholders early enables their experiences and requirements to be taken into account at the start rather than a situation in which the researchers presume to know what is required[39]. In the current development process, although end users´ and stakeholders´ engagement ranged in intensity from relatively passive to highly active, their engagement pervaded the entire development process, and important decisions regarding the intervention design were made by considering their input. Furthermore, because we engaged end users and stakeholders, the current intervention was based on their own experiences regarding the evidence and knowledge that are necessary throughout the entire process of preventing the risks of pressure ulcers, malnutrition, poor oral health and falls. Engaging end users and stakeholders during the developing process[42] was also important in light of the local context since this enabled us to identify facilitators and barriers in the environment in which the intervention will eventually be implemented[26].

A recent scoping review investigating education interventions for health professionals on fall prevention in health care settings[43], highlighted that health professional education to prevent fall is important. Nevertheless, the scoping review concluded that there are no uniform education design principles utilized to date[43]. Another review found that it was uncertain whether education delivered in different format such as didactic or video-based format makes a difference to health professionals´ knowledge of pressure ulcers prevention. However, education format in the current developed intervention was designed to fit end users' needs and suit the local context, which may have benefits for the outcome.

Considering and understanding the local context is also crucial when addressing an evidence-practice gap[24]. In this case, knowledge concerning the process of preventing the risks of pressure ulcers, malnutrition, poor oral health and falls is already contained in the quality register Senior Alert, but this evidence has not been fully translated into practice. Thus, we focused on translating the existing knowledge contained in Senior Alert into practice. However, if this knowledge is to be implemented effectively[44], it is crucial to employ a conceptual framework[20]. Therefore, we chose the KTA framework because it provided us with knowledge translation strategies to reduce the evidence-practice gap[18], and it was suitable since the quality register Senior Alert is already in use. Furthermore, adapting

For peer review only - http://bmjopen.bmj.com/site/about/guidelines.xhtml

knowledge to the local context and assessing barriers to knowledge use may enable the research to have a greater impact[45], which could in turn reduce the evidence-practice gap.

Successful intervention development is characterized as rigorous and scientific and leads to an intervention that can be implemented in a real-world setting[33]. The robust research process used to develop the STAIR OF KNOWLEDGE intervention incorporates existing evidence, the views of end users and stakeholders[41], the local context and knowledge translation strategies. Consequently, the use of knowledge translation strategies and the engagement of end users who are embedded in the local context in the development of a tailored complex intervention both for and with them could contribute to increased knowledge and awareness of the entire process of preventive care. This may, in turn, reduce the evidence-practice gap among end users and, importantly, reduce the risk of pressure ulcers, malnutrition, poor oral health and falls among older persons in nursing homes. Furthermore, the engagement of stakeholders already in the development process is likely to facilitate the implementation of the current intervention.

Limitations

Although the development of this complex intervention has been completed, it is important to acknowledge the limitations of the development process. First, only four clusters were included in the development process. Nevertheless, since this part of the trial focused on the development of an intervention rather than its evaluation and because the clusters were recruited pragmatically, the clusters included in the trial could be considered sufficient. Second, although all end users in the intervention arm (n=118) were invited to participate in workshops, only 16 participated. However, different professionals participated in the workshops, and the discussions were energetic, active and creative. Third, although this design is creative and can generate new ideas, it is time- and rescore-consuming for all parties involved. It requires end users and stakeholders to set aside time and expend extra effort in their daily work. For researchers, this process requires careful planning to enable them to coordinate, meet with many different persons repeatedly and be responsive to all parties involved. However, although this design required the expenditure of time and resources, the engagement of end users, stakeholders and researchers is meaningful and necessary to develop successful interventions; ultimately, this design might have an impact on to prevent the risks of pressure ulcers, malnutrition, poor oral health and falls among older persons in

For peer review only - http://bmjopen.bmj.com/site/about/guidelines.xhtml

nursing homes. Furthermore, the current intervention might offer value when used by others and could likely be adjusted to and tested in similar contexts.

Conclusion

The current codesign complex intervention, the STAIR OF KNOWLEDGE, which aims to prevent the risks of pressure ulcers, malnutrition, poor oral health and falls among older persons in nursing homes, is robustly developed and thoroughly described. A careful description of the entire development process and the intervention itself can enhance the replicability of the current intervention. This article highlights the extensive process that is necessary for the development of tailored complex interventions. Finally, this codesigned complex intervention might result in more evidence-based practice concerning the entire process of preventing the risks of pressure ulcers, malnutrition, poor oral health and falls and, importantly, reduce these health risks among older persons in nursing homes. However, uncertainties regarding the intervention itself remain. Thus, the STAIR OF KNOWLEDGE must be tested and evaluated in an upcoming feasibility study before we continue to the stage of conducting a full trial evaluation.

Ethical considerations

This trial was approved by the Swedish Ethical Review Authority (DNR 2019-06414). In addition, written approval was requested and granted by the head of the department of elderly care homes in the municipality in which this trial was conducted. All end users working in eligible nursing homes were invited to participate in the workshops. Moreover, end users had the right to withdraw from participation at any stage without providing reasons and bearing any consequences. Participation in the workshops was based on written consent. The results of this trial may be considered to contribute to scientific value on good ethical grounds, and the benefits of participating in the trial outweigh the corresponding risks.

Funding statement

This research received finical support with regard to conducting the workshops from the Derbrings and Stöltens research and development foundation. The funders had no role in the research manuscript's design, conduct, analysis, interpretation or drafting.

CRediT authorship contribution statement

For peer review only - http://bmjopen.bmj.com/site/about/guidelines.xhtml

Merita Neziraj: Conceptualization, Methodology, Investigation, Writing – original draft, Writing – review & editing, Validation, Formal analysis, Visualization. **Malin Axelsson:** Conceptualization, Methodology, Investigation, Writing – review & editing, Validation, Supervision. **Christine Kumlien:** Conceptualization, Methodology, Investigation, Writing – review & editing, Validation, Supervision. **Peter Hellman:** Conceptualization, Methodology, Investigation, Writing – review & editing, Validation, Supervision. **Magdalena Andersson:** Conceptualization, Methodology, Investigation, Writing – review & editing, Validation, Formal analysis, Supervision. All the authors read and approved the final version of the manuscript.

Competing interests

The authors have no conflicts of interest.

Participant consent

All participation in the workshops was based on written consent.

Acknowledgements

We thank the nurse aides, registered nurses and managers who participated in the development of the intervention. We also thank the stakeholders who participated during this process. Their enthusiastic participation was helpful in developing the STAIR OF KNOWLEDGE intervention. The Derbring and Stölten foundation is acknowledged for its financial support.

Data sharing statement

The data that support the development of the STAIR OF KNOWLEDGE intervention are not publicly available to ensure confidentiality. All data relevant to the development are included in the article. All figures and tables included in this article are original.

For peer review only - http://bmjopen.bmj.com/site/about/guidelines.xhtml

References

1. Neziraj M, Hellman P, Kumlien C, et al. Prevalence of risk for pressure ulcers, malnutrition, poor oral health and falls: a register study among older persons receiving municipal health care in southern Sweden. *BMC Geriatrics* 2021;21(1) doi: 10.1186/s12877-021-02205-x
2. Neziraj M, Andersson M, Hellman P, et al. Prevention of pressure ulcers, malnutrition, poor oral health and falls in nursing homes: A focus group study with nurse aides, registered nurses and managers. *International Journal of Nursing Studies Advances* 2021;3(100056-) doi: 10.1016/j.ijnsa.2021.100056
3. Gorecki C, Brown JM, Nelson EA, et al. Impact of pressure ulcers on quality of life in older patients: a systematic review. *Journal of the American Geriatrics Society* 2009;57(7):1175-83.
4. Marjolein van der V, Juanita AH, Geraerds AJLM, et al. Health care costs of injury in the older population: a prospective multicentre cohort study in the Netherlands. *BMC Geriatrics* 2020;20(1):1-11. doi: 10.1186/s12877-020-01825-z
5. Bravell ME, Westerlind B, Midlov P, et al. How to assess frailty and the need for care? Report from the Study of Health and Drugs in the Elderly (SHADES) in community dwellings in Sweden. *ARCHIVES OF GERONTOLOGY AND GERIATRICS* 2011;53(1):40-45. doi: 10.1016/j.archger.2010.06.011
6. WHO. Ageing well must be a global priority 2014 [accessed 10-23 2020.
7. Trinks A, Hägglin C, Dennis N, et al. The impact of a national quality register in the analysis of risks and adverse events among older adults in nursing homes and hospital wards: a Swedish senior Alertsurvey. *Safety in Health* 2018;4(10) doi: 10.1186/s40886-018-0077-x
8. Edvinsson J, Rahm M, Trinks A, et al. Senior alert: a quality registry to support a standardized, structured, and systematic preventive care process for older adults. *Quality management in health care* 2015;24(2):96-101.
9. Rubenstein LZ, Harker JO, Salvà A, et al. Screening for undernutrition in geriatric practice: developing the short-form mini-nutritional assessment (MNA-SF). *The journals of gerontology Series A, Biological sciences and medical sciences* 2001;56(6):M366-M72.
10. Dalvand S, Ebadi A, Ghanei Gheshlagh R. Nurses' knowledge on pressure injury prevention: a systematic review and meta-analysis based on the Pressure Ulcer Knowledge Assessment Tool. *Clinical, Cosmetic and Investigational Dermatology* 2018;ume 11:613-20.
11. Annelie KGP, Rose-Marie Johansson-Pajala Ph D, Marina Arkkukangas Ph D, et al. Preventing Falls and Malnutrition among Older Adults in Municipal Residential Care in Sweden: A Registry Study. *SAGE Open Nursing* 2021;7 doi: 10.1177/23779608211026161
12. Backlund A, Holmbeck O, Kumlien C, et al. A registry study of nursing assessments, interventions and evaluations according to nutrition for persons living in municipal residential care homes. *Nursing open* 2018;5(3) doi: 10.1002/nop2.144
13. Witt S, Englander E, Kumlien C, et al. Mismatch between risk factors and preventive interventions? A register study of fall prevention among older people in one Swedish county. *International journal of older people nursing* 2018;13(4):e12209.
14. Hopewell S, Adedire O, Copsey BJ, et al. Multifactorial and multiple component interventions for preventing falls in older people living in the community. *Cochrane Database of Systematic Reviews* 2018(7) doi: 10.1002/14651858.CD012221.pub2
15. Field B, Booth A, Ilott I, et al. Using the Knowledge to Action Framework in practice: a citation analysis and systematic review. *Implement Sci* 2014;9:172. doi: 10.1186/s13012-014-0172-2 [published Online First: 2014/11/25]
16. Straus SE, Tetroe J, Graham I. Defining knowledge translation. *Cmaj* 2009;181(3-4):165-8. doi: 10.1503/cmaj.081229 [published Online First: 20090720]
17. Brownson RC, Eyler AA, Harris JK, et al. Getting the Word Out: New Approaches for Disseminating Public Health Science. *J Public Health Manag Pract* 2018;24(2):102-11. doi: 10.1097/phh.0000000000000673

For peer review only - http://bmjopen.bmj.com/site/about/guidelines.xhtml

18. Graham ID, Logan J, Harrison MB, et al. Lost in knowledge translation: time for a map? *J Contin Educ Health Prof* 2006;26(1):13-24. doi: 10.1002/chp.47 [published Online First: 2006/03/25]
19. Davis D, Evans M, Jadad A, et al. The case for knowledge translation: shortening the journey from evidence to effect. *Bmj* 2003;327(7405):33-5. doi: 10.1136/bmj.327.7405.33
20. Bowen S, Graham ID. Backwards design or looking sideways? knowledge translation in the real world Comment on "A call for a backward design to knowledge translation". *Int J Health Policy Manag* 2015;4(8):545-7. doi: 10.15171/ijhpm.2015.71 [published Online First: 20150324]
21. Eljiz K, Greenfield D, Hogden A, et al. Improving knowledge translation for increased engagement and impact in healthcare. *BMJ Open Qual* 2020;9(3) doi: 10.1136/bmjoq-2020-000983
22. Graham ID, Kothari A, McCutcheon C. Moving knowledge into action for more effective practice, programmes and policy: protocol for a research programme on integrated knowledge translation. *Implement Sci* 2018;13(1):22. doi: 10.1186/s13012-017-0700-y [published Online First: 20180202]
23. Craig P, Dieppe P, Macintyre S, et al. Developing and evaluating complex interventions: the new Medical Research Council guidance. *Bmj* 2008;337:a1655. doi: 10.1136/bmj.a1655 [published Online First: 2008/10/01]
24. Skivington K, Matthews L, Simpson SA, et al. A new framework for developing and evaluating complex interventions: update of Medical Research Council guidance. *BMJ-BRITISH MEDICAL JOURNAL* 2021;374:n2061. doi: 10.1136/bmj.n2061
25. Lannering C, Ernsth-Bravell M, Johansson L. Prevention of falls, malnutrition and pressure ulcers among older persons: nursing staff's experiences of a structured preventive care process. *Health & Social Care in the Community* 2017;25(3):1011-20. doi: 10.1111/hsc.12400
26. Beran D, Lazo-Porras M, Cardenas MK, et al. Moving from formative research to co-creation of interventions: insights from a community health system project in Mozambique, Nepal and Peru. *BMJ Global Health* 2018;3(6):e001183. doi: 10.1136/bmjgh-2018-001183
27. Rantz MJ, Flesner MK, Zwygart-Stauffacher M. Improving care in nursing homes using quality measures/indicators and complexity science. *J Nurs Care Qual* 2010;25(1):5-12. doi: 10.1097/NCQ.0b013e3181c12b0f [published Online First: 2009/12/17]
28. Per N, Susanne B. Context matters in implementation science: a scoping review of determinant frameworks that describe contextual determinants for implementation outcomes. *BMC Health Services Research* 2019;19(1):1-21. doi: 10.1186/s12913-019-4015-3
29. Zwarenstein M, Treweek S, Gagnier JJ, et al. Improving the reporting of pragmatic trials: an extension of the CONSORT statement. *BMJ* 2008;337:a2390. doi: 10.1136/bmj.a2390
30. Geurden B. Screening for Malnutrition in Healthcare. Universiteit Antwerpen (Belgium), 2014.
31. Lauque S, Arnaud-Battandier F, Mansourian R, et al. Protein-energy oral supplementation in malnourished nursing-home residents. A controlled trial. *Age & Ageing* 2000;29(1):51-56.
32. Slattery P, Saeri AK, Bragge P. Research co-design in health: a rapid overview of reviews. *Health Res Policy Syst* 2020;18(1):17. doi: 10.1186/s12961-020-0528-9 [published Online First: 20200211]
33. Duncan E, O'Cathain A, Rousseau N, et al. Guidance for reporting intervention development studies in health research (GUIDED): an evidence-based consensus study. *BMJ Open* 2020;10(4):e033516. doi: 10.1136/bmjopen-2019-033516 [published Online First: 20200408]
34. Hoffmann TC, Glasziou PP, Boutron I, et al. [Better Reporting of Interventions: Template for Intervention Description and Replication (TIDieR) Checklist and Guide]. *Gesundheitswesen* 2016;78(3):175-88. doi: 10.1055/s-0041-111066 [published Online First: 20160129]
35. Graham ID, Logan J. Innovations in knowledge transfer and continuity of care. *Can J Nurs Res* 2004;36(2):89-103.
36. Ørngreen R, Levinsen K. Workshops as a Research Methodology. *Electronic Journal of e-Learning* 2017;15(1):70-81.

For peer review only - http://bmjopen.bmj.com/site/about/guidelines.xhtml

37. Braun V, Clarke V. Using thematic analysis in psychology. *Qualitative Research in Psychology* 2006;3(2):77-101. doi: 10.1191/1478088706qp063oa
38. Braun V, Clarke V. Reflecting on reflexive thematic analysis. *Qualitative Research in Sport, Exercise and Health* 2019;11(4):589-97. doi: 10.1080/2159676X.2019.1628806
39. O'Cathain A, Croot L, Duncan E, et al. Guidance on how to develop complex interventions to improve health and healthcare. *BMJ Open* 2019;9(8):e029954. doi: 10.1136/bmjopen-2019-029954 [published Online First: 20190815]
40. Sanders EN, Stappers P. Co-creation and the new landscapes of design. *CoDesign* 2008;4(1):5-18. doi: 10.1080/15710880701875068
41. Turner KM, Rousseau N, Croot L, et al. Understanding successful development of complex health and healthcare interventions and its drivers from the perspective of developers and wider stakeholders: an international qualitative interview study. *BMJ Open* 2019;9(5):e028756. doi: 10.1136/bmjopen-2018-028756 [published Online First: 20190530]
42. O'Cathain A, Thomas KJ, Drabble SJ, et al. What can qualitative research do for randomised controlled trials? A systematic mapping review. *BMJ Open* 2013;3(6):e002889. doi: 10.1136/bmjopen-2013-002889
43. Shaw L, Kiegaldie D, Farlie MK. Education interventions for health professionals on falls prevention in health care settings: a 10-year scoping review. *BMC Geriatr* 2020;20(1):460. doi: 10.1186/s12877-020-01819-x [published Online First: 20201109]
44. Moore JB, Maddock JE, Brownson RC. The Role of Dissemination in Promotion and Tenure for Public Health. *J Public Health Manag Pract* 2018;24(1):1-3. doi: 10.1097/phh.0000000000000691
45. Wolfenden L, Mooney K, Gonzalez S, et al. Increased use of knowledge translation strategies is associated with greater research impact on public health policy and practice: an analysis of trials of nutrition, physical activity, sexual health, tobacco, alcohol and substance use interventions. *Health Res Policy Syst* 2022;20(1):15. doi: 10.1186/s12961-022-00817-2 [published Online First: 20220131]

Figure 1 - Phases one-three illustrate the process of developing the STAIR OF KNOWLEDGE intervention, which took place between 2019 and 2022. Although the knowledge-to-action (KTA) framework is viewed as a cycle by Graham and colleagues (2006), in this figure, the arrow illustrates the fact that the KTA framework was applied throughout phases one-three of the development process. The KTA framework was applied in an iterative and dynamic way in each phase and is described in detail in the text.

Figure 2 - Flowchart of the two-arm pragmatic cluster randomized controlled trial. The dashed lines illustrate an upcoming study. The current study focused on the development of the STAIR OF KNOWLEDGE intervention.

Figure 3 - Note: T=how long the meeting lasted for, reported in minutes. MN=the first author. MA=the last author.
The iterative and dynamic process of designing the final outline of the STAIR OF KNOWLEDGE intervention between April and September 2022, including meetings and discussions with stakeholders. In all the meetings, the first author participated. In addition, in some meetings also the last author participated. The blue arrows illustrate that adjustments were made following each meeting.

Figure 4 - Final design of the STAIR OF KNOWLEDGE intervention.

For peer review only - http://bmjopen.bmj.com/site/about/guidelines.xhtml

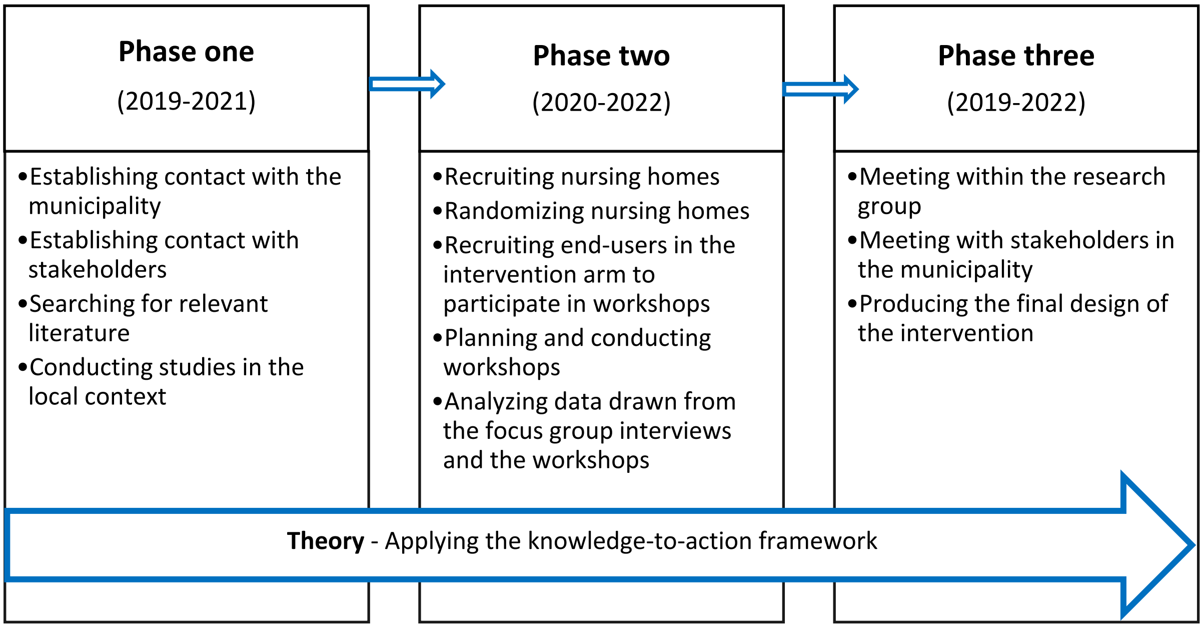

Figure 1. Phases one-three illustrate the process of developing the STAIR OF KNOWLEDGE intervention, which took place between 2019 and 2022. Although the knowledge-to-action (KTA) framework is viewed as a cycle by Graham and colleagues (2006), in this figure, the arrow illustrates the fact that the KTA framework was applied throughout phases one-three of the development process. The KTA framework was applied in an iterative and dynamic way in each phase and is described in detail in the text.

For peer review only

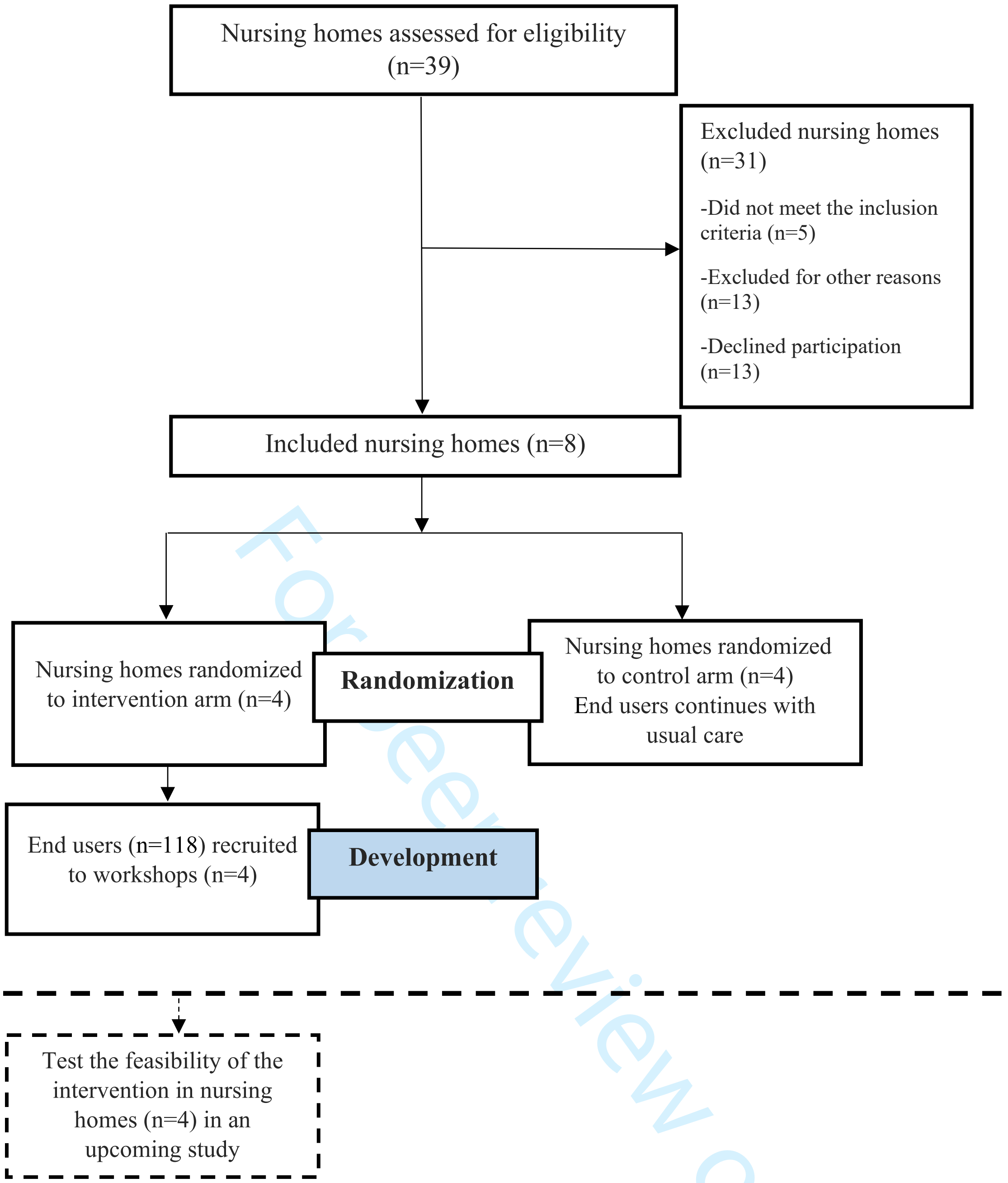

Figure 2. Flowchart of the two-arm pragmatic cluster randomized controlled trial. The dashed lines illustrate an upcoming study. The current study focused on the development of the STAIR OF KNOWLEDGE intervention.

For peer review only

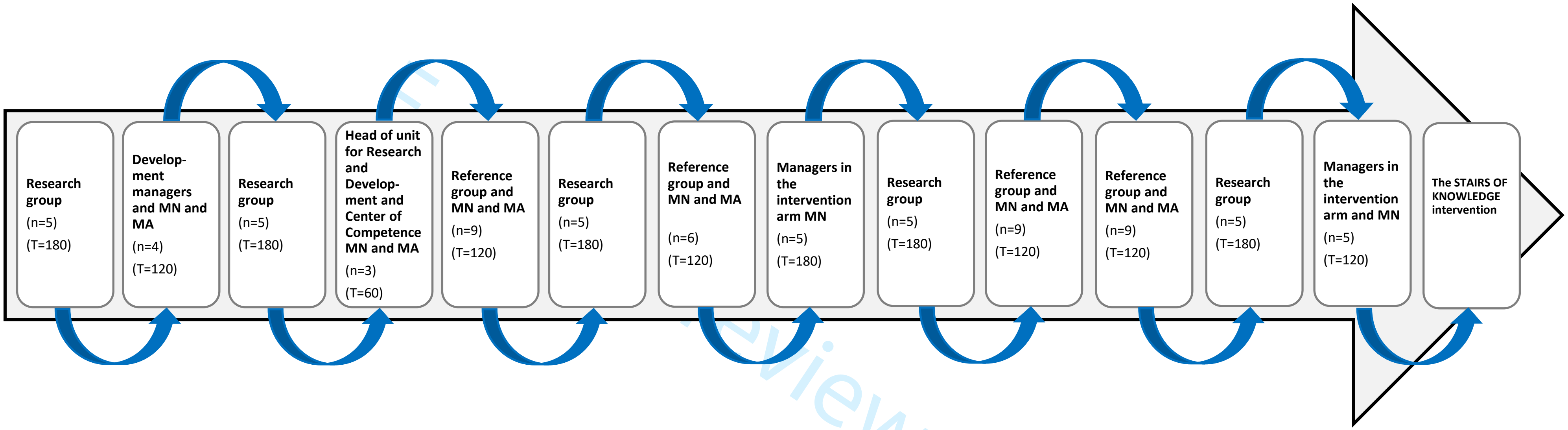

Figure 3. Note: T=how long the meeting lasted for, reported in minutes. MN=the first author. MA=the last author.
The iterative and dynamic process of designing the final outline of the STAIR OF KNOWLEDGE intervention between April and September 2022, including meetings and discussions with stakeholders. In all the meetings, the first author participated. In addition, in some meetings also the last author participated. The blue arrows illustrate that adjustments were made following each meeting.

For peer review only - http://bmjopen.bmj.com/site/about/guidelines.xhtml

The STAIR OF KNOWLEDGE

The STAIR OF KNOWLEDGE is addressed to all nurse aides, registered nurses and managers who work with older persons in nursing homes. The STAIR OF KNOWLEDGE aims to increase knowledge regarding the preventive care necessary to prevent the risks of falls, pressure ulcers, malnutrition and poor oral health.

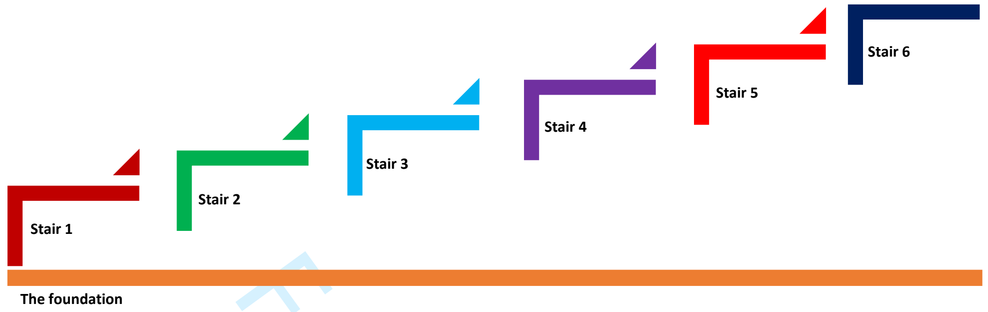

The foundation is mandatory for nurse aides, registered nurses and managers. **Stairs 1-4** and **stair 6** are mandatory for all nurse aides, registered nurses and managers. **Stair 5** is mandatory for individuals who register in Senior Alert. Follow the instructions below.

For your convenience, click the boxes as you progress through the STAIR OF KNOWLEDGE.

Foundation	☐	Stair 4a-d	☐
Stair 1a-d	☐	Stair 5	☐
Stair 2a-d	☐	Stair 6	☐
Stair 3	☐		

The foundation. Local working description of the entire preventive care working process.

- Link to the local working routine.

Stair 1a-d. General information regarding falls, pressure ulcers, malnutrition and poor oral health.

- Links to texts and videos regarding falls, pressure ulcers, malnutrition and poor oral health.

Stair 2a-d. Risk assessment of falls, pressure ulcers, malnutrition and poor oral health.

- Links to texts and videos regarding the risk assessment of falls, pressure ulcers, malnutrition and poor oral health.

Stair 3. Causes of falls, pressure ulcers, malnutrition and poor oral health.

- Link to text regarding the causes of falls, pressure ulcers, malnutrition and poor oral health.

Stair 4a-d. Preventive care interventions for falls, pressure ulcers, malnutrition and poor oral health.

- Links to texts regarding preventive care interventions for falls, pressure ulcers, malnutrition and poor oral health.

Stair 5. Registering in Senior Alert.

- Links to texts and videos regarding how to register in Senior Alert.

Stair 6. Inspiration week.

- Inspiration week focuses on preventive care intended to prevent the risks of falls, pressure ulcers, malnutrition and poor oral health in an inspiring and motivating way. The inspiration week will be organized by and for employees and managers. The inspiration week is preferably organized twice per year.

Figure 4. Final design of the STAIR OF KNOWLEDGE intervention.

For peer review only

TIDieR
Template for Intervention Description and Replication

The TIDieR (Template for Intervention Description and Replication) Checklist*:

Information to include when describing an intervention and the location of the information

Item number	Item<br>The codesigned STAIR OF KNOWLEDGE intervention to prevent pressure ulcers, malnutrition, poor oral health and falls among older persons in nursing homes: a complex intervention development study	Where located **<br>Primary paper (page or appendix number)	Other † (details)
	**BRIEF NAME**		
**1.**	Provide the name or a phrase that describes the intervention.	1____________	_______________
	**WHY**		
**2.**	Describe any rationale, theory, or goal of the elements essential to the intervention.	4-5 + 7______	______________
	**WHAT**		
**3.**	Materials: Describe any physical or informational materials used in the intervention, including those provided to participants or used in intervention delivery or in training of intervention providers. Provide information on where the materials can be accessed (e.g. online appendix, URL).	13-15_______	______________
**4.**	Procedures: Describe each of the procedures, activities, and/or processes used in the intervention, including any enabling or support activities.	13-15_______	______________
	**WHO PROVIDED**		
**5.**	For each category of intervention provider (e.g. psychologist, nursing assistant), describe their expertise, background and any specific training given.	5-6 + 13 + Figure 4___	______________
	**HOW**		
**6.**	Describe the modes of delivery (e.g. face-to-face or by some other mechanism, such as internet or telephone) of the intervention and whether it was provided individually or in a group.	13-15________	______________
	**WHERE**		

For peer review only - http://bmjopen.bmj.com/site/about/guidelines.xhtml

**7.**	Describe the type(s) of location(s) where the intervention occurred, including any necessary infrastructure or relevant features.	13-15________	______________
	**WHEN and HOW MUCH**		
**8.**	Describe the number of times the intervention was delivered and over what period of time including the number of sessions, their schedule, and their duration, intensity or dose.	13-15________	______________
	**TAILORING**		
**9.**	If the intervention was planned to be personalised, titrated or adapted, then describe what, why, when, and how.	15___________	______________
	**MODIFICATIONS**		
**10.‡**	If the intervention was modified during the course of the study, describe the changes (what, why, when, and how).	N/A_________	______________
	**HOW WELL**		
**11.**	Planned: If intervention adherence or fidelity was assessed, describe how and by whom, and if any strategies were used to maintain or improve fidelity, describe them.	N/A________	______________
**12.‡**	Actual: If intervention adherence or fidelity was assessed, describe the extent to which the intervention was delivered as planned.	N/A_________	______________

For peer review only

** **Authors** - use N/A if an item is not applicable for the intervention being described. **Reviewers** – use '?' if information about the element is not reported/not sufficiently reported.

† If the information is not provided in the primary paper, give details of where this information is available. This may include locations such as a published protocol or other published papers (provide citation details) or a website (provide the URL).

‡ If completing the TIDieR checklist for a protocol, these items are not relevant to the protocol and cannot be described until the study is complete.

* We strongly recommend using this checklist in conjunction with the TIDieR guide (see *BMJ* 2014;348:g1687) which contains an explanation and elaboration for each item.

* The focus of TIDieR is on reporting details of the intervention elements (and where relevant, comparison elements) of a study. Other elements and methodological features of studies are covered by other reporting statements and checklists and have not been duplicated as part of the TIDieR checklist. When a **randomised trial** is being reported, the TIDieR checklist should be used in conjunction with the CONSORT statement (see www.consort-statement.org) as an extension of **Item 5 of the CONSORT 2010 Statement.** When a **clinical trial protocol** is being reported, the TIDieR checklist should be used in conjunction with the SPIRIT statement as an extension of **Item 11 of the SPIRIT 2013**

For peer review only - http://bmjopen.bmj.com/site/about/guidelines.xhtml

Statement (see www.spirit-statement.org). For alternate study designs, TIDieR can be used in conjunction with the appropriate checklist for that study design (see www.equator-network.org).

For peer review only

For peer review only - http://bmjopen.bmj.com/site/about/guidelines.xhtml

GUIDED – a guideline for reporting for intervention development studies.

Supplementary File 1: Blank Checklist

Item description	Explanation	Page in manuscript where item is located	Other*
1. Report the context for which the intervention was developed.	Understanding the context in which an intervention was developed informs readers about the suitability and transferability of the intervention to the context in which they are considering evaluating, adapting or using the intervention. Context here can include place, organisational and wider socio-political factors that may influence the development and/or delivery of the intervention (15).	Page 6 (Study context and setting)	
2. Report the purpose of the intervention development process.	Clearly describing the purpose of the intervention specifies what it sets out to achieve. The purpose may be informed by research priorities, for example those identified in systematic reviews, evidence gaps set out in practice guidance such as The National Institute for Health and Care Excellence or specific prioritisation exercises such as those undertaken with patients and practitioners through the James Lind Alliance.	Page 4-5 (Introduction) Page 7-13) Development of the intervention)	
3. Report the target population for the intervention development process.	The target population is the population that will potentially benefit from the intervention – this may include patients, clinicians, and/or members of the public. If the target population is clearly described then readers will be able to understand the relevance of the intervention to their own research or practice. Health inequalities, gender and ethnicity are features of the target population that may be relevant to intervention development processes.	Page 7-13 (Development of the intervention) Page 13-16 (Results)	
4. Report how any published intervention development approach contributed to the development process	Many formal intervention development approaches exist and are used to guide the intervention development process (e.g. 6Squid (16) or The Person Based Approach to Intervention Development (17)). Where a formal intervention development approach is used, it is helpful to describe the process that was followed, including any deviations. More general approaches to intervention development also exist and have been categorised as follows (3):- Target Population-centred intervention development; evidence and theory-based intervention development; partnership intervention development; implementation-based intervention development; efficacy-based intervention development; step or phased-based intervention development; and intervention-specific intervention development (3). These approaches do not always have specific guidance that describe their use. Nevertheless, it is helpful to give a rich description of how any published approach was operationalised	Page 4-5 (Introduction) Page 6 (Study design)	
5. Report how evidence from different sources informed the intervention development process.	Intervention development is often based on published evidence and/or primary data that has been collected to inform the intervention development process. It is useful to describe and reference all forms of evidence and data that have informed the development of the intervention because evidence bases can change rapidly, and to explain the manner in which the evidence and/or data was used. Understanding what evidence was and was not available at the time of intervention development can help readers to assess transferability to their current situation.	Page 7-13 (Development of the intervention)	
6. Report how/if published theory informed the intervention development process.	Reporting whether and how theory informed the intervention development process aids the reader's understanding of the theoretical rationale that underpins the intervention. Though not mentioned in the e-Delphi or consensus meeting, it became increasingly apparent through the development of our guidance that this theory item could relate to either existing published theory or programme theory	Page 4-5 (Introduction) Page 6 (Study design) Page 7 (Theory)	
7. Report any use of components from an existing intervention in the current intervention development process.	Some interventions are developed with components that have been adopted from existing interventions. Clearly identifying components that have been adopted or adapted and acknowledging their original source helps the reader to understand and distinguish between the novel and adopted components of the new intervention.	The intervention is based on exising evidence-based knowledge in Senior Alert. See figure 5.	
8. Report any guiding principles, people or factors that were prioritised when making decisions during the intervention development process.	Reporting any guiding principles that governed the development of the application helps the reader to understand the authors' reasoning behind the decisions that were made. These could include the examples of particular populations who views are being considered when designing the intervention, the modality that is viewed as being most appropriate, design features considered important for the target population, or the potential for the intervention to be scaled up.	Page 7-13 (Development of the intervention)	

For peer review only - http://bmjopen.bmj.com/site/about/guidelines.xhtml

Duncan E, *et al. BMJ Open* 2020; 10:e033516. doi: 10.1136/bmjopen-2019-033516

Item description	Explanation	Page in manuscript where item is located	Other*
9. Report how stakeholders contributed to the intervention development process.	Potential stakeholders can include patient and community representatives, local and national policy makers, health care providers and those paying for or commissioning health care. Each of these groups may influence the intervention development process in different ways. Specifying how differing groups of stakeholders contributed to the intervention development process helps the reader to understand how stakeholders were involved and the degree of influence they had on the overall process. Further detail on how to integrate stakeholder contributions within intervention reporting are available (19).	Page 7-13 (Development of the intervention)	
10. Report how the intervention changed in content and format from the start of the intervention development process.	Intervention development is frequently an iterative process. The conclusion of the initial phase of intervention development does not necessarily mean that all uncertainties have been addressed. It is helpful to list remaining uncertainties such as the intervention intensity, mode of delivery, materials, procedures, or type of location that the intervention is most suitable for. This can guide other researchers to potential future areas of research and practitioners about uncertainties relevant to their healthcare context.	Page 12 (Analysed the data from the focus group interviews and workshops) + Page 12-13 (Third phase)	
11. Report any changes to interventions required or likely to be required for subgroups.	Specifying any changes that the intervention development team perceive are required for the intervention to be delivered or tailored to specific sub groups enables readers to understand the applicability of the intervention to their target population or context. These changes could include changes to personnel delivering the intervention, to the content of the intervention, or to the mode of delivery of the intervention.	Page 12 (Analysed the data from the focus group interviews and workshops) + Page 12-13 (Third phase) + Page 13-16 (Results)	
12. Report important uncertainties at the end of the intervention development process.	Intervention development is frequently an iterative process. The conclusion of the initial phase of intervention development does not necessarily mean that all uncertainties have been addressed. It is helpful to list remaining uncertainties such as the intervention intensity, mode of delivery, materials, procedures, or type of location that the intervention is most suitable for. This can guide other researchers to potential future areas of research and practitioners about uncertainties relevant to their healthcare context.	Page 16-18 (Discussion)	
13. Follow TIDieR guidance when describing the developed intervention.	Interventions have been poorly reported for a number of years. In response to this, internationally recognized guidance has been published to support the high quality reporting of health care? interventions[5] and public health interventions[14]. This guidance should therefore be followed when describing a developed intervention.	The description of the intervention followsTIDieR	
14. Report the intervention development process in an open access format.	Unless reports of intervention development are available people considering using an intervention cannot understand the process that was undertaken and make a judgement about its appropriateness to their context. It also limits cumulative learning about intervention development methodology and observed consequences at later evaluation, translation and implementation stages. Reporting intervention development in an open access (Gold or Green) publishing format increases the accessibility and visibility of intervention development research and makes it more likely to be read and used. Potential platforms for open access publication of intervention development include open access journal publications, freely accessible funder reports or a study web-page that details the intervention development process.	Submitted to the BMJ Open	

*e.g. if item is reported elsewhere, then the location of this information can be stated here.

For peer review only - http://bmjopen.bmj.com/site/about/guidelines.xhtml

Duncan E, *et al. BMJ Open* 2020; 10:e033516. doi: 10.1136/bmjopen-2019-033516

BMJ Open

The STAIR OF KNOWLEDGE – a codesigned intervention to prevent pressure ulcers, malnutrition, poor oral health and falls among older persons in nursing homes in Sweden: development of a complex intervention

Journal:	*BMJ Open*
Manuscript ID	bmjopen-2023-072453.R3
Article Type:	Original research
Date Submitted by the Author:	22-Jun-2023
Complete List of Authors:	Neziraj, Merita; Malmo University, Department of Care Science, Faculty of Health and Society<br>Axelsson, Malin; Malmo University, Department of Care Science, Faculty of Health and Society<br>Kumlien, Christine ; Malmo University, Department of Care Science, Faculty of Health and Society; Skanes universitetssjukhus Malmo, Department of Cardio-Thoracic and Vascular Surgery, Skane University Hospital, Malmö, Sweden<br>Hellman, Peter; Malmo University, Department of Care Science, Faculty of Health and Society<br>Andersson, Magdalena; Unit of Research and development and competence centre, City of Malmö, Sweden, Health and Social Care, Strategic Development
**Primary Subject Heading**:	Nursing
Secondary Subject Heading:	Nursing
Keywords:	PREVENTIVE MEDICINE, Aging, Nursing Care

SCHOLARONE™
Manuscripts

I, the Submitting Author has the right to grant and does grant on behalf of all authors of the Work (as defined in the below author licence), an exclusive licence and/or a non-exclusive licence for contributions from authors who are: i) UK Crown employees; ii) where BMJ has agreed a CC-BY licence shall apply, and/or iii) in accordance with the terms applicable for US Federal Government officers or employees acting as part of their official duties; on a worldwide, perpetual, irrevocable, royalty-free basis to BMJ Publishing Group Ltd ("BMJ") its licensees and where the relevant Journal is co-owned by BMJ to the co-owners of the Journal, to publish the Work in this journal and any other BMJ products and to exploit all rights, as set out in our licence.

The Submitting Author accepts and understands that any supply made under these terms is made by BMJ to the Submitting Author unless you are acting as an employee on behalf of your employer or a postgraduate student of an affiliated institution which is paying any applicable article publishing charge ("APC") for Open Access articles. Where the Submitting Author wishes to make the Work available on an Open Access basis (and intends to pay the relevant APC), the terms of reuse of such Open Access shall be governed by a Creative Commons licence – details of these licences and which Creative Commons licence will apply to this Work are set out in our licence referred to above.

Other than as permitted in any relevant BMJ Author's Self Archiving Policies, I confirm this Work has not been accepted for publication elsewhere, is not being considered for publication elsewhere and does not duplicate material already published. I confirm all authors consent to publication of this Work and authorise the granting of this licence.

For peer review only

Title

The STAIR OF KNOWLEDGE – a codesigned intervention to prevent pressure ulcers, malnutrition, poor oral health and falls among older persons in nursing homes in Sweden: development of a complex intervention

Merita Neziraj[1]

Email: merita.neziraj@mau.se

Malin Axelsson[1]

Email: malin.axelsson@mau.se

Christine Kumlien[1, 2]

Email: christine.kumlien@mau.se

Peter Hellman[1]

Email: peter.hellman@mau.se

Magdalena Andersson[3]

Email: magdalena.andersson@malmo.se

[1]*Department of Care Science, Faculty of Health and Society, Malmö University, Sweden*

[2]*Department of Cardio-Thoracic and Vascular Surgery, Skane University Hospital, Malmö, Sweden*

[3]*Department of Health and Social Care, Strategic Development, Unit of Research and development and competence centre, City of Malmö, Sweden*

Corresponding Author: Merita Neziraj. merita.neziraj@mau.se

For peer review only - http://bmjopen.bmj.com/site/about/guidelines.xhtml

Abstract

Objectives: To describe the development of a codesigned complex intervention intended to prevent the risks of pressure ulcers, malnutrition, poor oral health and falls among older persons in nursing homes.

Design: A complex intervention development study. The development of the intervention was conducted in three phases. We established contact with stakeholders in the municipality, updated us of current status of the literature in this area and conducted studies in the local context (1). We codesigned the intervention in workshops together with end users (2). We codesigned the final outline of the intervention in an iterative process with stakeholders (3).

Setting: Nursing homes in the municipality in southern Sweden.

Participants: End users (n=16) in nursing homes (n=4) codesigned the intervention together with the research group in workshops (n=4) in March-April 2022. Additionally, stakeholders (n=17) who were considered to play an important role in developing the intervention participated throughout this process. Data were analysed using reflexive thematic analysis.

Results: Four workshops were conducted with end users (n=16) and thirteen meetings with stakeholders (n=12) were held during the development process. The intervention aims to bridge the evidence-practice gap regarding the preventive care process of the risks of pressure ulcers, malnutrition, poor oral health and falls among older persons in nursing homes. The intervention is aimed at end users, lasts for three weeks and is divided into two parts. First, end users obtain knowledge on their own by following written instructions. Second, they meet, interact and discuss the knowledge acquired during part one.

Conclusion: The intervention is robustly developed and thoroughly described. The study highlights the extensive process that is necessary for developing tailored complex interventions. The description of the entire development process may enhance the replicability of this intervention. The intervention needs to be tested and evaluated in an upcoming feasibility study.

Trial number: Clinical Trial NCT05308862.

For peer review only - http://bmjopen.bmj.com/site/about/guidelines.xhtml

Strengths

- Inspired by the Medical Research Council guidelines for complex intervention, a robust development process was undertaken based on the literature and research conducted in the local context prior to developing the complex intervention.
- A complex intervention was codesigned both with and for nurse aides, registered nurses and managers in workshops. Additionally, key persons working in the municipality were engaged in the development of this tailored intervention.
- To bridge the evidence-practice gap regarding the risks of pressure ulcers, malnutrition, poor oral health and falls among older persons in nursing homes, knowledge translation strategies were applied during the development process in accordance with the action part of the knowledge-to-action framework.
- A thorough description of the entire development process may enhance the replicability of the current intervention.

Limitations

- One limitation of the development process was that this design is time- and resource-consuming. On the other hand, this was necessary to develop a tailored complex intervention that might enhance the likelihood of successful implementation. The transferability of the tailored intervention to other nursing homes might also be a limitation.

For peer review only - http://bmjopen.bmj.com/site/about/guidelines.xhtml

Introduction

There remains an evidence-practice gap in preventing the risks of pressure ulcers, malnutrition, poor oral health and falls among older persons in nursing homes[1 2]. These health risks cause a major burden for older persons[3] and they are costly for the health care system[4]. Since older persons are more vulnerable to these health risks[5] and considering the increasing ageing population globally, particularly with regard to older persons aged 80 years or older[6], evidence-based preventive work is crucial to manage this demographic challenge and, importantly, these health risks among older persons.

In Sweden, there is a national quality register, Senior Alert, providing an individualized, standardized, structured and systematic preventive care work process for older persons 65 years or older who are at risk of pressure ulcers, malnutrition, poor oral health and falls[7]. Senior Alert provides evidence-based knowledge aimed at preventing these health risks to enable a healthy ageing among older persons[8]; in addition, it can increase cost efficiency[9]. However, a lack of knowledge among those working with older persons has been identified as one major challenge regarding to preventive work[2 10]. As a result, these health risks continue to be prevalent[7]. For instance, approximately every third older person living in a nursing home faces at least one of these health risks, and every tenth older person faces all four of these health risks[1]. Additionally, not all older persons who are at risk have planned care interventions[11 12] and there is a mismatch between identified risks and planned and performed care interventions[13 14], thus indicating an evidence-practice gap and consequently, highlighting the urgent need of translating knowledge into practice.

Nevertheless, this is not unique to Sweden or this context; in contrast, health systems worldwide face the shared challenge of translating knowledge into practice[15]. Knowledge translation has been defined as *"a dynamic and iterative process that includes synthesis, dissemination, exchange and ethically sound application of knowledge to improve health care of people in the country, provide more effective health service and products and strengthen the health care system",* p. 165[16]. Ineffective knowledge translation can result in an evidence-practice gap[17] and, worryingly, lead to situations in which patients are denied interventions that have been proven to be beneficial[18], which in turn can result in a reduction in their quality of life[19].

For peer review only - http://bmjopen.bmj.com/site/about/guidelines.xhtml

To bridge this evidence-practice gap, conceptual frameworks are recommended[20]. The knowledge-to-action (KTA) framework is intended to help the parties involved in the process of knowledge translation[18]. The KTA framework is also appropriate when addressing an evidence-practice gap[15] and conducting pragmatic research[18].

As a part of translating knowledge into practice and promoting knowledge use by end users[21], the engagement of both researchers and stakeholders in research is crucial[22]. Engaging stakeholders at an early stage in the development of solutions that can be applied to real world settings is essential according to the Medical Research Council´s (MRC) framework for complex interventions[23]. Complex interventions have multiple components, target multiple groups or levels of an organization and attempt to affect multiple outcomes[23]. Additionally, for complex interventions to be most useful to end users, the local context must be taken into account[24]. Since it is well underpinned that organizational factors hinder preventive work in nursing homes[2 25], considering and understanding the local context and integrating it into the process of intervention development is crucial[26].

Consequently, change in the practices of nursing homes is considered to be complex[27], but if complex interventions are tailored to the local context[28], including the targets of the intervention[23 24] and is directly relevant to them[29], such interventions could be successful.

Aim

The aim of this study was to describe the development of a codesigned complex intervention intended to prevent pressure ulcers, malnutrition, poor oral health and falls among older persons in nursing homes.

Methods

Definitions

Nursing homes were defined based on the definition provided by Neziraj et al. (2021)[1]: residential care homes where older persons live and receive municipal health care.

Health care personnel and managers were defined based on the definition provided by Neziraj et al. (2021) as follows[2]:

Nurse aide: a person with a secondary degree in nursing, involves three years of study in high school *or* a person without any formal education in nursing.

For peer review only - http://bmjopen.bmj.com/site/about/guidelines.xhtml

Registered nurse: a person with a bachelor's degree in nursing, which involves three years of study at university.

Manager: a person who is in charge of nurse aides or registered nurses.

End users: nurse aides, registered nurses and managers working in nursing homes.

Stakeholders: key persons working in the municipality who are considered to play an important role in the development and implementation of the intervention.

Study context and setting

In nursing homes, nurse aides are the main providers of care and services and are on duty around the clock. Nurse aides work under the regulations of the Social and Services Act (SFS)[30] but are also delegated tasks according to the Health and Medical Services Act (HSL)[31], usually by registered nurses. Registered nurses guide care in nursing homes and work under the regulations of HSL[31]. In the current setting, a large town located in southern Sweden with 39 nursing homes, one registered nurse (or occasionally more depending on the size of the nursing home) is located in the nursing home during office hours but is also available at any other time. Managers who are in charge of the care and services provided by the nurse aides are located at their respective nursing homes during office hours.

For transparency, the research group (n=5) positions are reported; four of the researchers hold positions as either doctoral students (MN), associated professors (MAX), professors (CK) or senior lectures (PH) at the affiliated university. The last author (MA) is a PhD and hold the position as a research and development coordinator in the municipality where the study was conducted. All the authors are registered nurses, and two of them (MN, MA) specialize in elderly care and have worked in this context previously,

In addition, a reference group was created, which consisted of experts (n=7) drawn from the local context; nurse aide (n=1), managers in charge of nursing homes (n=2), head of managers in charge of registered nurses (n=1), development managers (n=2) and head of the nursing homes in the municipality (n=1).

Study design

The current study describes the development of a codesigned complex intervention and is a part of the PROSENIOR program (https://mau.se/en/research/projects/prosenior/). This part of the PROSENIOR program aims to develop, test and evaluate a codesigned complex

For peer review only - http://bmjopen.bmj.com/site/about/guidelines.xhtml

intervention to prevent pressure ulcers, malnutrition, poor oral health and falls among older persons living in nursing homes in a two-arm pragmatic cluster randomized trial. The randomization was conducted with the double aim to first develop the intervention and then to evaluate the feasibility in the nursing homes allocated to the intervention group. The current study only reports on the intervention development part of this trial. The feasibility evaluation regarding e.g. recruitment and retention of nursing homes and randomization procedure will be reported separately elsewhere. In the current study, the randomization aimed to invite end users allocated to the intervention arm to develop a codesigned complex intervention. The control arm was therefore not included in the current study. The nursing home is the cluster and the unit of allocation. The nursing homes were randomized using a computerized program (Excel) by MN to either intervention or control arm. MN informed the managers in the included nursing homes about allocation output. Due to the nature of the design, the cluster randomization of nursing homes was unblinded to the nursing homes and the researchers (Figure1).

The development of the codesigned complex intervention (hereafter called the intervention) was conducted in three phases. The phases are described below. The development of the current intervention was conducted in a pragmatic paradigm as it is intended to work in a real-world setting [29]; this process was inspired by the MRC guidelines for complex interventions[24], applied the KTA framework[18] and engaged end users and stakeholders in the process of codesign[32].

We follow the guidance for reporting intervention development studies (GUIDED)[33] when describing the development of the intervention and the template for intervention description and replication (TIDieR) checklist and guide[34] when describing the intervention. We use "development" to refer to the whole process of intervention development and "design" to indicate the intervention content, format and delivery.

Patient and public involvement

Patients or informal caregivers were not involved in the research process. End users codesigned the intervention with the research group in workshops. Stakeholders were also involved in this research; they supported the research group throughout the entire development of the intervention by contributing their valuable knowledge. All engagement is described in detail in the section "Development of the intervention" below.

For peer review only - http://bmjopen.bmj.com/site/about/guidelines.xhtml

Development of the intervention

We developed the intervention in three phases and applied the KTA framework in all phases (Figure 2).

Theory

The KTA framework takes implementation strategies into account already in the development phase[18], which promotes and sustains practice change[15]. We applied the KTA framework because it offers a structured and systematic approach to translate knowledge into practice[18]. It comprises two parts: knowledge creation and the action cycle. Since evidence-based knowledge is already available to end users in the quality register Senior Alert, the action cycle was applied during the development of the current intervention. The action cycle consists of the following steps: *1. Identify the problem, identify and review selected knowledge, 2. Adapt knowledge to the local context, 3. Assess barriers to knowledge use, 4. Select and tailor implementation strategies, 5. Monitor knowledge use, 6. Evaluate the outcomes* and *7. Sustain knowledge use*[18 35]. Steps 1-4 the action cycle were applied throughout the development process of developing the intervention in an iterative, dynamic and permeable way.

Phase one

During this phase, we established contact with stakeholders in the municipality, updated us of the current status of the literature in this area and conducted studies in the local context.

Establishing contact with stakeholders in the municipality

Initially, we established contact and met with the head of the nursing homes in the municipality. The reference group was created in this phase (described in the paragraph "Study context and setting" above).

Searching for literature and conducting studies in the local context

As a part of step 1 in the KTA framework*, identify the problem, identify and review selected knowledge*, firstly, we updated us of the current status of the literature regarding prevention of pressure ulcers, malnutrition, poor oral health and falls and intervention studies in this area. Subsequently, we conducted a cross-sectional study to determine the prevalence of the risks of pressure ulcers, malnutrition, poor oral health and falls in nursing homes in southern Sweden[1].

For peer review only - http://bmjopen.bmj.com/site/about/guidelines.xhtml

As a part of steps 2-3 in the KTA framework, *adapt knowledge to the local context and assess barriers to knowledge use,* we conducted focus group interviews (n=5) with end users (n=21) who worked in nursing homes to prevent pressure ulcers, malnutrition, poor oral health and falls[2]. The focus group interviews lasted between 63 and 106 min (mean 83 min). A detailed description of this study and its participants is provided in Neziraj et al[2]. Additionally, we asked the end users included in our previous study[2] how an optimal intervention could be designed to prevent the risks of pressure ulcers, malnutrition, poor oral health and falls among older persons in nursing homes. These particular data were targeted for the current study. Hence, these data were not reported in our previous study, but are included, analysed and reported in our current study.

Phase two

During this phase, we recruited and randomized nursing homes. Subsequently, we invited end users in the intervention arm to participate in workshops, and planned and conducted the workshops. We also analysed the specific data regarding intervention design drawn from the focus group interviews (see the previous paragraph on phase one for clarification) and the workshops.

Recruiting and randomizing nursing homes

In this part of the two-arm pragmatic cluster randomized trial, randomization aimed to recruit end users in nursing homes allocated to the intervention arm to codesign an intervention together with the research group in workshops.

Inclusion criteria for the study were nursing homes working with and registered in the quality register Senior Alert. We recruited eligible nursing homes (n=21) to participate in the study via digital meetings. In total, eight nursing homes agreed to participate and were cluster randomized using a computerized program to either the intervention (n=4) or control arm (n=4). Subsequently, we invited end users (n=118) working in nursing homes in the intervention arm to participate in workshops intended to develop a tailored intervention together with the research group; the invitations were extended both via a digital information video and in written form. The remaining end users (n=184) working in the nursing homes who were allocated to the control arm continued with their usual care routine.

Conducting workshops

For peer review only

For peer review only - http://bmjopen.bmj.com/site/about/guidelines.xhtml

As a part of steps 2-4 in the KTA framework, *adapt knowledge to the local context, assess barriers to knowledge use* and *select and tailor implementation strategies*, we conducted workshops with end users. In total, four workshops were conducted, which featured two nurse aides, one registered nurse and one manager in each workshop; the workshops were conducted over the course of four weeks (March-April 2022). The workshops were kept small to offer the end users the possibility of exhibiting activity and creativity[36]. The first author (MN) led the workshops together with one of the coauthors (all coauthors participated in one workshop each). The workshops were intended to serve as a place in which participants could learn together and discuss the design of the intervention in four different stations (Table 1). The end users engaged in active discussion and wrote creative ideas and suggestions on the walls and the board in a lecture hall designed for the purpose of encouraging creative pedagogy. In the first station, the end users were asked to discuss the risks of pressure ulcers, malnutrition, poor oral health and falls and the care interventions that should be applied. In the second station, they were asked to discuss and identify barriers and facilitators they had encountered in their own work regarding the preventive care process stipulated by Senior Alert (identify a risk, assess causes and plan, undertake and evaluate care intervention). Barriers were written down on pink post-it notes, while facilitators were written down on green post-it notes. These post-it notes were subsequently placed at the appropriate location on the board with regard to the predawn preventive care process. The focus of the discussions at station three was on the end users´ needs and the support they needed throughout the preventive care process. In the fourth station, they were asked to discuss the core components of the intervention, how to provide follow-ups and implementation strategies. After completing each workshop, MN photographed and briefly summarized the written data from each station. This summary was used if the end users in the subsequent workshop reached an impasse and/or discussed and wrote similar suggestions and ideas to those proposed by the end users in the previous workshop. Each workshop lasted for three hours, and the discussions were audio recorded to support the written data collection during the analysis.

For peer review only - http://bmjopen.bmj.com/site/about/guidelines.xhtml

1 **Table 1.** Workshop content (n=4).

Workshop	Station	Content	Examples of questions to discuss
Workshop 1-4	Station 1	Case regarding an older person at risk of pressure ulcers, malnutrition, poor oral health and falls living in a nursing home	• What would you have done in this case regarding these four risks?<br>• Are there any good examples? What can you learn from good examples?<br>• What additional knowledge do you need regarding these four risks in order to produce a risk assessment and provide adequate care interventions?
	Station 2	Senior Alert´s care process	• Place green/pink post-it notes on the care process regarding what works/what can be improved in your own work and workplace.
	Station 3	End users needs´ and the support they need regarding preventive work	• What do you need in your preventive work?<br>• Why is this important, and what is most important (rank 1-3)?<br>• Who needs help in the context of preventive work?<br>• Who should be involved and in what way?<br>• What is necessary for it to be feasible?<br>• How can you work better/smarter?<br>• How can you work in a more sustainable way?
	Station 4	Core components of the intervention	• What should be included in the intervention?<br>• Who should it target?<br>• How should it be designed?<br>• How much/often/for how long should the intervention take place?<br>• How should it be followed up?<br>• Where should it be implemented?<br>• How should it be implemented?

2

3

4

5

For peer review only - http://bmjopen.bmj.com/site/about/guidelines.xhtml

Analysing the data from the focus group interviews and the workshops

The analysis was guided by the six phases of reflexive thematic analysis described by Braun and Clarke[37 38]: *1. Familiarizing with the data, 2. Coding, 3. Generating initial themes, 4. Reviewing the identified themes, 5. Defining and naming the themes* and *6. Producing the report.* Thematic analysis was chosen because it facilitates a flexible analysis process but simultaneously provides researchers with the core skills they need to conduct the analysis.

To familiarize ourselves with the data, MN and MA read the transcripts from the focus group interviews, including the data specifically collected for the current study, and the written data collected from the workshops. In addition, MN listened to all the audio-recorded discussions from the workshops meticulously. During the process of reading the data, MN and MA reflected on and generated initial codes. Subsequently, MN and MA met and discussed these initial codes (1). Thereafter, MN and MA separately engaged in a process of identifying and coding entities of interest in relation to the design of the intervention, giving equal attention to all the data (2). The initial codes were then sorted into their core components in relation to the design of the intervention (3). Next, the core components were reviewed by MN to determine whether any relevant data regarding the design of the intervention had been missed (4). Subsequently, MN designed an outline of the intervention. This outline contained the intervention's proposed design, including its content, format, plan for delivery and duration. In the following step of the analysis, the entire research group met and discussed the design of the outline of the intervention. During this step, MN continuously revised the outline of the intervention following discussions within the research group (5). Then, the outline of the intervention was redesigned by MN. The redesigned outline of the intervention was then presented to the research group before it was presented to the stakeholders. The process of producing the final design of the intervention is described in phase three below (6).

Phase three

As part of steps 2-4 in the KTA framework, *adapt knowledge to the local context, assess barriers to knowledge use* and *select and tailor implementation strategies,* MN and MA met regularly with stakeholders in structured meetings to present and discuss the outline of the intervention. MN documented all the meetings. MA works within the municipality and thus facilitated contact with stakeholders who were considered to play an important role in this part of designing the intervention. Since this part of the process was dynamic and iterative and because all relevant uncertainties had not been addressed in the redesigned outline of the

For peer review only - http://bmjopen.bmj.com/site/about/guidelines.xhtml

intervention, it was helpful to meet stakeholders for the purpose of identifying and addressing the remaining uncertainties regarding the content, format, delivery and duration of the intervention. This part of the process was time-consuming and required a back-and-forth process involving meetings and discussions between MN and MA, within the entire research group and with the stakeholders. Next, the redesigned outline of the intervention was adjusted by MN in accordance with the results of these meetings and discussions (Figure 3). Finally, MN investigated whether any data from the focus group interviews and the workshops had been missed, since these data were intended to serve as the foundation for designing the final outline of the intervention. The final design of the intervention, the STAIR OF KNOWLEDGE (Figure 4), is described below.

Results

Findings from our previous studies[1 2] in phase one showed that the prevalence of the risk for pressure ulcer, malnutrition, poor oral health and falls is still high in the local context. Furthermore, findings from phase one suggested that individuals working with older persons in nursing homes need increased knowledge concerning how to prevent these health risks. Since existing evidence and knowledge concerning how to prevent these health risks is already contained in Senior Alert, the challenge seems to lie in the evidence-practice gap. Consequently, in phase two and three, a tailored intervention was codesigned with end users, stakeholders and the research group to reduce the evidence-practice gap. The final design of the intervention is presented below.

A majority of the end users (n=16) in workshops (n=4) were women (n=13), between the ages of 28-63 years (mean 53), and had worked for 3-41 years (mean 18). The meetings (n=13) with stakeholders (n=12) lasted between 60-180 min (mean 134 min).

The final design of the intervention

The final design of the intervention was described in line with the TIDieR checklist[34] (Supplementary file).

The STAIR OF KNOWLEDGE consists of *the foundation and stairs 1-6,* lasts for three weeks and is divided into two parts. Part one, including *the foundation and stairs 1-5*, takes place throughout the entire intervention period (weeks 1-3) and is delivered digitally to end users in the nursing homes via their workplace email addresses. Part two includes *stair 6* and

For peer review only - http://bmjopen.bmj.com/site/about/guidelines.xhtml

takes place during the last week of the intervention period (week 3) in the nursing homes in question (Figure 4).

The content of the intervention

Part one of the intervention: the foundation and stairs 1-5

End users emphasized uncertainties of different professionals' responsibilities regarding the preventive work. For instance, they highlighted that it is highly relevant for respective professional to know "who does what" regarding the preventive working. Hence, *the foundation* is intended to facilitate for different professionals regarding responsibilities for respective profession and working routine in the local context. *The foundation* provide end users with knowledge and awareness of how to work preventively in the context of an existing local working routine and is intended to represent "the ground to stand on".

Furthermore, end users expressed a need of increased knowledge regarding the health risks and the entire preventive working process. They stressed the importance of basic knowledge when working with older persons in nursing homes. According to end users, not all of them has basic knowledge in how to prevent these health risks among older persons. This was particularly common among temporary workers. To meet their need, *stairs 1-4* provide the end users with general knowledge about risks of pressure ulcers, malnutrition, poor oral health and falls according to the care process suggested by the quality register Senior Alert *(stair 1),* risk assessment instruments *(stair 2),* the underlying causes of these risks *(stair 3)* and preventive care interventions (*stair 4)*. *Stairs 1-5* provide end users with website links that allow them to both read texts and watch videos. *Stairs 1-4* are mandatory for all professionals. *Stair 5* provides end users with knowledge of how to register in the quality register Senior Alert and is mandatory only for users who have access to and the responsibility to register in the quality register Senior Alert.

Part two of the intervention: stair 6

Although it was necessary for end users to increase their knowledge on their own regarding the preventive work, they particularly highlighted the importance of physical meetings. This was also stressed as important by stakeholders and was considered as a complement to the first part of the intervention. Therefore, in part two, *stair 6*, a facilitator (MN) meets with end users to interact with them and discuss the knowledge acquired during part one. The meetings will be structured including discussions based on different cases related to pressure ulcers,

For peer review only - http://bmjopen.bmj.com/site/about/guidelines.xhtml

malnutrition, poor oral health and falls. End users will also perform risk assessments, identify the underlying causes and plan accurate care interventions based on these cases. Additionally, end users will identify environmental risk factors related to the risks of pressure ulcers, malnutrition, poor oral health and falls in their own workplace. They will also discuss and generate ideas how to follow up on the preventive care process on an organizational level. This part of the intervention is intended to inspire end users to prevent pressure ulcers, malnutrition, poor oral health and falls among older persons in nursing homes.

The format of the intervention

Part one of the intervention: the foundation and stairs 1-5

From end users' perspective, it was important with a clear format. They expressed a need of a structured, readable and colourful working "manual". Hence, the format of the intervention is designed as colourful stair with the intention to visualize the entire preventive working process. To enhance the structure, end users are provided with written instructions in respective stair. Furthermore, stakeholder emphasized the need of a "self-check box" for end users when completing the foundation and stairs in the intervention. Stakeholder believed that this could increase participation and involvement among end users. Since both end users and stakeholders stressed that there are many end users that do not have the Swedish language as their native language, the language is adjusted to suit the local context. Furthermore, end users expressed that the format of the intervention should consider different ways of learning. This was also highlighted as important by stakeholders. Hence, the format consist of both reading texts and watching videos. Moreover, end users and stakeholders emphasized that a digital intervention could be a sustainable solution.

Part two of the intervention: stair 6

End users and stakeholder were in agreement that it is necessary to meet and discuss. Therefore, in part two of the intervention, end users meet in their respective nursing home. Also, the format of this part of the intervention was designed as an inspiration to raise awareness of the preventive work among end users.

The delivery of the intervention

Part one of the intervention: the foundation and stairs 1-5

For peer review only - http://bmjopen.bmj.com/site/about/guidelines.xhtml

The intervention will be delivered via email to managers in nursing homes. Subsequently, respective manager will forward the intervention via workplace email addresses to nurse aides and registered nurses. The end users highlighted that some learn better individually while others learn better in group. Therefore, they are permitted to choose if they want to read texts and watch videos individually and/or in group. *The foundation and stairs 1-5* is anticipated to take approximately 10 minutes, 60 minutes, 20 minutes, 10 minutes, 30 minutes and 60 minutes respectively for end users to complete. End users can choose to complete this part of intervention at once or divide it during working hours.

Part two of the intervention: stair 6

Part two of the intervention will be delivered by a facilitator (MN) who will moderate sessions lasting approximately 30 minutes each, Monday-Friday, in the nursing homes in question. If end users participate in all the sessions during this week, the planned amount of time is two and half hours for each end user.

Discussion

The current codesigned complex intervention, the STAIR OF KNOWLEDGE, was developed together with end users in workshops in an active and creative way. Stakeholders were also engaged in an iterative and dynamic way throughout the development of the intervention, as an important part of undertaking implementation strategies already in the development phase[39]. As recommended by the MRC framework[24], we meticulously considered the relationship between the intervention and its context when developing the intervention. Furthermore, we followed the strategies for knowledge translation included in the KTA framework[18]. Hence, the strengths exhibited by the development of this complex intervention lie in the fact that it was developed both together with and for end users and engaged stakeholders who are considered to play an important role in the development and implementation process. The current intervention is intended to work in a real-world setting and aims to bridge the evidence-practice gap regarding the process of preventing the risks of pressure ulcers, malnutrition, poor oral health and falls; ultimately, this intervention may reduce these risks among older persons in nursing homes.

When developing new intervention, the value of the used design process cannot be understated[40]. In fact, engagement of end users in a creative environment have been linked to more robust research and development efforts, which in turn may drive more successful

For peer review only - http://bmjopen.bmj.com/site/about/guidelines.xhtml

interventions outcome[40]. Hence, the benefits of codesign are potentially substantial[41]. For instance, engaging end users and stakeholders as design partners to the research group could ensure that the intervention exhibits a better fit to their needs[32]. Engaging end users and stakeholders early enables their experiences and requirements to be taken into account at the start rather than a situation in which the researchers presume to know what is required[39]. In the current development process, although end users´ and stakeholders´ engagement ranged in intensity from relatively passive to highly active, their engagement pervaded the entire development process, and important decisions regarding the intervention design were made by considering their input. Furthermore, because we engaged end users and stakeholders, the current intervention was based on their own experiences regarding the evidence and knowledge that are necessary throughout the entire process of preventing the risks of pressure ulcers, malnutrition, poor oral health and falls. Engaging end users and stakeholders during the developing process[42] was also important in light of the local context since this enabled us to identify facilitators and barriers in the environment in which the intervention will eventually be implemented[26].

A recent scoping review investigating education interventions for health professionals on fall prevention in health care settings[43], highlighted that health professional education to prevent fall is important. Nevertheless, the scoping review concluded that there are no uniform education design principles utilized to date[43]. Another review found that it was uncertain whether education delivered in different format such as didactic or video-based format makes a difference to health professionals´ knowledge of pressure ulcers prevention. However, education format in the current developed intervention was designed to fit end users' needs and suit the local context, which may have benefits for the outcome.

Considering and understanding the local context is also crucial when addressing an evidence-practice gap[24]. In this case, knowledge concerning the process of preventing the risks of pressure ulcers, malnutrition, poor oral health and falls is already contained in the quality register Senior Alert, but this evidence has not been fully translated into practice. Thus, we focused on translating the existing knowledge contained in Senior Alert into practice. However, if this knowledge is to be implemented effectively[44], it is crucial to employ a conceptual framework[20]. Therefore, we chose the KTA framework because it provided us with knowledge translation strategies to reduce the evidence-practice gap[18], and it was suitable since the quality register Senior Alert is already in use. Furthermore, adapting

For peer review only - http://bmjopen.bmj.com/site/about/guidelines.xhtml

knowledge to the local context and assessing barriers to knowledge use may enable the research to have a greater impact[45], which could in turn reduce the evidence-practice gap.

Successful intervention development is characterized as rigorous and scientific and leads to an intervention that can be implemented in a real-world setting[33]. The robust research process used to develop the STAIR OF KNOWLEDGE intervention incorporates existing evidence, the views of end users and stakeholders[41], the local context and knowledge translation strategies. Consequently, the use of knowledge translation strategies and the engagement of end users who are embedded in the local context in the development of a tailored complex intervention both for and with them could contribute to increased knowledge and awareness of the entire process of preventive care. This may, in turn, reduce the evidence-practice gap among end users and, importantly, reduce the risk of pressure ulcers, malnutrition, poor oral health and falls among older persons in nursing homes. Furthermore, the engagement of stakeholders already in the development process is likely to facilitate the implementation of the current intervention.

Limitations

Although the development of this complex intervention has been completed, it is important to acknowledge the limitations of the development process. First, only four clusters were included in the development process. Nevertheless, since this part of the trial focused on the development of an intervention rather than its evaluation and because the clusters were recruited pragmatically, the clusters included in the trial could be considered sufficient. Second, although all end users in the intervention arm (n=118) were invited to participate in workshops, only 16 participated. However, different professionals participated in the workshops, and the discussions were energetic, active and creative. Third, although this design is creative and can generate new ideas, it is time- and rescore-consuming for all parties involved. It requires end users and stakeholders to set aside time and expend extra effort in their daily work. For researchers, this process requires careful planning to enable them to coordinate, meet with many different persons repeatedly and be responsive to all parties involved. However, although this design required the expenditure of time and resources, the engagement of end users, stakeholders and researchers is meaningful and necessary to develop successful interventions; ultimately, this design might have an impact on to prevent the risks of pressure ulcers, malnutrition, poor oral health and falls among older persons in

For peer review only - http://bmjopen.bmj.com/site/about/guidelines.xhtml

nursing homes. Furthermore, the current intervention might offer value when used by others and could likely be adjusted to and tested in similar contexts.

Conclusion

The current codesign complex intervention, the STAIR OF KNOWLEDGE, which aims to prevent the risks of pressure ulcers, malnutrition, poor oral health and falls among older persons in nursing homes, is robustly developed and thoroughly described. A careful description of the entire development process and the intervention itself can enhance the replicability of the current intervention. This article highlights the extensive process that is necessary for the development of tailored complex interventions. Finally, this codesigned complex intervention might result in more evidence-based practice concerning the entire process of preventing the risks of pressure ulcers, malnutrition, poor oral health and falls and, importantly, reduce these health risks among older persons in nursing homes. However, uncertainties regarding the intervention itself remain. Thus, the STAIR OF KNOWLEDGE must be tested and evaluated in an upcoming feasibility study before we continue to the stage of conducting a full trial evaluation.

Ethical considerations

This trial was approved by the Swedish Ethical Review Authority (DNR 2019-06414). In addition, written approval was requested and granted by the head of the department of elderly care homes in the municipality in which this trial was conducted. All end users working in eligible nursing homes were invited to participate in the workshops. Moreover, end users had the right to withdraw from participation at any stage without providing reasons and bearing any consequences. Participation in the workshops was based on written consent. The results of this trial may be considered to contribute to scientific value on good ethical grounds, and the benefits of participating in the trial outweigh the corresponding risks.

Funding statement

This research received finical support with regard to conducting the workshops from the Derbrings and Stöltens research and development foundation. The funders had no role in the research manuscript's design, conduct, analysis, interpretation or drafting.

CRediT authorship contribution statement

For peer review only - http://bmjopen.bmj.com/site/about/guidelines.xhtml

Merita Neziraj: Conceptualization, Methodology, Investigation, Writing – original draft, Writing – review & editing, Validation, Formal analysis, Visualization. **Malin Axelsson:** Conceptualization, Methodology, Investigation, Writing – review & editing, Validation, Supervision. **Christine Kumlien:** Conceptualization, Methodology, Investigation, Writing – review & editing, Validation, Supervision. **Peter Hellman:** Conceptualization, Methodology, Investigation, Writing – review & editing, Validation, Supervision. **Magdalena Andersson:** Conceptualization, Methodology, Investigation, Writing – review & editing, Validation, Formal analysis, Supervision. All the authors read and approved the final version of the manuscript.

Competing interests

The authors have no conflicts of interest.

Participant consent

All participation in the workshops was based on written consent.

Acknowledgements

We thank the nurse aides, registered nurses and managers who participated in the development of the intervention. We also thank the stakeholders who participated during this process. Their enthusiastic participation was helpful in developing the STAIR OF KNOWLEDGE intervention. The Derbring and Stölten foundation is acknowledged for its financial support.

Data sharing statement

The data that support the development of the STAIR OF KNOWLEDGE intervention are not publicly available to ensure confidentiality. All data relevant to the development are included in the article. All figures and tables included in this article are original.

For peer review only - http://bmjopen.bmj.com/site/about/guidelines.xhtml

References

1. Neziraj M, Hellman P, Kumlien C, et al. Prevalence of risk for pressure ulcers, malnutrition, poor oral health and falls: a register study among older persons receiving municipal health care in southern Sweden. *BMC Geriatrics* 2021;21(1) doi: 10.1186/s12877-021-02205-x
2. Neziraj M, Andersson M, Hellman P, et al. Prevention of pressure ulcers, malnutrition, poor oral health and falls in nursing homes: A focus group study with nurse aides, registered nurses and managers. *International Journal of Nursing Studies Advances* 2021;3(100056-) doi: 10.1016/j.ijnsa.2021.100056
3. Gorecki C, Brown JM, Nelson EA, et al. Impact of pressure ulcers on quality of life in older patients: a systematic review. *Journal of the American Geriatrics Society* 2009;57(7):1175-83.
4. Marjolein van der V, Juanita AH, Geraerds AJLM, et al. Health care costs of injury in the older population: a prospective multicentre cohort study in the Netherlands. *BMC Geriatrics* 2020;20(1):1-11. doi: 10.1186/s12877-020-01825-z
5. Bravell ME, Westerlind B, Midlov P, et al. How to assess frailty and the need for care? Report from the Study of Health and Drugs in the Elderly (SHADES) in community dwellings in Sweden. *ARCHIVES OF GERONTOLOGY AND GERIATRICS* 2011;53(1):40-45. doi: 10.1016/j.archger.2010.06.011
6. WHO. Ageing well must be a global priority 2014 [accessed 10-23 2020.
7. Trinks A, Hägglin C, Dennis N, et al. The impact of a national quality register in the analysis of risks and adverse events among older adults in nursing homes and hospital wards: a Swedish senior Alertsurvey. *Safety in Health* 2018;4(10) doi: 10.1186/s40886-018-0077-x
8. Edvinsson J, Rahm M, Trinks A, et al. Senior alert: a quality registry to support a standardized, structured, and systematic preventive care process for older adults. *Quality management in health care* 2015;24(2):96-101.
9. Rubenstein LZ, Harker JO, Salvà A, et al. Screening for undernutrition in geriatric practice: developing the short-form mini-nutritional assessment (MNA-SF). *The journals of gerontology Series A, Biological sciences and medical sciences* 2001;56(6):M366-M72.
10. Dalvand S, Ebadi A, Ghanei Gheshlagh R. Nurses' knowledge on pressure injury prevention: a systematic review and meta-analysis based on the Pressure Ulcer Knowledge Assessment Tool. *Clinical, Cosmetic and Investigational Dermatology* 2018;ume 11:613-20.
11. Annelie KGP, Rose-Marie Johansson-Pajala Ph D, Marina Arkkukangas Ph D, et al. Preventing Falls and Malnutrition among Older Adults in Municipal Residential Care in Sweden: A Registry Study. *SAGE Open Nursing* 2021;7 doi: 10.1177/23779608211026161
12. Backlund A, Holmbeck O, Kumlien C, et al. A registry study of nursing assessments, interventions and evaluations according to nutrition for persons living in municipal residential care homes. *Nursing open* 2018;5(3) doi: 10.1002/nop2.144
13. Witt S, Englander E, Kumlien C, et al. Mismatch between risk factors and preventive interventions? A register study of fall prevention among older people in one Swedish county. *International journal of older people nursing* 2018;13(4):e12209.
14. Hopewell S, Adedire O, Copsey BJ, et al. Multifactorial and multiple component interventions for preventing falls in older people living in the community. *Cochrane Database of Systematic Reviews* 2018(7) doi: 10.1002/14651858.CD012221.pub2
15. Field B, Booth A, Ilott I, et al. Using the Knowledge to Action Framework in practice: a citation analysis and systematic review. *Implement Sci* 2014;9:172. doi: 10.1186/s13012-014-0172-2 [published Online First: 2014/11/25]
16. Straus SE, Tetroe J, Graham I. Defining knowledge translation. *Cmaj* 2009;181(3-4):165-8. doi: 10.1503/cmaj.081229 [published Online First: 20090720]
17. Brownson RC, Eyler AA, Harris JK, et al. Getting the Word Out: New Approaches for Disseminating Public Health Science. *J Public Health Manag Pract* 2018;24(2):102-11. doi: 10.1097/phh.0000000000000673

For peer review only - http://bmjopen.bmj.com/site/about/guidelines.xhtml

18. Graham ID, Logan J, Harrison MB, et al. Lost in knowledge translation: time for a map? *J Contin Educ Health Prof* 2006;26(1):13-24. doi: 10.1002/chp.47 [published Online First: 2006/03/25]
19. Davis D, Evans M, Jadad A, et al. The case for knowledge translation: shortening the journey from evidence to effect. *Bmj* 2003;327(7405):33-5. doi: 10.1136/bmj.327.7405.33
20. Bowen S, Graham ID. Backwards design or looking sideways? knowledge translation in the real world Comment on "A call for a backward design to knowledge translation". *Int J Health Policy Manag* 2015;4(8):545-7. doi: 10.15171/ijhpm.2015.71 [published Online First: 20150324]
21. Eljiz K, Greenfield D, Hogden A, et al. Improving knowledge translation for increased engagement and impact in healthcare. *BMJ Open Qual* 2020;9(3) doi: 10.1136/bmjoq-2020-000983
22. Graham ID, Kothari A, McCutcheon C. Moving knowledge into action for more effective practice, programmes and policy: protocol for a research programme on integrated knowledge translation. *Implement Sci* 2018;13(1):22. doi: 10.1186/s13012-017-0700-y [published Online First: 20180202]
23. Craig P, Dieppe P, Macintyre S, et al. Developing and evaluating complex interventions: the new Medical Research Council guidance. *Bmj* 2008;337:a1655. doi: 10.1136/bmj.a1655 [published Online First: 2008/10/01]
24. Skivington K, Matthews L, Simpson SA, et al. A new framework for developing and evaluating complex interventions: update of Medical Research Council guidance. *BMJ-BRITISH MEDICAL JOURNAL* 2021;374:n2061. doi: 10.1136/bmj.n2061
25. Lannering C, Ernsth-Bravell M, Johansson L. Prevention of falls, malnutrition and pressure ulcers among older persons: nursing staff's experiences of a structured preventive care process. *Health & Social Care in the Community* 2017;25(3):1011-20. doi: 10.1111/hsc.12400
26. Beran D, Lazo-Porras M, Cardenas MK, et al. Moving from formative research to co-creation of interventions: insights from a community health system project in Mozambique, Nepal and Peru. *BMJ Global Health* 2018;3(6):e001183. doi: 10.1136/bmjgh-2018-001183
27. Rantz MJ, Flesner MK, Zwygart-Stauffacher M. Improving care in nursing homes using quality measures/indicators and complexity science. *J Nurs Care Qual* 2010;25(1):5-12. doi: 10.1097/NCQ.0b013e3181c12b0f [published Online First: 2009/12/17]
28. Per N, Susanne B. Context matters in implementation science: a scoping review of determinant frameworks that describe contextual determinants for implementation outcomes. *BMC Health Services Research* 2019;19(1):1-21. doi: 10.1186/s12913-019-4015-3
29. Zwarenstein M, Treweek S, Gagnier JJ, et al. Improving the reporting of pragmatic trials: an extension of the CONSORT statement. *BMJ* 2008;337:a2390. doi: 10.1136/bmj.a2390
30. Geurden B. Screening for Malnutrition in Healthcare. Universiteit Antwerpen (Belgium), 2014.
31. Lauque S, Arnaud-Battandier F, Mansourian R, et al. Protein-energy oral supplementation in malnourished nursing-home residents. A controlled trial. *Age & Ageing* 2000;29(1):51-56.
32. Slattery P, Saeri AK, Bragge P. Research co-design in health: a rapid overview of reviews. *Health Res Policy Syst* 2020;18(1):17. doi: 10.1186/s12961-020-0528-9 [published Online First: 20200211]
33. Duncan E, O'Cathain A, Rousseau N, et al. Guidance for reporting intervention development studies in health research (GUIDED): an evidence-based consensus study. *BMJ Open* 2020;10(4):e033516. doi: 10.1136/bmjopen-2019-033516 [published Online First: 20200408]
34. Hoffmann TC, Glasziou PP, Boutron I, et al. [Better Reporting of Interventions: Template for Intervention Description and Replication (TIDieR) Checklist and Guide]. *Gesundheitswesen* 2016;78(3):175-88. doi: 10.1055/s-0041-111066 [published Online First: 20160129]
35. Graham ID, Logan J. Innovations in knowledge transfer and continuity of care. *Can J Nurs Res* 2004;36(2):89-103.
36. Ørngreen R, Levinsen K. Workshops as a Research Methodology. *Electronic Journal of e-Learning* 2017;15(1):70-81.

For peer review only - http://bmjopen.bmj.com/site/about/guidelines.xhtml

37. Braun V, Clarke V. Using thematic analysis in psychology. *Qualitative Research in Psychology* 2006;3(2):77-101. doi: 10.1191/1478088706qp063oa

38. Braun V, Clarke V. Reflecting on reflexive thematic analysis. *Qualitative Research in Sport, Exercise and Health* 2019;11(4):589-97. doi: 10.1080/2159676X.2019.1628806

39. O'Cathain A, Croot L, Duncan E, et al. Guidance on how to develop complex interventions to improve health and healthcare. *BMJ Open* 2019;9(8):e029954. doi: 10.1136/bmjopen-2019-029954 [published Online First: 20190815]

40. Sanders EN, Stappers P. Co-creation and the new landscapes of design. *CoDesign* 2008;4(1):5-18. doi: 10.1080/15710880701875068

41. Turner KM, Rousseau N, Croot L, et al. Understanding successful development of complex health and healthcare interventions and its drivers from the perspective of developers and wider stakeholders: an international qualitative interview study. *BMJ Open* 2019;9(5):e028756. doi: 10.1136/bmjopen-2018-028756 [published Online First: 20190530]

42. O'Cathain A, Thomas KJ, Drabble SJ, et al. What can qualitative research do for randomised controlled trials? A systematic mapping review. *BMJ Open* 2013;3(6):e002889. doi: 10.1136/bmjopen-2013-002889

43. Shaw L, Kiegaldie D, Farlie MK. Education interventions for health professionals on falls prevention in health care settings: a 10-year scoping review. *BMC Geriatr* 2020;20(1):460. doi: 10.1186/s12877-020-01819-x [published Online First: 20201109]

44. Moore JB, Maddock JE, Brownson RC. The Role of Dissemination in Promotion and Tenure for Public Health. *J Public Health Manag Pract* 2018;24(1):1-3. doi: 10.1097/phh.0000000000000691

45. Wolfenden L, Mooney K, Gonzalez S, et al. Increased use of knowledge translation strategies is associated with greater research impact on public health policy and practice: an analysis of trials of nutrition, physical activity, sexual health, tobacco, alcohol and substance use interventions. *Health Res Policy Syst* 2022;20(1):15. doi: 10.1186/s12961-022-00817-2 [published Online First: 20220131]

Figure 1 – Flowchart of the two-arm pragmatic cluster randomized controlled trial. The dashed lines illustrate an upcoming study. The current study focused on the development of the STAIR OF KNOWLEDGE intervention.

Figure 2 - Phases one-three illustrate the process of developing the STAIR OF KNOWLEDGE intervention, which took place between 2019 and 2022. Although the knowledge-to-action (KTA) framework is viewed as a cycle by Graham and colleagues (2006), in this figure, the arrow illustrates the fact that the KTA framework was applied throughout phases one-three of the development process. The KTA framework was applied in an iterative and dynamic way in each phase and is described in detail in the text.

Figure 3 - Note: T=how long the meeting lasted for, reported in minutes. MN=the first author. MA=the last author.
The iterative and dynamic process of designing the final outline of the STAIR OF KNOWLEDGE intervention between April and September 2022, including meetings and discussions with stakeholders. In all the meetings, the first author participated. In addition, in some meetings also the last author participated. The blue arrows illustrate that adjustments were made following each meeting.

Figure 4 - Final design of the STAIR OF KNOWLEDGE intervention.

For peer review only - http://bmjopen.bmj.com/site/about/guidelines.xhtml

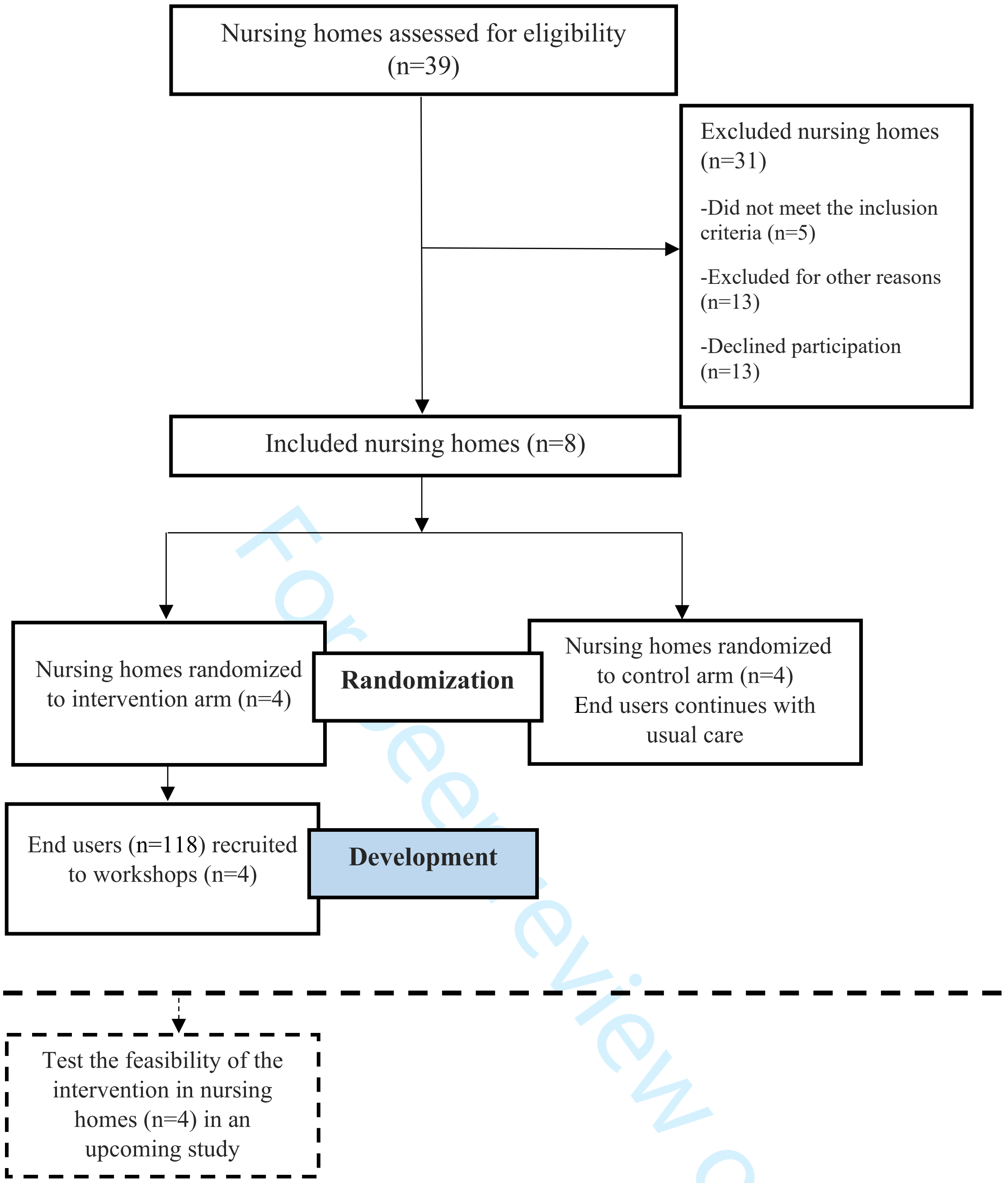

Figure 1. Flowchart of the two-arm pragmatic cluster randomized controlled trial. The dashed lines illustrate an upcoming study. The current study focused on the development of the STAIR OF KNOWLEDGE intervention.

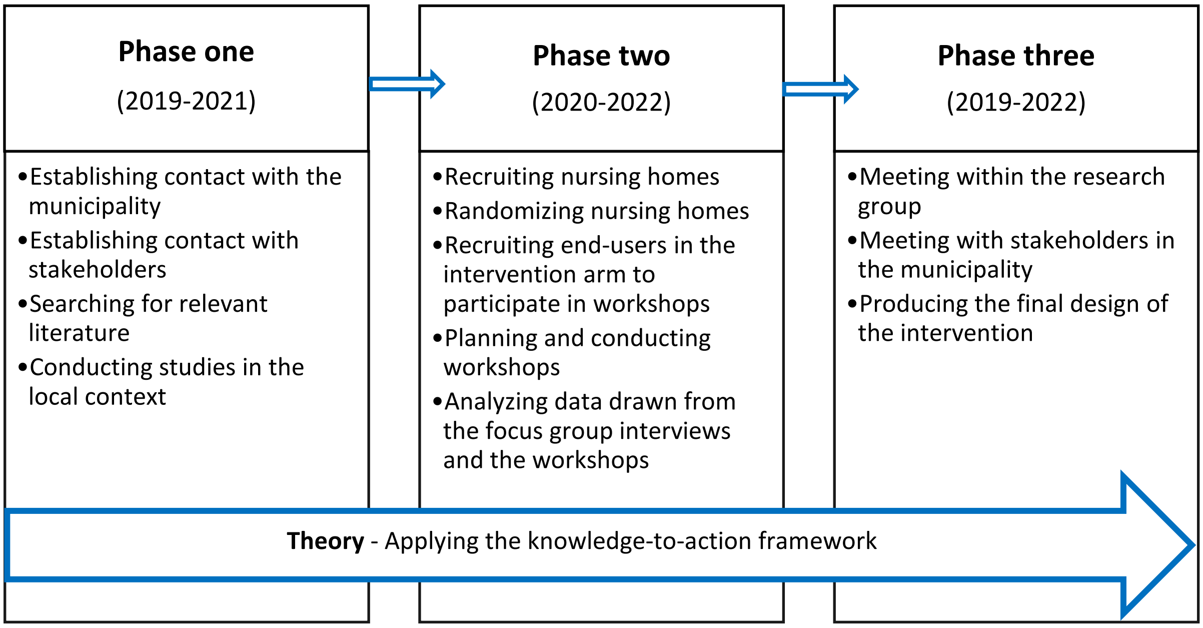

Figure 2. Phases one-three illustrate the process of developing the STAIR OF KNOWLEDGE intervention, which took place between 2019 and 2022. Although the knowledge-to-action (KTA) framework is viewed as a cycle by Graham and colleagues (2006), in this figure, the arrow illustrates the fact that the KTA framework was applied throughout phases one-three of the development process. The KTA framework was applied in an iterative and dynamic way in each phase and is described in detail in the text.

For peer review only

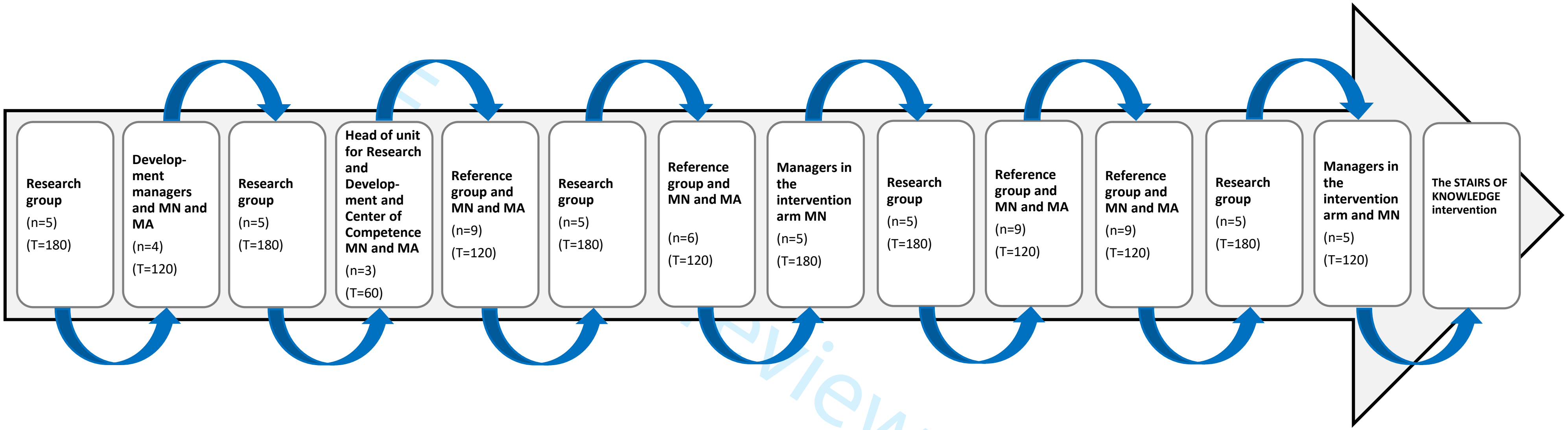

Figure 3. Note: T=how long the meeting lasted for, reported in minutes. MN=the first author. MA=the last author.
The iterative and dynamic process of designing the final outline of the STAIR OF KNOWLEDGE intervention between April and September 2022, including meetings and discussions with stakeholders. In all the meetings, the first author participated. In addition, in some meetings also the last author participated. The blue arrows illustrate that adjustments were made following each meeting.

The STAIR OF KNOWLEDGE

The STAIR OF KNOWLEDGE is addressed to all nurse aides, registered nurses and managers who work with older persons in nursing homes. The STAIR OF KNOWLEDGE aims to increase knowledge regarding the preventive care necessary to prevent the risks of falls, pressure ulcers, malnutrition and poor oral health.

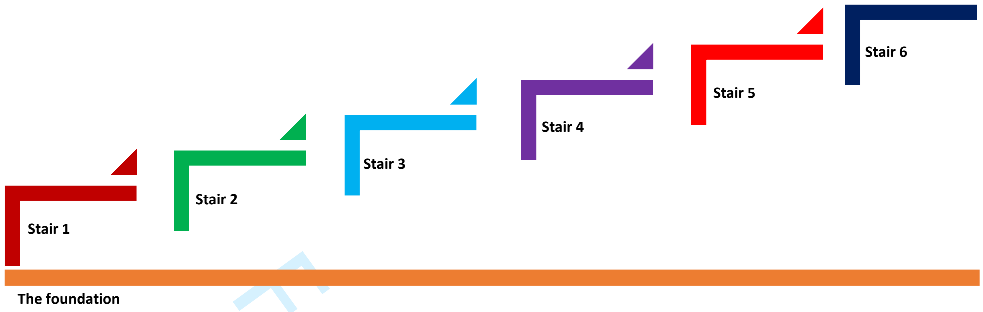

The foundation is mandatory for nurse aides, registered nurses and managers. **Stairs 1-4** and **stair 6** are mandatory for all nurse aides, registered nurses and managers. **Stair 5** is mandatory for individuals who register in Senior Alert. Follow the instructions below.

For your convenience, click the boxes as you progress through the STAIR OF KNOWLEDGE.

- Foundation ☐
- Stair 1a-d ☐
- Stair 2a-d ☐
- Stair 3 ☐
- Stair 4a-d ☐
- Stair 5 ☐
- Stair 6 ☐

The foundation. Local working description of the entire preventive care working process.

- Link to the local working routine.

Stair 1a-d. General information regarding falls, pressure ulcers, malnutrition and poor oral health.

- Links to texts and videos regarding falls, pressure ulcers, malnutrition and poor oral health.

Stair 2a-d. Risk assessment of falls, pressure ulcers, malnutrition and poor oral health.

- Links to texts and videos regarding the risk assessment of falls, pressure ulcers, malnutrition and poor oral health.

Stair 3. Causes of falls, pressure ulcers, malnutrition and poor oral health.

- Link to text regarding the causes of falls, pressure ulcers, malnutrition and poor oral health.

Stair 4a-d. Preventive care interventions for falls, pressure ulcers, malnutrition and poor oral health.

- Links to texts regarding preventive care interventions for falls, pressure ulcers, malnutrition and poor oral health.

Stair 5. Registering in Senior Alert.

- Links to texts and videos regarding how to register in Senior Alert.

Stair 6. Inspiration week.

- Inspiration week focuses on preventive care intended to prevent the risks of falls, pressure ulcers, malnutrition and poor oral health in an inspiring and motivating way. The inspiration week will be organized by and for employees and managers. The inspiration week is preferably organized twice per year.

Figure 4. Final design of the STAIR OF KNOWLEDGE intervention.

TIDieR
Template for Intervention Description and Replication

The TIDieR (Template for Intervention Description and Replication) Checklist*:

Information to include when describing an intervention and the location of the information

Item number	Item	Where located **	
		Primary paper (page or appendix number)	Other † (details)
1.	**BRIEF NAME** Provide the name or a phrase that describes the intervention.	p. 1	
2.	**WHY** Describe any rationale, theory, or goal of the elements essential to the intervention.	p. 4-4 +- 6-8	
3.	**WHAT** Materials: Describe any physical or informational materials used in the intervention, including those provided to participants or used in intervention delivery or in training of intervention providers. Provide information on where the materials can be accessed (e.g. online appendix, URL).	p. 13-16	
4.	Procedures: Describe each of the procedures, activities, and/or processes used in the intervention, including any enabling or support activities.	p. 13-16	
5.	**WHO PROVIDED** For each category of intervention provider (e.g. psychologist, nursing assistant), describe their expertise, background and any specific training given.	p. 5 + 13 + Figure 4	
6.	**HOW** Describe the modes of delivery (e.g. face-to-face or by some other mechanism, such as internet or telephone) of the intervention and whether it was provided individually or in a group.	p. 13-16	
	**WHERE**	p. 13-16	

For peer review only

For peer review only - http://bmjopen.bmj.com/site/about/guidelines.xhtml

**7.**	Describe the type(s) of location(s) where the intervention occurred, including any necessary infrastructure or relevant features.	______________	______________
	**WHEN and HOW MUCH**	p. 13-16	
**8.**	Describe the number of times the intervention was delivered and over what period of time including the number of sessions, their schedule, and their duration, intensity or dose.	______________	______________
	**TAILORING**	p. 13-16	
**9.**	If the intervention was planned to be personalised, titrated or adapted, then describe what, why, when, and how.	______________	______________
	**MODIFICATIONS**	N/A	
**10.‡**	If the intervention was modified during the course of the study, describe the changes (what, why, when, and how).	______________	______________
	**HOW WELL**	N/A	
**11.**	Planned: If intervention adherence or fidelity was assessed, describe how and by whom, and if any strategies were used to maintain or improve fidelity, describe them.	______________	______________
**12.‡**	Actual: If intervention adherence or fidelity was assessed, describe the extent to which the intervention was delivered as planned.	N/A _________	______________

** **Authors** - use N/A if an item is not applicable for the intervention being described. **Reviewers** – use '?' if information about the element is not reported/not sufficiently reported.

† If the information is not provided in the primary paper, give details of where this information is available. This may include locations such as a published protocol or other published papers (provide citation details) or a website (provide the URL).

‡ If completing the TIDieR checklist for a protocol, these items are not relevant to the protocol and cannot be described until the study is complete.

* We strongly recommend using this checklist in conjunction with the TIDieR guide (see *BMJ* 2014;348:g1687) which contains an explanation and elaboration for each item.

* The focus of TIDieR is on reporting details of the intervention elements (and where relevant, comparison elements) of a study. Other elements and methodological features of studies are covered by other reporting statements and checklists and have not been duplicated as part of the TIDieR checklist. When a **randomised trial** is being reported, the

For peer review only

For peer review only - http://bmjopen.bmj.com/site/about/guidelines.xhtml

TIDieR checklist should be used in conjunction with the CONSORT statement (see www.consort-statement.org) as an extension of **Item 5 of the CONSORT 2010 Statement.** When a **clinical trial protocol** is being reported, the TIDieR checklist should be used in conjunction with the SPIRIT statement as an extension of **Item 11 of the SPIRIT 2013 Statement** (see www.spirit-statement.org). For alternate study designs, TIDieR can be used in conjunction with the appropriate checklist for that study design (see www.equator-network.org).

For peer review only

For peer review only - http://bmjopen.bmj.com/site/about/guidelines.xhtml

GUIDED – a guideline for reporting for intervention development studies.

Supplementary File 1: Blank Checklist

Item description	Explanation	Page in manuscript where item is located	Other*
1. Report the context for which the intervention was developed.	Understanding the context in which an intervention was developed informs readers about the suitability and transferability of the intervention to the context in which they are considering evaluating, adapting or using the intervention. Context here can include place, organisational and wider socio-political factors that may influence the development and/or delivery of the intervention (15).	Page 5 (Study context)	
2. Report the purpose of the intervention development process.	Clearly describing the purpose of the intervention specifies what it sets out to achieve. The purpose may be informed by research priorities, for example those identified in systematic reviews, evidence gaps set out in practice guidance such as The National Institute for Health and Care Excellence or specific prioritisation exercises such as those undertaken with patients and practitioners through the James Lind Alliance.	Page 4-5 (Introduction) Page 8-13) Development of the intervention)	
3. Report the target population for the intervention development process.	The target population is the population that will potentially benefit from the intervention – this may include patients, clinicians, and/or members of the public. If the target population is clearly described then readers will be able to understand the relevance of the intervention to their own research or practice. Health inequalities, gender and ethnicity are features of the target population that may be relevant to intervention development processes.	Page 8-13 (Development of the intervention) Page 13 (Results)	
4. Report how any published intervention development approach contributed to the development process	Many formal intervention development approaches exist and are used to guide the intervention development process (e.g. 6Squid (16) or The Person Based Approach to Intervention Development (17)). Where a formal intervention development approach is used, it is helpful to describe the process that was followed, including any deviations. More general approaches to intervention development also exist and have been categorised as follows (3):- Target Population-centred intervention development; evidence and theory-based intervention development; partnership intervention development; implementation-based intervention development; efficacy-based intervention development; step or phased-based intervention development; and intervention-specific intervention development (3). These approaches do not always have specific guidance that describe their use. Nevertheless, it is helpful to give a rich description of how any published approach was operationalised	Page 4-5 (Introduction) Page 6 (Study design)	
5. Report how evidence from different sources informed the intervention development process.	Intervention development is often based on published evidence and/or primary data that has been collected to inform the intervention development process. It is useful to describe and reference all forms of evidence and data that have informed the development of the intervention because evidence bases can change rapidly, and to explain the manner in which the evidence and/or data was used. Understanding what evidence was and was not available at the time of intervention development can help readers to assess transferability to their current situation.	Page 8-13 (Development of the intervention)	
6. Report how/if published theory informed the intervention development process.	Reporting whether and how theory informed the intervention development process aids the reader's understanding of the theoretical rationale that underpins the intervention. Though not mentioned in the e-Delphi or consensus meeting, it became increasingly apparent through the development of our guidance that this theory item could relate to either existing published theory or programme theory	Page 4-5 (Introduction) Page 6 (Study design) Page 7 (Theory)	
7. Report any use of components from an existing intervention in the current intervention development process.	Some interventions are developed with components that have been adopted from existing interventions. Clearly identifying components that have been adopted or adapted and acknowledging their original source helps the reader to understand and distinguish between the novel and adopted components of the new intervention.	The intervention is based on exising evidence-based knowledge in Senior Alert. See figure 4.	
8. Report any guiding principles, people or factors that were prioritised when making decisions during the intervention development process.	Reporting any guiding principles that governed the development of the application helps the reader to understand the authors' reasoning behind the decisions that were made. These could include the examples of particular populations who views are being considered when designing the intervention, the modality that is viewed as being most appropriate, design features considered important for the target population, or the potential for the intervention to be scaled up.	Page 6-10 (Development of the intervention)	

1

For peer review only - http://bmjopen.bmj.com/site/about/guidelines.xhtml

Item description	Explanation	Page in manuscript where item is located	Other*
9. Report how stakeholders contributed to the intervention development process.	Potential stakeholders can include patient and community representatives, local and national policy makers, health care providers and those paying for or commissioning health care. Each of these groups may influence the intervention development process in different ways. Specifying how differing groups of stakeholders contributed to the intervention development process helps the reader to understand how stakeholders were involved and the degree of influence they had on the overall process. Further detail on how to integrate stakeholder contributions within intervention reporting are available (19).	Page 8-13 (Development of the intervention)	
10. Report how the intervention changed in content and format from the start of the intervention development process.	Intervention development is frequently an iterative process. The conclusion of the initial phase of intervention development does not necessarily mean that all uncertainties have been addressed. It is helpful to list remaining uncertainties such as the intervention intensity, mode of delivery, materials, procedures, or type of location that the intervention is most suitable for. This can guide other researchers to potential future areas of research and practitioners about uncertainties relevant to their healthcare context.	Page 12 (Analysed the data from the focus group interviews and workshops) + Page 12 (Third phase)	
11. Report any changes to interventions required or likely to be required for subgroups.	Specifying any changes that the intervention development team perceive are required for the intervention to be delivered or tailored to specific sub groups enables readers to understand the applicability of the intervention to their target population or context. These changes could include changes to personnel delivering the intervention, to the content of the intervention, or to the mode of delivery of the intervention.	Page 12 (Analysed the data from the focus group interviews and workshops) + Page 12 (Third phase) + Page 13 (Results)	
12. Report important uncertainties at the end of the intervention development process.	Intervention development is frequently an iterative process. The conclusion of the initial phase of intervention development does not necessarily mean that all uncertainties have been addressed. It is helpful to list remaining uncertainties such as the intervention intensity, mode of delivery, materials, procedures, or type of location that the intervention is most suitable for. This can guide other researchers to potential future areas of research and practitioners about uncertainties relevant to their healthcare context.	Page 11-12 (Discussion)	
13. Follow TIDieR guidance when describing the developed intervention.	Interventions have been poorly reported for a number of years. In response to this, internationally recognized guidance has been published to support the high quality reporting of health care? interventions[5]and public health interventions[14]. This guidance should therefore be followed when describing a developed intervention.	The description of the intervention followsTIDieR	
14. Report the intervention development process in an open access format.	Unless reports of intervention development are available people considering using an intervention cannot understand the process that was undertaken and make a judgement about its appropriateness to their context. It also limits cumulative learning about intervention development methodology and observed consequences at later evaluation, translation and implementation stages. Reporting intervention development in an open access (Gold or Green) publishing format increases the accessibility and visibility of intervention development research and makes it more likely to be read and used. Potential platforms for open access publication of intervention development include open access journal publications, freely accessible funder reports or a study web-page that details the intervention development process.	Submitted to the BMJ Open	

*e.g. if item is reported elsewhere, then the location of this information can be stated here.

For peer review only

For peer review only - http://bmjopen.bmj.com/site/about/guidelines.xhtml

Duncan E, *et al. BMJ Open* 2020; 10:e033516. doi: 10.1136/bmjopen-2019-033516
